# Photoredox-Driven Three-Component Coupling of Aryl Halides, Olefins, and O_2_

**DOI:** 10.1021/acscatal.3c05988

**Published:** 2024-02-04

**Authors:** Mark C. Maust, Simon B. Blakey

**Affiliations:** Department of Chemistry, Emory University, Atlanta, Georgia 30322, United States

**Keywords:** photoredox, multicomponent coupling, catalysis, olefin functionalization, radical

## Abstract

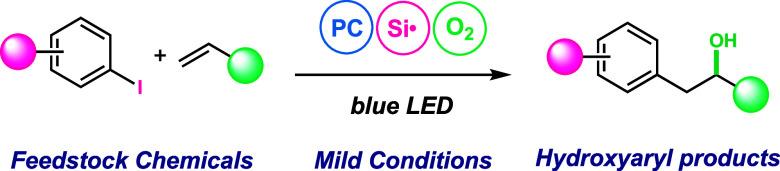

Modern organic synthesis requires methodologies that bring together abundant feedstock chemicals in a mild and efficient manner. To aid in this effort, we have developed a multicomponent radical hydroxyarylation reaction that utilizes aryl halides, olefins, and O_2_ as the reaction components. Crucial to this advance was an oxidative, rather than a reductive, approach to aryl radical generation, which enables reaction tolerance to O_2_. This methodology displays a broad functional group tolerance with a variety of functionalized aryl halides and a broad array of olefins. Development of this methodology enables rapid access to biologically relevant hydroxyaryl products from simple, commercially available starting materials.

Multicomponent coupling reactions, especially those that bring together simple and abundant feedstock chemicals under mild, functional group-tolerant conditions, hold exceptional potential in creating molecular complexity, advancing sustainable synthesis, and generating chemical libraries.^1^ Within this context, a catalytic photoredox-driven union of aryl halides, olefins, and O_2_ to deliver the olefin hydroxyarylation product represents an important and previously unrealized transformation. The development of such a reaction enables rapid access to hydroxyaryl motifs that can be found in natural products, agrochemicals, and pharmaceutical agents (Figure 1, top).^2^

**Figure 1:**
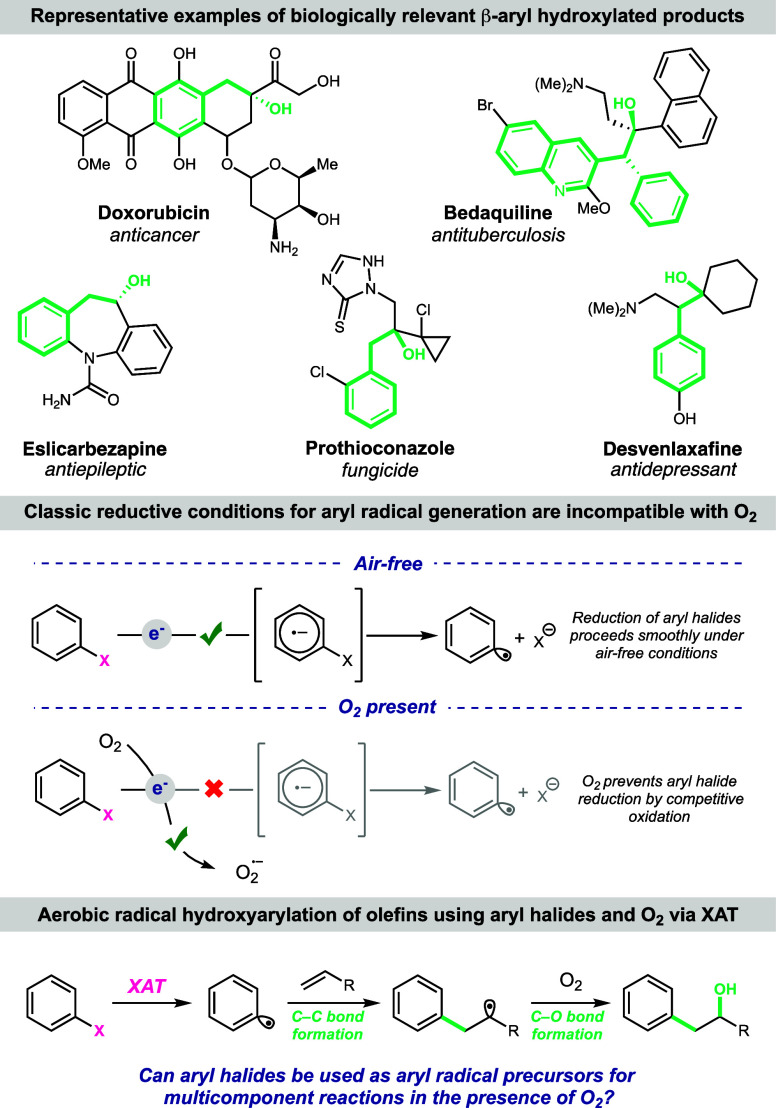
Rational for pursuing a multicomponent radical hydroxyarylation of olefins using aryl halides and O_2_.

The conceptual challenge associated with this transformation is that the generation of aryl radical intermediates from aryl halides is classically considered a reductive process, and the reaction conditions are often incompatible with O_2_ (Figure 1, middle).^3^ Indeed, numerous photoredox reactions that operate in a reductive manifold frequently necessitate air-free conditions to achieve optimal yields.^4^ However, silyl radical-mediated halogen atom transfer (XAT) is an attractive approach to aryl radical generation from aryl halides^5^ and has been shown to tolerate O_2_,^6^ though no instances of utilizing O_2_ as a reaction component for C–O bond formation have been reported to this point.

Based on this observation, we hypothesized that the oxidative activation of silanols would be highly compatible with aryl radical generation and olefin addition in the presence of O_2_.^6a,6b,7^ Furthermore, we recognized that nucleophilic radicals are known to react with triplet O_2_, and the differential nucleophilicity of an sp^2^-aryl radical and an sp^3^-alkyl radical could be utilized for sequencing bond formation in a multicomponent reaction.^8^ We expected the less nucleophilic sp^2^-radical to preferentially react with an olefin substrate, while the resulting more nucleophilic sp^3^-radical is polarity-matched for subsequent capture by O_2_, leading to clean and efficient multicomponent coupling.

Prior work in this area shows several examples of multicomponent olefin oxyarylation reactions, though these reactions require 2,2,6,6-tetramethylpiperidine-1-oxyl (TEMPO) or metal-assisted trapping of the sp^3^-radical intermediate.^9^ To date, only a few reports have described the aryl radical reactivity in the presence of O_2_. Such reported reactions require aryl hydrazines,^10^ aryl diazoniums,^11^ or aryl boronates^9d,12^ as aryl radical precursors and are often combined with stoichiometric metal reductants, limiting their synthetic utility. Prior to this study, no catalytic multicomponent hydroxyarylation of olefins with aryl halides and O_2_ has been reported.

Our reaction design is depicted in Figure 2A. Oxidation of tris(trimethylsilyl)silanol ((TMS)_3_SiOH) by a photocatalyst (PC) initiates a radical Brook rearrangement to silyl radical intermediate **I**. Polarity-matched halogen-atom transfer (XAT) between **I** and aryl halide **II** results in aryl radical **III**. Trapping of the aryl radical with an olefin forges a C–C bond and alkyl radical intermediate **IV**. This radical engages with triplet O_2_ to form the C–O bond and peroxyl radical species **V**, which is reduced by the photocatalyst radical anion to deliver peroxide **VI**. Conversion of **VI** to hydroxy product **VIII** requires an additional equivalent of reductant. We envision that silanol **VII** is formed from the hydrolysis of the halogenated silyl species resulting from XAT of **I** and **II**. We propose that silanol **VII** is oxidized by the excited-state photocatalyst, undergoing a second radical Brook rearrangement and generating another equivalent of photocatalyst radical anion to reduce peroxide **VI** to the alcohol product **VIII.**

**Figure 2:**
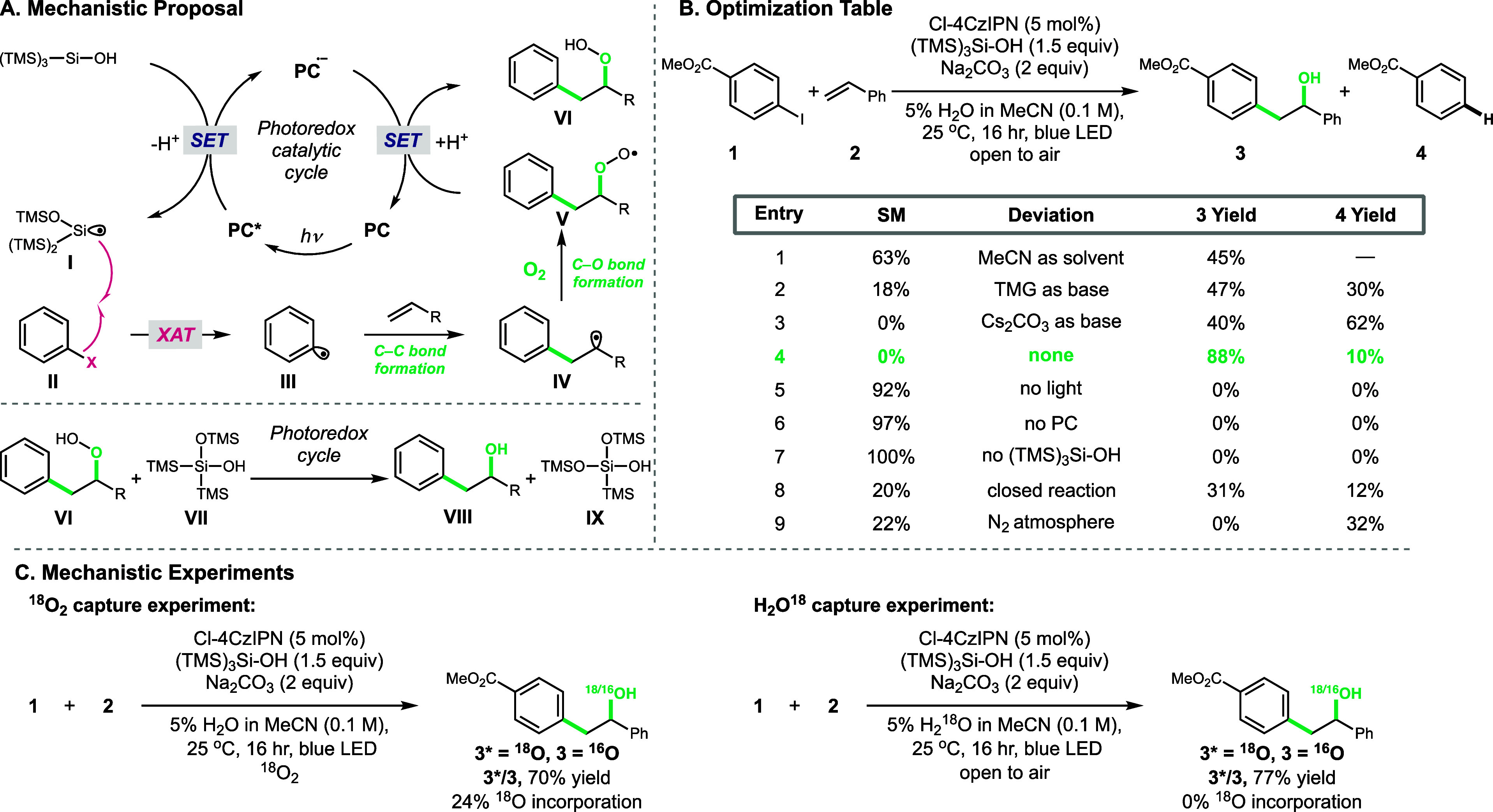
(A) Proposed mechanism for the formation of the observed hydroxyarylation products. (B) Optimization of the hydroxyarylation of olefins with aryl halides and O_2_. Conditions are as follows: **1** (0.1 mmol), **2** (0.3 mmol), Na_2_CO_3_ (0.2 mmol), (TMS)_3_SiOH (0.15 mmol), solvent (0.1 M), blue LEDs, and open to air at room temperature for 16 h. (C) Mechanistic experiments to support radical trapping of O_2_ rather than radical–polar crossover and capture of H_2_^18^O.

With this mechanism in mind, we note that the activation of (TMS)_3_SiOH to form the halogen-abstracting silyl radical requires a highly oxidizing photocatalyst (*E*_p_ = +1.54 V vs SCE),^7^ so we chose a metal-free photocatalyst Cl-4CzIPN (*E*_1/2_^red^ [*PC/PC^•–^ ] = +1.71 vs SCE)^7a^ to begin our studies. In our initial experiment, we investigated the reaction of 4-methyl iodobenzoate as the aryl radical precursor (**1**), styrene as the radical acceptor (**2**), and (TMS)_3_SiOH as the XAT reagent in the presence of the photocatalyst Cl-4CzIPN, and sodium carbonate. Subjecting these reagents to irradiation by blue LEDs in a MeCN solution open to air produced hydroxyarylated product **3** in 45% yield, with the starting material composing the rest of the reaction mass balance (Figure 2B, entry 1). In an effort to push the reaction to complete starting material consumption, we conducted experiments exploring the base solubility, as base is required to generate the silyl radical abstractor. Organic base tetramethylguanidine (TMG) increased the starting material consumption, although a large amount of hydrodehalogenation product **4** was observed (Figure 2B, entry 2). Similarly, cesium carbonate as base led to full consumption of the starting material, but hydrodehalogenation outcompeted hydroxyarylation (Figure 2B, entry 3). Adding water as a cosolvent to the original conditions aided sodium carbonate solubility, facilitating full starting material consumption and increasing the hydroxyarylation yield to 88% with minimal hydrodehalogenation (10%, Figure 2B, entry 4). Control experiments revealed that light, photocatalyst, and XAT reagent are all necessary components for product formation (Figure 2B, entries 5–7).

To probe our hypothesis that C–O bond formation arises from O_2_ capture, we conducted experiments investigating how the reaction atmosphere impacts product formation. Varying the optimized conditions so that the reaction is conducted in a closed reaction vessel rather than open to air reduced the yield of **3** to 31% (Figure 2B, entry 8). Additionally, under an inert N_2_ atmosphere, no **3** was observed. Conducting the experiment under an atmosphere of ^18^O_2_ resulted in a 70% yield of **3** and 3* with 24% ^18^O_2_ incorporation, giving strong evidence to support C–O bond formation resulting from O_2_ capture. To rule out a potential mechanism where the radical intermediate **IV** is oxidized to radical cation and C–O bond formation results in the nucleophilic addition of H_2_O, we replaced the H_2_O cosolvent with H_2_^18^O. No ^18^O 3* was detected by mass spectroscopy, giving further evidence that C–O bond formation arises from radical capture by O_2_.

We next investigated the scope of the reaction, beginning with the scope of aryl iodides as aryl radical precursors. A variety of *para*-substituted electron-poor, -neutral, and -rich aromatics all reacted smoothly in good to excellent yields (Scheme 1, **3**–**19**, 78–96%). Although nitro groups have been reported to be photoactive under blue light irradiation, we obtained a good yield of nitro-containing hydroxyaryl product **6** (62%).^13^ Notably, unprotected phenol **12** was compatible (51%), though increased yields could be obtained with phenol protection (**13**, 87%). Protected aniline **14** reacted smoothly in 77% yield. Given the selectivity for aryl iodides, we explored the reaction tolerance for aromatic substituents that could be utilized for orthogonal reactivity. Pinacol boronate (Bpin)-substituted arene produced a mixture of hydroxyarylated product **15** in 25% yield and hydroxyarylated phenol **12** in 40% yield where **12** likely arises from oxidation and hydrolysis of the aryl-Bpin under photoredox conditions.^14^ Switching from Bpin to the less redox-sensitive *N*-methyliminodiacetic acid (MIDA)-substituted borane increased the yield to 70% of **16**. Electrophilic cross-coupling handles TfO-, Cl-, and Br-substituted arenes were tolerated, and products were obtained in good yields (**17**–**19,** 76–90%). Expanding beyond *para*-substituted arenes, *meta*-substitution was well tolerated with electron-rich, -poor, and reactive handles, all producing good yields (**20**–**22**, 65–95%). Additionally, *ortho*-substitution was well tolerated with both mono- and disubstituted arenes (**23**–**28**, 36–90%). Previous hydroxyarylations have limited utility in functionalizing heteroaryl species.^9−12^ Under our XAT conditions, pyridines could be functionalized at the 2-, 3-, and 4-positions in moderate yields (**29**–**31**, 42–76%). Additional heterocycles such as pyrazine **32**, thiophene **33**, and indole **34** all produced hydroxyarylated products in 51–62% yields.

**Scheme 1:**
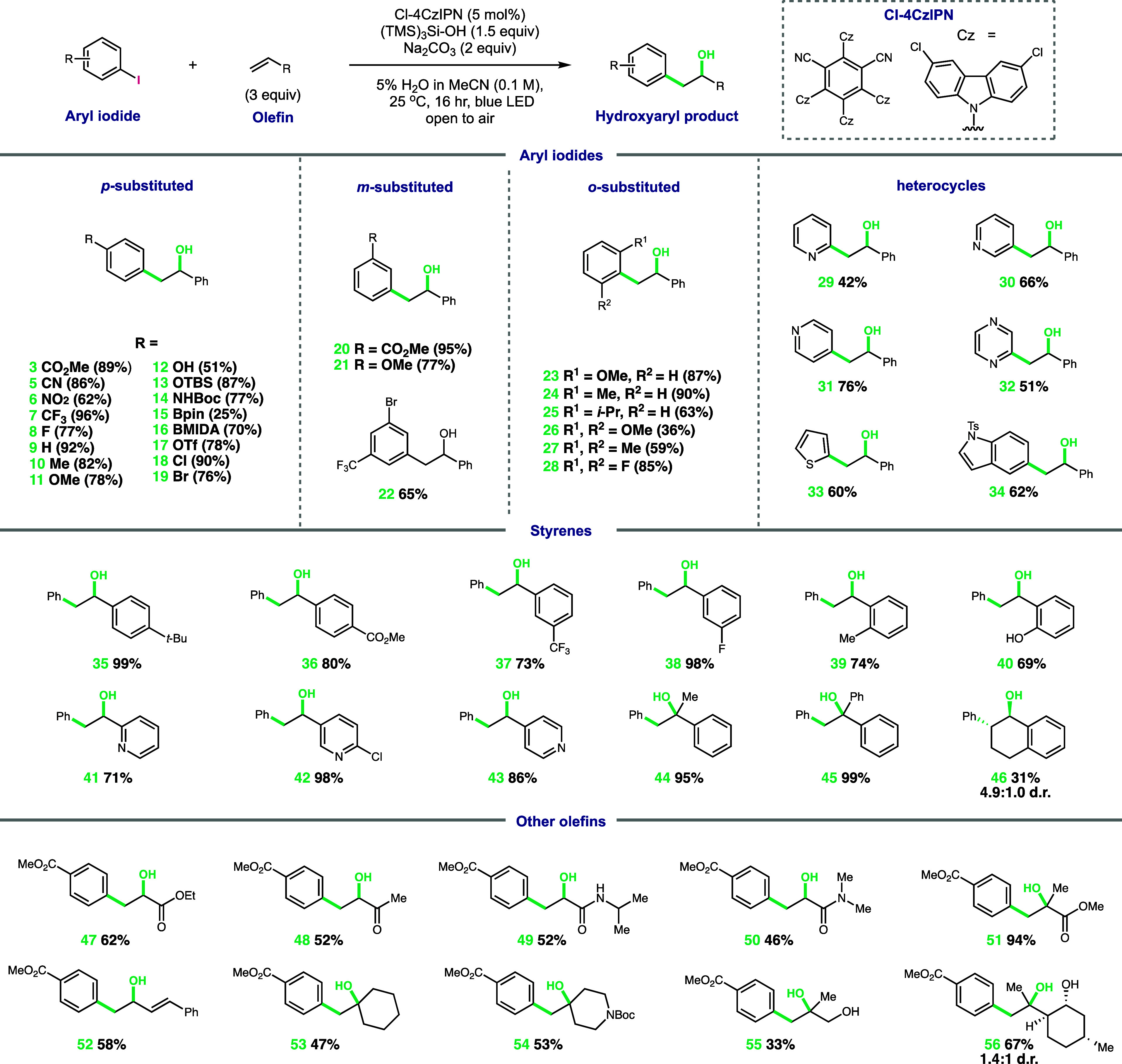
Scope of the Multicomponent Radical Hydroxyarylation of Olefins with Aryl Halides and O_2_ Conditions are as follows: aryl halide (1 equiv), olefin (3 equiv), (TMS)_3_SiOH (1.5 equiv), Na_2_CO_3_ (2 equiv), 5% H_2_O in MeCN (0.1 M), blue LEDs, and open to air at room temperature for 16 h.

With the aryl iodide scope established, we investigated the range of olefin radical acceptors that can be applied to our conditions. We began by exploring styrene substitution and its effect on the hydroxyarylation yield. Electron-poor, -neutral, and -rich styrenes substituted at the *ortho-, meta-, and para*-positions, all gave good to excellent yields, resulting in a variety of benzylic hydroxylated products (**35**–**46**, 74–99%). Again, phenol protection was not required (**40**, 69%), and heterocycles, such as, 2-, 3-, and 4-vinylpyridines, all reacted in good-to-excellent yields (**41**–**43**, 71–96%). Investigating the substitution of the styrene olefin revealed α-substituted styrenes gave excellent yields (**44** and **45**, 95 and 99%, respectively), likely associated with the increased stability of tertiary-substituted radical intermediates. Conversely, β-substitution reduced the hydroxyarylated product yield (**46**, 31%), likely due to additional steric demands at the site of aryl radical addition to the olefin. Expanding beyond styrenes, we investigated the reactivity of Michael acceptors and their ability to generate synthetically useful α-hydroxy carbonyl compounds. Acrylates, enones, and acrylamides were all competent with the α-substitution of the olefin, again increasing the yield (**47**–**51**, 46–94%). Aryl radical addition to a conjugated diene formed secondary allylic alcohol **52** in 58% yield. Additionally, unactivated 1,1-disubstitued olefins reacted to give a variety of tertiary alcohols. Cyclohexane **53**, medicinally relevant piperidine **54**, β-hydroxy alcohol **55**, and functionalized chiral pool substrate **56** (from (−)-isopulegol) were all obtained in moderate yields (33–67%).

To further demonstrate the applicability of this methodology in complex molecule synthesis, we conducted experiments to perform this reaction on scale. Early on in our studies, we observed a decreased yield moving from the optimization scale (0.1 mmol) to the substrate scope scale (0.5 mmol, Table S2). This effect was largely associated with the stir rate, where we observed that smooth “vortexing” of the reaction mixture throughout the reaction time was crucial for optimal and reproducible yields. Scaling from 0.5 to 2 mmol also required slight tuning of the reaction conditions (Table S3). Though the organic photocatalyst Cl-4CzIPN provided good and reproducible yields on scales ≤0.5 mmol, its poor solubility in MeCN/H_2_O became an issue when scaling the reaction, presumably due to reduced light penetration. Alternatively, the photocatalyst [Ir(dFCF_3_ppy)_2_(5,5′-dCF_3_bpy)]PF_6_ has similar oxidizing properties to Cl-4CzIPN (*E*_1/2_^red^ [*Ir^III^/Ir^II^] = +1.68 V vs SCE)^6b^ and an improved solubility profile in our reaction solvent. With this photocatalyst and a slightly longer reaction time, we scaled the hydroxyarylation reaction to 2 mmol without an appreciable decrease in yield (86%, Figure 3A). With the access to millimole quantities of hydroxyaryl product **3**, we subjected **3** to enzyme-catalyzed kinetic resolution. Using commercially available *Pseudomonas stutzeri* lipase, enantioenriched benzylic alcohols could be accessed in just two steps from **1** and **2** (Figure 3A).^15^

**Figure 3:**
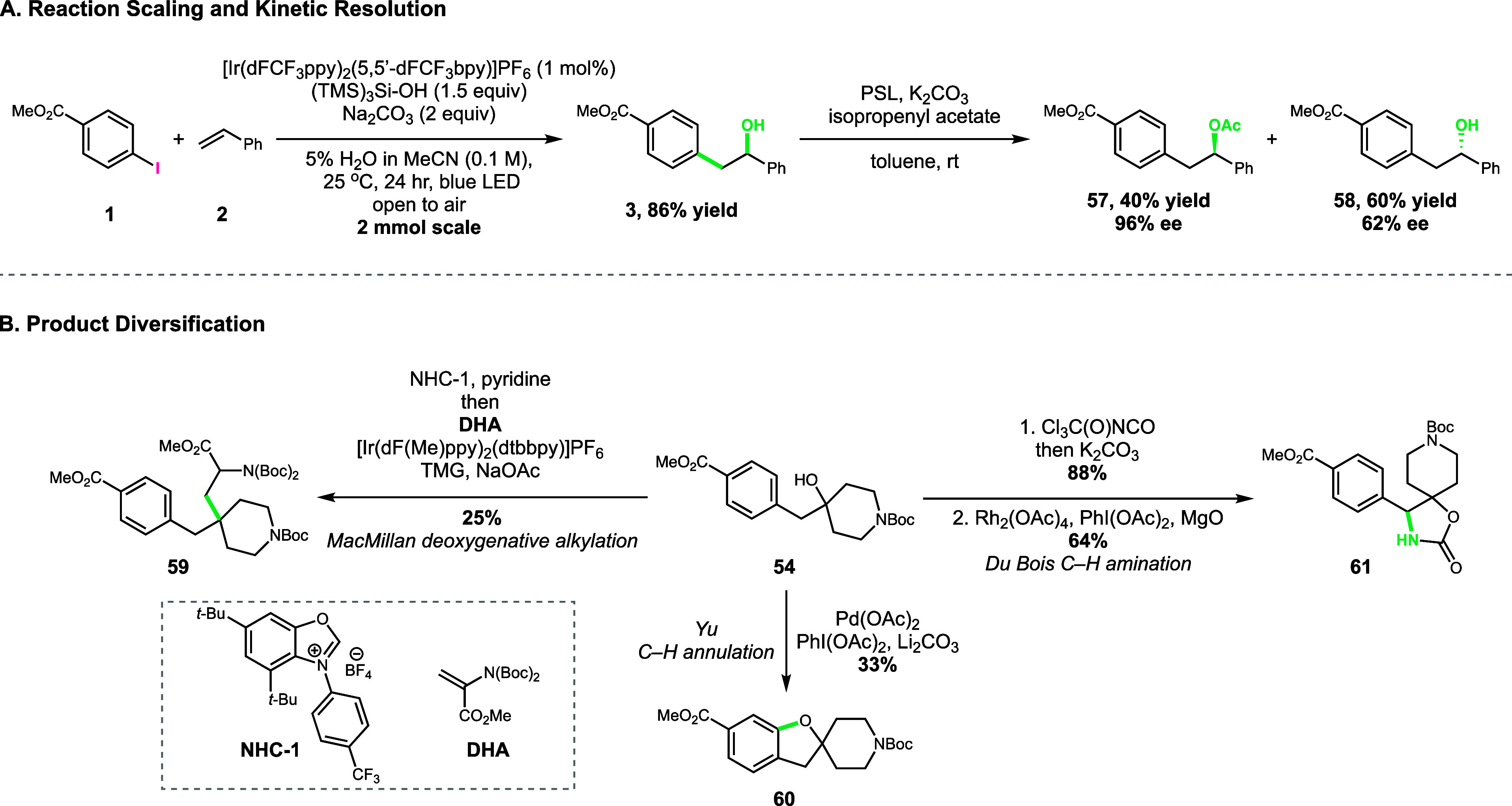
(A) Rapid access to enantioenriched 1,2-diarylalcohols on mmol scale. (B) Diversification of the aliphatic alcohol **54** hydroxyaryl product.

Additionally, we highlight the ability to diversify alcohol products into a range of medicinally relevant motifs. Using a deoxygenative alkylation protocol reported by MacMillan and co-workers, **54** could be transformed into quaternary carbon containing unnatural amino acid **59** (25% yield, Figure 3B).^16^ The free hydroxyl group of **54** could be utilized for a Yu C–H annulation to deliver spirocyclic benzofuran/piperidine heterocycle **60** in 33% yield.^17^ Finally, functionalization of the benzylic site through Du Bois C–H amination produced synthetically useful oxazolidinone **61** (Figure 3B).^18^ In all cases, the reported yields represent reactions run under the originally reported conditions. No attempts to optimize for this particular substrate were made, highlighting the ability to diversify without the need for significant extra experiments.

In summary, we present the first example of a hydroxyarylation of alkenes using aryl halides and O_2_. Our methodology has significant potential for industrial impact given it utilizes abundant feedstock chemicals, displays a diverse substrate scope, and is operationally simple to set up. In addition to our methodology, providing rapid access to biologically relevant hydroxyaryl scaffolds, these products can undergo a variety of transformations to build molecular complexity and introduce new motifs. Investigations to develop new reactions using our O_2_-tolerant oxidative aryl radical generation conditions are ongoing.

## Supporting Information Available

Experimental procedures, characterization data, and spectra (PDF)
