# Supplementary material for: Photoredox-Driven Three-Component Coupling of Aryl Halides, Olefins, and O2
Source: ACS Catal. 2024 Feb 4;14(4):2582–7. doi: 10.1021/acscatal.3c05988 (PMC10877571; doi:10.1021/acscatal.3c05988)
Supplement: Supplementary file 1 — cs3c05988_si_001.pdf [file cs3c05988_si_001.pdf]

Supporting Information

Photoredox-Driven Three-Component Coupling of Aryl Halides, Olefins, and O_2

Mark C. Maust and Simon B. Blakey*

Corresponding author: sblakey@emory.edu

Department of Chemistry, Emory University, Atlanta, Georgia 30322, United States

I. General information
- a. **General Reagent Information (S2)**
- b. **General Analytical Information (S2)**

II. General Procedures
- a. **Optimization Procedure**
- b. **General Procedure A (S2)**
- c. **General Procedure B (S2)**
- d. **General Photoredox Setup (S3)**

III. Extended Optimization
- a. **Extended optimization experiments (S4)**
- b. **Failed substrates (S5)**

IV. Preparation of Starting Materials and Reagents
- a. **Preparation of Cl-4CzIPN (S6)**
- b. **Preparation of tris(trimethylsilyl)silanol (S6)**
- c. **Preparation of substrates (S7)**

V. Mechanistic Experiments
- a. **$^{18}O_2$ experiment (S8)**
- b. **$H_2{}^{18}O$ experiment (S9)**

VI. Preparation of Products from Substrate Table (S10-S25)

VII. Scaling Experiments
- a. **Optimization of reaction set-up on 0.5 mmol scale (S26)**
- b. **Optimization of reaction set up on 2 mmol scale (S27)**

VIII. Preparation of Products from Product Diversification Figure
- a. **Kinetic Resolution of 3 (S28)**
- b. **Deoxygenative Alkylation of 54 (S30)**
- c. **C–H annulation of 54 (S30)**
- d. **C–H amination of 54 (S31)**

IX. References (S32)

X. NMR Spectra(S34)

I. General Information

General Reagent Information:

Reagents were purchased from Sigma-Aldrich, Alfa Aesar, Acros Organics, Combi-Blocks, Oakwood Chemicals, Ambeed, and TCI America and used as received, unless stated otherwise. *Pseudomonas stutzeri* lipase (PSL) enzyme was provided by Meito Sangyo. All reactions were carried out under nitrogen atmosphere with anhydrous solvents in oven- or flame-dried glassware using standard Schlenk technique, unless otherwise stated. Alkenes that were packaged with radical inhibitors were passed through a pipette plug of aluminum oxide, activated, Brockman I immediately prior reaction set-up to remove inhibitor prior to adding to the reaction. Reactions requiring irradiation were done so with blue LEDs (Hydrofarm® PPB1002 PowerPAR LED Bulb-Blue 15W/E27 (available from Amazon). Optimization experiments performed at temperatures at or below 15 ºC were done so using a Thermo/Neslab CB80 Cryocool chamber containing an alumium block reaction tube holder and irriadiated by a CHANZON High Power 100W LED 440 nm chip powered by a Mean Well HLG-240H-36B LED driver. Optimization experiments with light intensity less than 100% were done so using a Kessil PR160L 456 nm for irradiation. Optimization experiments conducted at temperatures above 30 ºC were done so using a Acceled Photoreactor M2. Flash chromatography was carried out using Siliaflash® P60 silica gel obtained from Silicycle. Thin-layer chromatography (TLC) was performed on 250 μm SiliCycle silica gel F-254 plates. Visualization of the developed chromatogram was performed by fluorescence quenching or staining using $KMnO_4$ or Seebach's stain. Anhydrous dichloromethane (DCM), diethyl ether (Et_2O), tetrahydrofuran (THF), and toluene were obtained by passage through activated alumina using a Glass Contours solvent purification system.

General Analytical Information:

Unless otherwise noted, all yields refer to chromatographically and spectroscopically (^1H NMR) homogenous materials. New compounds were characterized by NMR and HRMS. ^1H and ^{13}C NMR spectra were obtained from the Emory University NMR facility and recorded on a Bruker Avance III HD 600 equipped with cryo-probe (600 MHz), Bruker 400 (400 MHz), INOVA 600 (600 MHz), INOVA 500 (500 MHz), INOVA 400 (400 MHz), or VNMR 400 (400 MHz), and are internally referenced to residual proton solvent signals. Data for ^1H NMR are reported as follows: chemical shift (ppm), multiplicity (s = singlet, d = doublet, t = triplet, q = quartet, m = multiplet, dd = doublet of doublets, dt = doublet of triplets, ddd= doublet of doublet of doublets, dtd= doublet of triplet of doublets, b = broad, etc.), coupling constant (Hz), integration, and assignment, when applicable. Data for decoupled ^{13}C NMR are reported in terms of chemical shift and multiplicity when applicable. High Resolution mass spectra were obtained from the Emory University Mass Spectral facility using a Thermo Scientific Extractive Plus with an orbitrap mass analyzer. The emission spectrum for the Hydrofarm® PPB1002 PowerPAR LED Bulb-Blue 15W/E27 light source was obtained using an Ocean Optics USB4000 spectrophotometer.

II. General Procedures

Optimization General Procedure

An 8 mL screw-top reaction tube equipped with a stir bar was charged with Cl-4CzIPN (5 mol%), base (2 equiv), aryl iodide (1.0 equiv). Solvent was added to the reaction tube. Alkene (3.0 equiv) and $(TMS)_3SiOH$ (1.5 equiv) were added via syringe. The tube was capped with a PTFE/silicon septum and pierced with an 18 G needle. The resulting mixture was stirred open to air at room temperature at 1000 RPM for 16 h under irradiation by an overhead blue LED lamp located ~6 inches above the reaction. The crude reaction mixture was diluted with EtOAc (5 mL), passed through a pad of silica, and the filtrate was concentrated *in vacuo*. Dibromomethane (0.1 mmol, 7 μL) as internal standard was added and the crude sample was analyzed by ^1H NMR (d = 5s) to obtain reaction yield.

General Procedure A

A 20 mL screw-top reaction tube equipped with a stir bar was charged with Cl-4CzIPN (5 mol%), Na_2CO_3 (2 equiv), aryl iodide (1.0 equiv). Benchtop MeCN and deionized H_2O (19:1 MeCN:H_2O, 0.1 M) was added to the reaction tube.

Alkene (3.0 equiv) and $(TMS)_3SiOH$ (1.5 equiv) were added via syringe. The resulting mixture was stirred open to air at 1400 RPM for 16 h under irradiation by blue LEDs at room temperature. The crude reaction mixture was diluted with 20 mL of EtOAc and added to a separatory funnel containing 1 M K_2CO_3 (aq) (10 mL). The layers were separated, and the organic layer was washed twice more with 1 M K_2CO_3 (10 mL) followed by brine (20 mL). The organic layer was dried over sodium sulfate and concentrated *in vacuo*. The crude sample was purified by silica chromatography using the indicated solvent mixture as the eluent to afford the title compound. Occasionally, nonpolar compounds coelute with silyl related byproducts. In these cases, the compound was dissolved in a minimal amount of DCM and loaded onto a pipette alumina plug (ca. 3 inches of alumina). This was washed with 10 mL of 50% hexanes/DCM to elute the silyl byproduct followed by 10 mL of EtOAc to elute the desired product.

General Procedure B

A 20 mL screw-top reaction tube equipped with a stir bar was charged with Cl-4CzIPN (5 mol%), Na_2CO_3 (2 equiv), aryl iodide (1.0 equiv). Benchtop MeCN and distilled H_2O (19:1 MeCN:H_2O, 0.1 M) was added to the reaction tube. Alkene (3.0 equiv) and $(TMS)_3SiOH$ (1.5 equiv) were added via syringe. The resulting mixture was stirred open to air at 1400 RPM for 48 h under irradiation by blue LEDs at room temperature. The crude reaction mixture was diluted with EtOAc (10 mL), pushed through a pad of celite, and concentrated *in vacuo*. The crude sample was purified by silica chromatography using the indicated solvent mixture as the eluent to afford the title compound.

General Photoredox Set-up:

The reactions were conducted in a solvent cabinet fitted with a large fan, cutout holes to maintain reactions at room temperature, and reflective tape (photos 1-4). The reactions were placed in an appropriately sized 3D printed carousel, allowing multiple reactions to be run simultaneously. A 15W blue LED array lamp was placed 6 inches above the reactions.

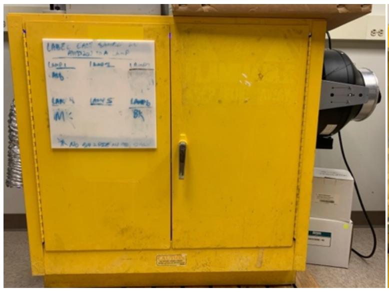
Photo 1

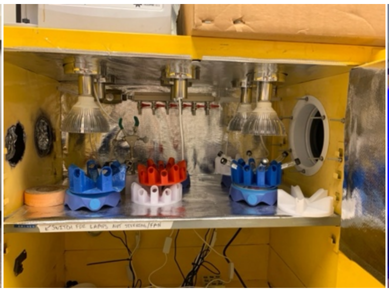
Photo 2

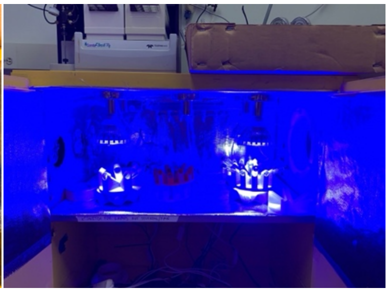
Photo 3

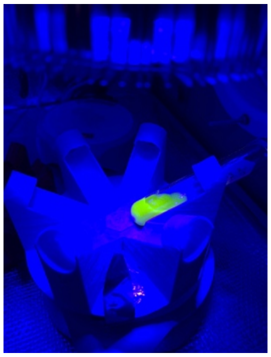
Photo 4

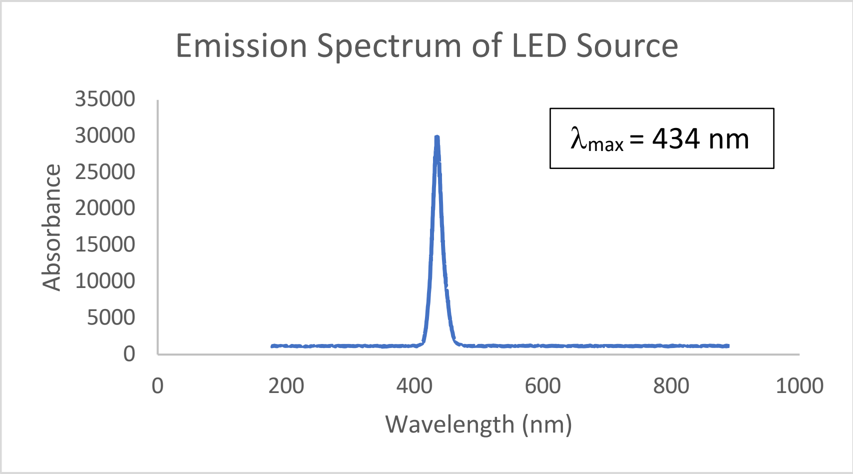

III. Extended Optimization

Table S1. Extended Optimization experiments

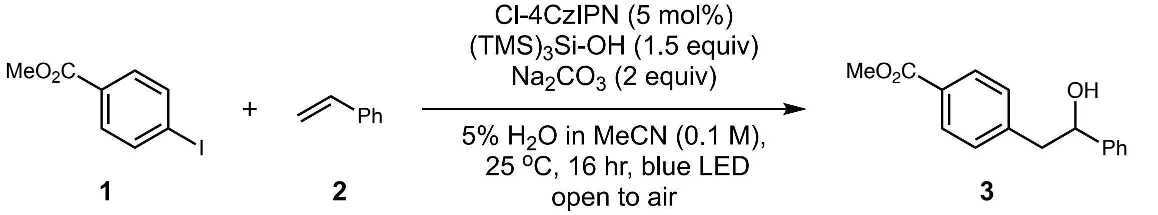

Entry	1	Deviation	3 Yield
1	92%	$Ir(ppy)_3$ as photocatalyst	0%
2	93%	$[Irdtbbpy(ppy)_2]PF_6$ as photocatalyst	0%
3	90%	$(Ir[dFCF_3ppy)]_2(dtbbpy))PF_6$ as photocatalyst	0%
4	14%	-15 ºC	51%
5	6%	0 ºC	60%
6	0%	15 ºC	66%
7	0%	30 ºC	79%
8	0%	40 ºC	69%
9	0%	50 ºC	55%
10	14%	0.4 M	76%
11	11%	0.2 M	84%
12	0%	0.05 M	76%
13	13%	0.025 M	58%
14	0%	75% light intensity	79%
15	0%	50% light intensity	80%
16	23%	25% light intensity	66%
17	23%	25% light intensity	66%
18	0%	2 eq $(TMS)_3SiOH$	90%
19	0%	3 eq $(TMS)_3SiOH$	89%
20	0%	4 eq $(TMS)_3SiOH$	85%

^{1}H NMR yields with 0.1 mmol dibromomethane as internal standard

Low-yielding substrates:

Aryl Halides:

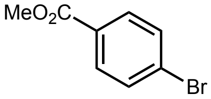

13%

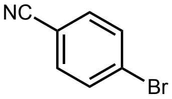

17%

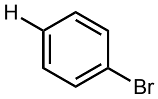

6%

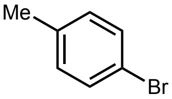

5%

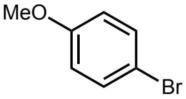

0%

Alkenes:

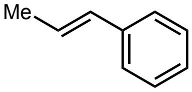

<10%

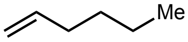

14%

IV. Preparation of Starting Materials and Reagents

Preparation of Cl-4CzIPN:

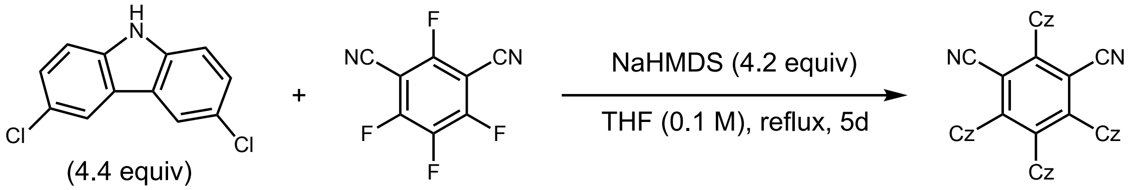

2,4,5,6-Tetrakis(3.6-dichloro-9H-carbazol-9-yl)isopthalonitrile (Cl-4CzIPN):
The following procedure was modified from a previous reported procedure by Molander et al.[1] An oven-dried 100 mL round-bottom flask was equipped with a stir bar and charged with 3,6-dichloro-9H-carbazole (4.155 g, 17.6 mmol, 4.4 equiv). The flask was sealed with a septum and the atmosphere was exchanged by applying vacuum and backfilling with nitrogen (this process was conducted a total of three times). Under nitrogen atmosphere, THF (40 mL) was added and the flask was cooled to 0 ºC. Once cooled, a solution of NaHMDS in THF (16.8 mL, 16.8 mmol, 4.2 equiv, 1 M in THF) was added slowly. After complete addition, the mixture was stirred for 10 minutes at 0 ºC followed by 30 minutes at room temperature. After this time, 2,4,5,6-tetrafluoroisopthalonitrile was added in one portion. The flask was then heated to reflux for 5 days.

After 5 days, the reaction was cooled to room temperature and filtered through a large coarse fritted funnel. The solid was washed with Et_2O (~500 mL) and the filtrate was discarded. The solid was then washed with $CHCl_3$ (~500 mL) to elute the desired product. The filtrate was transferred to a round-bottom flask and the solvent was removed *in vacuo*. The resulting solid was washed with a 75:25 mixture of pentanes (2 x 100 mL), followed by pentane (100 mL). The resulting bright yellow solid was dried under vacuum to give 2,4,5,6-Tetrakis(3.6-dichloro-9H-carbazol-9-yl)isopthalonitrile (2.334 g, 55%). The physical properties and spectral data were consistent with those reported in the literature.[1]

Preparation of $(TMS)_3SiOH$

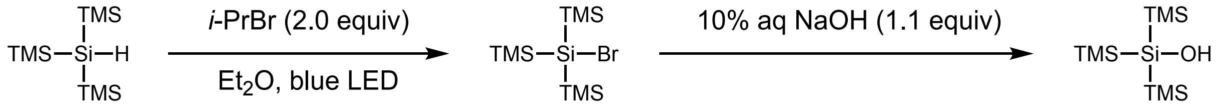

Tris(trimethylsilyl)silanol ($(TMS)_3SiOH$): The following procedure was modified from a previous reported procedure by MacMillan et al.[2] Under air, a 20 mL screw top reaction tube was equipped with a magnetic stir bar and charged with tri(trimethylsilyl)silane (3.86 mL, 12.5 mmol, 1.0 equiv), 2-bromopropane (2.35 mL, 25.0 mmol, 2.0 equiv), and Et_2O (4 mL). The reaction tube was capped with a PTFE/silicon septum and irradiated with blue LEDs for 12 hours. After irradiation, the septum was carefully pierced with a needle to allow the slow evolution of gas. Once bubbling subsided, the solution was transferred to a round-bottom flask containing 10% aq NaOH solution (11 mL, 1.1 eq) and stirred for 24 hours under air at room temperature. After this time, the solution was transferred to a separatory funnel, Et_2O (10 mL) was added, and the organic layer was separated. The organic layer was dried with Na_2SO_4, followed by concentration *in vacuo*. The crude silanol sample was then placed on high vacuum for 8 hours to remove solvent and residual silane impurities to yield pure silanol as a clear oil. The physical properties and spectral data were consistent with those reported in literature.[2]

Preparation of substrates:

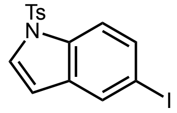

6-iodo-1-tosyl-1*H*-indole (S1): An oven-dried 100 mL round-bottom flask was equipped with a stir bar and charged with 6-iodo-1*H*-indole (1.21 g, 5 mmol, 1.0 equiv) and equipped with a nitrogen balloon. To the flask was added 15 mL of DMF and the mixture was cooled to 0 ºC. NaH 60% dispersion in mineral oil (0.240 g, 1 mmol, 1.2 equiv) was added to the mixture. The reaction was warmed to rt and stirred for 30 minutes. After that time *p*-toluenesulfonyl chloride (1.90 g, 10 mmol, 2.0 equiv) was added to the mixture and stirred for 16 hours. The reaction was quenched with water (10 mL) and extracted with EtOAc (30 mL). The organic layer was washed with 1 M LiCl (3 x 10 mL), followed by brine (3 x 10 mL), dried over Na_2SO_4, filtered, and concentrated *in vacuo*. The crude material was recrystallized from DCM/Hexanes to give the title compound as a white solid (1.240 g, 62%). The physical properties and spectral data were consistent with those reported in literature.[3]

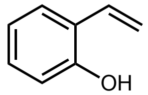

2-vinylphenol (S2): A mixture of methyl triphenylphosphonium bromide (4.29 g, 12.3 mmol, 1.5 equiv) in THF (16 mL) was treated with potassium *t*-butoxide (2.30 g, 20.5 mmol, 2.5 equiv). After stirring for 10 minutes at room temperature, a solution of 4-hydroxybenzaldehyde (1.00 g, 8.19 mmol, 1.0 equiv) in THF (2 mL) was added dropwise to the suspension. The resulting mixture was stirred at room temperature for one hour. After this time, the reaction was quenched with sat. NH_4Cl (10 mL) and concentrated *in vacuo* to remove THF. The concentrated mixture was extracted with DCM (40 mL). The organic layer was washed 3 x with brine (40 mL) and dried over Na_2SO_4, filtered, and concentrated *in vacuo*. The reaction was purified by silica gel chromatography (5% EtOAc/Hex) to give the title product as a colorless oil. The physical properties and spectra data are consisted with those reported in literature.[4]

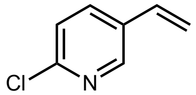

2-chloro-5-vinylpyridine (S3): A mixture of methyl triphenylphosphonium bromide (30.3 g, 85 mmol, 1.2 equiv) in THF (150 mL) was cooled to 0 ºC and treated with potassium *t*-butoxide (9.5 g, 85 mmol, 1.2 equiv) dissolved in 30 mL THF dropwise over 5 minutes. After stirring for 30 minutes at room temperature, 6-chloronicotinaldheyde (10 g, 71 mmol, 1.0 equiv) was added dropwise to the suspension. The resulting mixture was stirred at room temperature for two hours. After this time, the reaction was quenched with sat. NH_4Cl (160 mL) and concentrated *in vacuo* to remove THF. The concentrated mixture was diluted with EtOAc (100 mL), washed with brine (100 mL), and the organic layer was stirred over activate charcoal to remove colored impurities. The mixture was then filtered through celite and the filtrate was dried over Na_2SO_4 and concentrated *in vacuo.* The resulting semis-solid was stirred overnight in pentanes (150 mL) to precipitate the phosphine byproduct. After this time, the solution was filtered through a silica plug (~50 g) and the solid was washed with 2:1 Et_2O:pentane until olefin spots (as confirmed by TLC) no longer eluted from the plug. The combined filtrates were concentrated *in vacuo* and purified by flash chromatography (0-5% Et_2O/Hex) to yield the title compound as a clear colorless oil. The physical properties and spectra data are consisted with those reported in the literature.[5]

V. Mechanistic Experiments

$^{18}O_2$ labeling experiment:

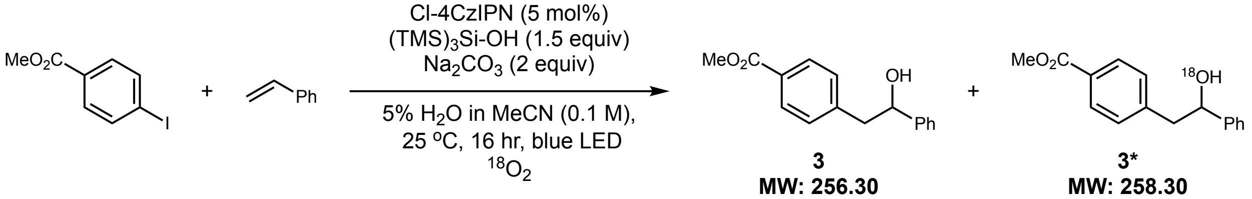

A 20 mL screw-top reaction tube was equipped with a stir bar and charged with methyl 4-iodobenzoate (0.5 mmol, 131 mg, 1.0 equiv), Na_2CO_3 (1 mmol, 106 mg, 2.0 equiv), Cl-4CzIPN (0.025 mmol, 26 mg, 5 mol%), $(TMS)_3SiOH$ (0.75 mmol, 0.230 mL, 1.5 equiv), styrene (1.5 mmol, 0.172 mL, 3.0 equiv), and 19:1 MeCN:H_2O (5 mL) and the tube was sealed with a PTFE/silicon septum. The reaction was degassed by placing under high vacuum while stirring until bubbling was observed then holding at static vacuum for 3 minutes (this process was repeated a total of 5 times). After degassing the reaction, $^{18}O_2$ was transferred to the reaction head space. The reaction was sealed with Teflon tape and was stirred at 1400 RPM for 16 hour under irradiation from blue LEDs. The crude reaction mixture was diluted with 20 mL of EtOAc and added to a separatory funnel containing 10 mL of 1 M K_2CO_3 (aq). The layers were separated, and the organic layer was washed twice more with 1 M K_2CO_3 followed by brine. The organic layer was dried over sodium sulfate and concentrated *in vacuo*. The crude sample was purified by silica chromatography (0-20% EtOAc/Hex) to afford a mixture of **3** and **3*** in 70% yield. The percent of $^{18}O_2$ incorporation was determined from the mass spectroscopy data shown below. HRMS detected [M+H] peaks for both **3** and **3***

HRMS (ESI pos.) m/z: [M+H] calcd. for $C_{16}H_{17}O_3$, 257.11722; found 257.1171.

HRMS (ESI pos.) m/z: [M+H] calcd. for $C_{16}H_{17}O_2{}^{18}O$, 259.12147; found 259.12142.

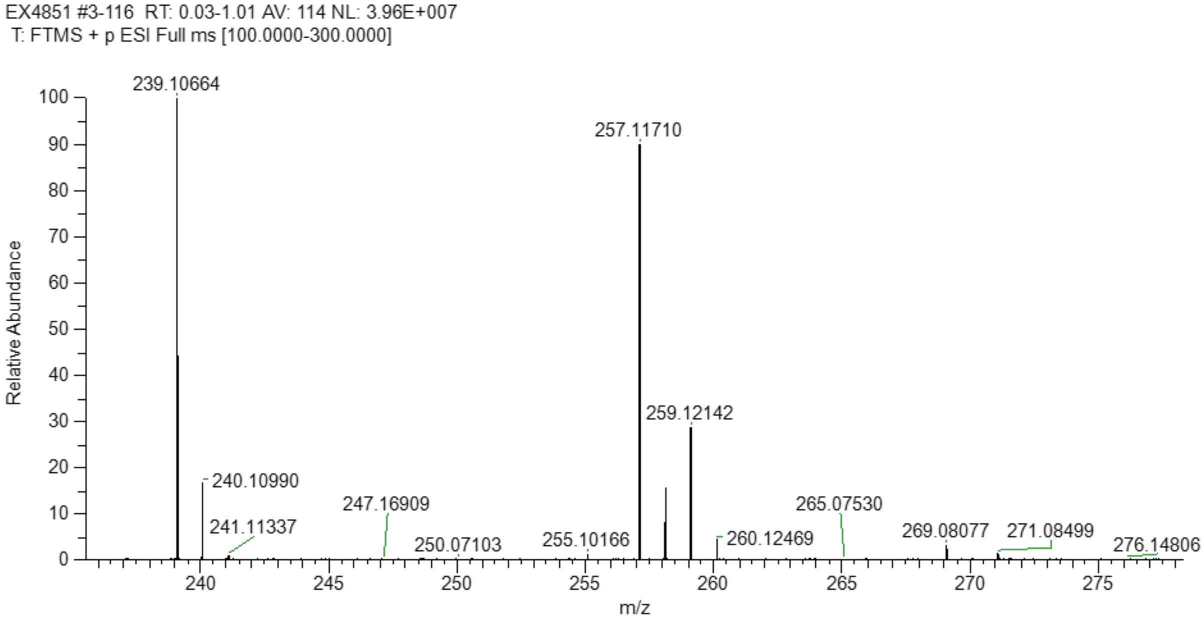

$H_2{}^{18}O$ labeling experiment:

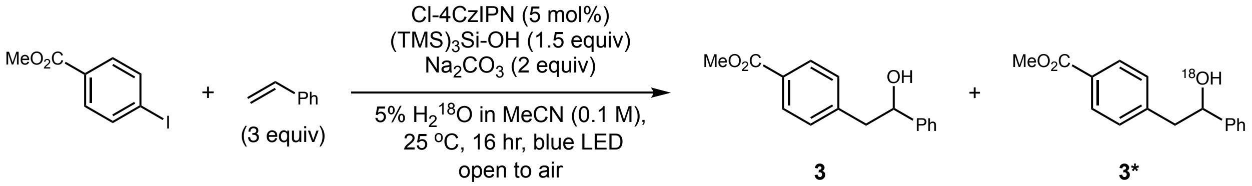

3
MW: 256.30

3*
MW: 258.30

An oven-dried 20 mL screw-top reaction tube equipped with a stir bar was charged with Cl-4CzIPN (5 mol%), Na_2CO_3 (2 equiv), aryl iodide (1.0 equiv). Anhydrous MeCN and $H_2{}^{18}O$ (19:1 MeCN:H_2O, 0.1 M) was added to the reaction tube. Alkene (3.0 equiv) and $(TMS)_3SiOH$ (1.5 equiv) were added via syringe. The resulting mixture was stirred open to air at 1400 RPM for 16 h under irradiation by blue LEDs at room temperature. The crude reaction mixture was diluted with EtOAc (20 mL) and added to a separatory funnel containing 1 M K_2CO_3 (aq) (10 mL). The layers were separated, and the organic layer was washed twice more with 1 M K_2CO_3 (10 mL) followed by brine (20 mL). The organic layer was dried over sodium sulfate and concentrated *in vacuo*. The reaction was purified by silica chromatography (0-20% EtOAc/Hex) to afford **3** as a pale yellow solid (98.2 mg, 76%). HRMS only detected a peak for **3**, thus no $^{18}O_2$ was incorporated.

HRMS (APCI pos.) m/z: [M+H] calcd. for $C_{16}H_{17}O_3$, 257.11722; found 257.1171.

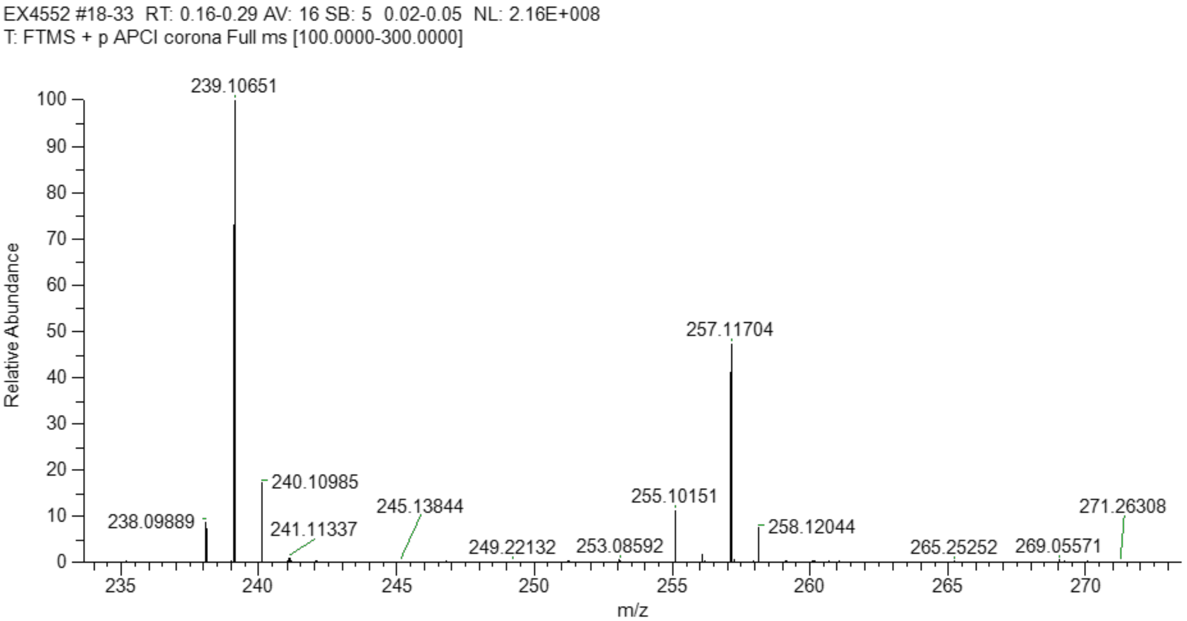

VI. Preparation of Products from Substrate Table

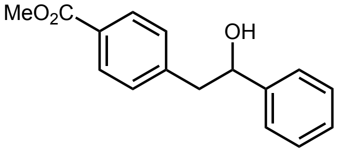

Methyl 4-(2-hydroxy-2-phenylethyl)benzoate (3): Prepared according to general procedure A using methyl 4-iodobenzoate (0.5 mmol, 131 mg, 1.0 equiv), Na_2CO_3 (1 mmol, 106 mg, 2.0 equiv), Cl-4CzIPN (0.025 mmol, 26 mg, 5 mol%), $(TMS)_3SiOH$ (0.75 mmol, 0.230 mL, 1.5 equiv), and styrene (1.5 mmol, 0.172 mL, 3.0 equiv) in 19:1 MeCN:H_2O (5 mL). The reaction was purified by silica chromatography (0-20% EtOAc/Hex) to afford the title compound as a pale yellow solid (115 mg, 89%). The physical properties and spectral data were consistent with those reported in literature.[6]

^{1}H NMR (600 MHz, $CDCl_3$) δ 7.95 (d, J = 8.0 Hz, 2H), 7.37 – 7.27 (m, 5H), 7.24 (d, J = 8.0 Hz, 2H), 4.93 (t, J = 6.7 Hz, 1H), 3.90 (s, 3H), 3.13 – 3.02 (m, 2H), 1.94 (s, 1H).

^{13}C NMR (101 MHz, $CDCl_3$) δ 167.20, 143.66, 143.63, 129.83, 129.73, 128.66, 128.62, 127.99, 126.01, 75.31, 52.19, 46.01.

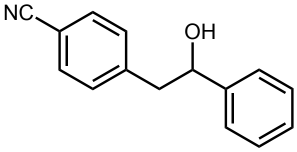

4-(2-hydroxy-2-phenylethyl)benzonitrile (5): Prepared according to general procedure A using methyl 4-iodobenzonitrile (0.5 mmol, 115 mg, 1.0 equiv), Na_2CO_3 (1 mmol, 106 mg, 2.0 equiv), Cl-4CzIPN (0.025 mmol, 26 mg, 5 mol%), $(TMS)_3SiOH$ (0.75 mmol, 0.230 mL, 1.5 equiv), and styrene (1.5 mmol, 0.172 mL, 3.0 equiv) in 19:1 MeCN:H_2O (5 mL). The reaction was purified by silica chromatography (0-30% EtOAc/Hex) to afford the title compound as a pale yellow solid (96.2 mg, 86%). The physical properties and spectral data were consistent with those reported in literature.[7]

^{1}H NMR (600 MHz, $CDCl_3$) δ 7.54 (d, J = 7.9 Hz, 2H), 7.42 – 7.27 (m, 5H), 7.27 – 7.23 (d, J = 7.9 Hz, 2H), 4.93-4.88 (m, 1H), 3.10 (dd, J = 13.6, 8.0 Hz, 1H), 3.05 (dd, J = 13.6, 5.1 Hz, 1H), 2.05 (d, J = 2.9 Hz, 1H).

^{13}C NMR (101 MHz, $CDCl_3$) δ 143.94, 143.38, 132.15, 130.51, 128.71, 128.15, 125.94, 119.12, 110.44, 75.07, 45.84.

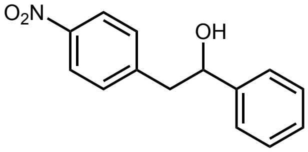

2-(4-nitrophenyl)-1-phenylethan-1-ol (6): Prepared according to general procedure A using 1-iodo-4-nitrobenzene (0.5 mmol, 125 mg, 1.0 equiv), Na_2CO_3 (1 mmol, 106 mg, 2.0 equiv), Cl-4CzIPN (0.025 mmol, 26 mg, 5 mol%), $(TMS)_3SiOH$ (0.75 mmol, 0.230 mL, 1.5 equiv), and styrene (1.5 mmol, 0.172 mL, 3.0 equiv) in 19:1 MeCN:H_2O (5 mL). The reaction was purified by silica chromatography (0-20% EtOAc/Hex) to afford the title compound as a yellow solid (75.6 mg, 62%). The physical properties and spectral data were consistent with those reported in literature.[7]

^{1}H NMR (600 MHz, $cdcl_3$) δ 8.12 (d, J = 8.8 Hz, 2H), 7.39 – 7.32 (m, 2H), 7.35 – 7.25 (m, 5H), 4.97 – 4.92 (m, 1H), 3.16 (dd, J = 13.7, 7.9 Hz, 1H), 3.10 (dd, J = 13.6, 5.2 Hz, 1H), 1.96 (d, J = 3.0 Hz, 1H).

^{13}C NMR (101 MHz, $CDCl_3$) δ 146.91, 146.07, 143.31, 130.58, 128.79, 128.26, 125.96, 123.60, 75.11, 45.56.

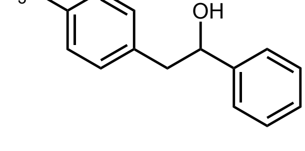

1-phenyl-2-(4-(trifluoromethyl)phenyl)ethan-1-ol (7): Prepared according to general procedure A using 1-iodo-4-(trifluoromethyl)benzene (0.5 mmol, 136 mg, 1.0 equiv), Na_2CO_3 (1 mmol, 106 mg, 2.0 equiv), Cl-4CzIPN (0.025 mmol, 26 mg, 5 mol%), $(TMS)_3SiOH$ (0.75 mmol, 0.230 mL, 1.5 equiv), and styrene (1.5 mmol, 0.172 mL, 3.0 equiv) in 19:1 MeCN:H_2O (5 mL). The reaction was purified by silica chromatography (0-10% EtOAc/Hex), followed by an alumina plug as described in general procedure A, to afford the title compound as a yellow solid (131 mg, 98%). The physical properties and spectral data were consistent with those reported in literature.[7]
^{1}H NMR (500 MHz, $CDCl_3$) δ 7.54 (d, *J* = 7.9 Hz, 2H), 7.40 7.24 (m, 7H), 4.93 (t, *J* = 6.6 Hz, 1H), 3.14 – 3.02 (m, 2H), 1.91 (s, 1H).
^{13}C NMR (101 MHz, $CDCl_3$) δ 143.61, 142.40 (d, *J* = 1.4 Hz), 130.01, 128.99 (q, *J* = 32.3 Hz) 128.71, 128.08, 126.00, 125.41(q, *J* = 3.7 Hz), 124.91 (q, *J* = 271.9), 75.31, 45.73.
^{19}F NMR (376 MHz, $CDCl_3$) δ -62.39.

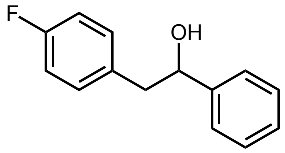

2-(4-fluorophenyl)-1-phenylethan-1-ol (8): Prepared according to general procedure A using 1-fluoro-4-iodobenzene (0.5 mmol, 111 mg, 1.0 equiv), Na_2CO_3 (1 mmol, 106 mg, 2.0 equiv), Cl-4CzIPN (0.025 mmol, 26 mg, 5 mol%), $(TMS)_3SiOH$ (0.75 mmol, 0.230 mL, 1.5 equiv), and styrene (1.5 mmol, 0.172 mL, 3.0 equiv) in 19:1 MeCN:H_2O (5 mL). The reaction was purified by silica chromatography (0-20% EtOAc/Hex) to afford the title compound as a yellow solid (83.7 mg, 77%). The physical properties and spectral data were consistent with those reported in literature.[7]
^{1}H NMR (500 MHz, $CDCl_3$) δ 7.38 – 7.27 (m, 5H), 7.16 – 7.10 (m, 2H), 7.01 – 6.94 (m, 2H), 4.87 (t, *J* = 6.6 Hz, 1H), 3.00 (d, *J* = 6.6 Hz, 2H), 1.99 (broad s, 1H).
^{13}C NMR (101 MHz, $CDCl_3$) δ 161.87 (d, *J* = 244.6 Hz), 143.74, 133.77 (d, *J* = 3.6 Hz) 131.08 (d, *J* = 8.0 Hz), 128.58, 127.86, 126.02, 115.32(d, *J* = 21.0 Hz), 75.50, 45.17.
^{19}F NMR (376 MHz, $CDCl_3$) δ -116.59.

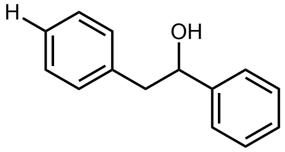

1,2-diphenylethan-1-ol (9): Prepared according to general procedure A using iodobenzene (0.5 mmol, 102 mg, 1.0 equiv), Na_2CO_3 (1 mmol, 106 mg, 2.0 equiv), Cl-4CzIPN (0.025 mmol, 26 mg, 5 mol%), $(TMS)_3SiOH$ (0.75 mmol, 0.230 mL, 1.5 equiv), and styrene (1.5 mmol, 0.172 mL, 3.0 equiv) in 19:1 MeCN:H_2O (5 mL). The reaction was purified by silica chromatography (0-10% EtOAc/Hex), followed by an alumina plug as described in general procedure A, to afford the title compound as a white solid (90.9 mg, 92%). The physical properties and spectral data were consistent with those reported in literature.[8]
^{1}H NMR (600 MHz, $CDCl_3$) δ 7.40 – 7.32 (m, 4H), 7.32 – 7.27 (m, 3H), 7.27-7.23 (m, 1H), 7.23-7.19 (m, 2H) 4.90 (m, 1H), 3.05 (dd, *J* = 13.7, 4.8 Hz, 1H), 3.00 (dd, *J* = 13.7, 8.6 Hz, 1H), 1.99 (broad s, 1H).
^{13}C NMR (101 MHz, $CDCl_3$) δ 143.94, 138.17, 129.65, 128.65, 128.55, 127.76, 126.76, 126.03, 75.48, 46.23.

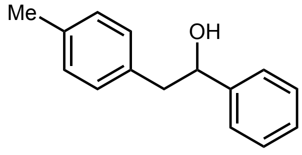

1-phenyl-2-(*p*-tolyl)ethan-1-ol (10): Prepared according to general procedure A using 1-iodo-4-methylbenzene (0.5 mmol, 109 mg, 1.0 equiv), Na_2CO_3 (1 mmol, 106 mg, 2.0 equiv), Cl-4CzIPN (0.025 mmol, 26 mg, 5 mol%), $(TMS)_3SiOH$ (0.75 mmol, 0.230 mL, 1.5 equiv), and styrene (1.5 mmol, 0.172 mL, 3.0 equiv) in 19:1 MeCN:H_2O (5 mL). The reaction was purified by silica chromatography (0-10% Acetone/Hex), followed by an alumina plug as described in general procedure A, to afford the title compound as a white solid (87.4 mg, 81%). The physical properties and spectral data were consistent with those reported in literature.[7]

^{1}H NMR (600 MHz, CDCl$_3$) δ 7.41 – 7.33 (m, 4H), 7.33 – 7.25 (m, 1H), 7.16 – 7.08 (m, 4H), 4.87 (dd, *J* = 8.8, 4.6 Hz, 1H), 3.02 (dd, *J* = 13.7, 4.6 Hz, 1H), 2.94 (dd, *J* = 13.8, 8.9 Hz, 1H), 2.33 (s, 3H).
^{13}C NMR (151 MHz, CDCl$_3$) δ 144.03, 136.34, 135.02, 129.51, 129.39, 128.55, 127.71, 126.04, 75.51, 45.85, 21.20.

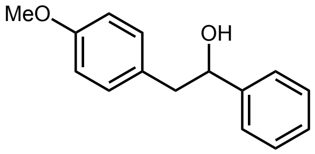

2-(4-methoxyphenyl)-1-phenylethan-1-ol (11): Prepared according to general procedure A using 1-iodo-4-methoxybenzene (0.5 mmol, 117 mg, 1.0 equiv), Na_2CO_3 (1 mmol, 106 mg, 2.0 equiv), Cl-4CzIPN (0.025 mmol, 26 mg, 5 mol%), $(TMS)_3SiOH$ (0.75 mmol, 0.230 mL, 1.5 equiv), and styrene (1.5 mmol, 0.172 mL, 3.0 equiv) in 19:1 MeCN:H_2O (5 mL). The reaction was purified by silica chromatography (0-20% EtOAc/Hex) to afford the title compound as a yellow solid (89.1 mg, 78%). The physical properties and spectral data were consistent with those reported in literature.[9]
^{1}H NMR (500 MHz, CDCl$_3$) δ 7.37-7.33 (m, 4H), 7.32 – 7.27 (m, 1H), 7.11 (d, *J* = 8.7 Hz, 2H), 6.85 (d, *J* = 8.7 Hz, 2H), 4.86 (dd, *J* = 8.5, 4.8 Hz, 1H), 3.80 (s, 3H), 3.00 (dd, *J* = 13.8, 4.8 Hz, 1H), 2.93 (dd, *J* = 13.7, 8.5 Hz, 1H).
^{13}C NMR (101 MHz, CDCl$_3$) δ 158.50, 143.97, 130.61, 130.08, 128.52, 127.69, 126.04, 114.06, 75.55, 55.38, 45.31.

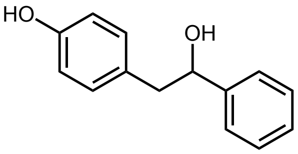

4-(2-hydroxy-2-phenylethyl)phenol (12): Prepared according to general procedure B using 4-iodophenol (0.5 mmol, 110 mg, 1.0 equiv), Na_2CO_3 (1 mmol, 106 mg, 2.0 equiv), Cl-4CzIPN (0.025 mmol, 26 mg, 5 mol%), $(TMS)_3SiOH$ (0.75 mmol, 0.230 mL, 1.5 equiv), and styrene (1.5 mmol, 0.172 mL, 3.0 equiv) in 19:1 MeCN:H_2O (5 mL). The reaction was purified by silica chromatography (10-30% EtOAc/Hex) to afford the title compound as a white solid (55.0 mg, 51%).
^{1}H NMR (600 MHz, CDCl$_3$) δ 7.34 (m, 4H), 7.30 – 7.23 (m, 1H), 7.05 (d, *J* = 8.3 Hz, 2H), 6.76 (d, *J* = 8.3 Hz, 2H), 4.85 (dd, *J* = 8.5, 4.8 Hz, 1H), 4.78 (s, 1H), 2.98 (dd, *J* = 13.8, 4.8 Hz, 1H), 2.92 (dd, *J* = 13.8, 8.5 Hz, 1H), 1.96 (s, 1H).
^{13}C NMR (151 MHz, CDCl$_3$) δ 154.44, 143.90, 130.84, 130.22, 128.55, 127.75, 126.06, 115.50, 75.60, 45.29.
HRMS (APCI neg.) m/z: [M-H] calcd. for $C_{14}H_{13}O_2$, 213.0921; found 213.0927.

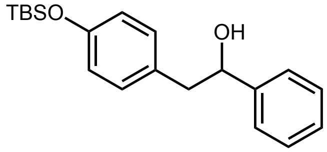

2-(4-((*tert*-butyldimethylsilyl)oxy)phenyl)-1-phenylethan-1-ol (13): Prepared according to general procedure A using *tert*-butyl(4-iodophenoxy)dimethylsilane (0.5 mmol, 167 mg, 1.0 equiv), Na_2CO_3 (1 mmol, 106 mg, 2.0 equiv), Cl-4CzIPN (0.025 mmol, 26 mg, 5 mol%), $(TMS)_3SiOH$ (0.75 mmol, 0.230 mL, 1.5 equiv), and styrene (1.5 mmol, 0.172 mL, 3.0 equiv) in 19:1 MeCN:H_2O (5 mL). The reaction was purified by silica chromatography (0-10% EtOAc/Hex), followed by an alumina plug as described in general procedure A, to afford the title compound as a yellow oil (136 mg, 87%).
^{1}H NMR (600 MHz, CDCl$_3$) δ 7.35 – 7.32 (m, 4H), 7.30 – 7.26 (m, 1H), 7.03 (d, *J* = 8.5 Hz, 2H), 6.77 (d, *J* = 8.5 Hz, 2H), 4.84 (dd, *J* = 8.5, 4.9 Hz, 1H), 2.98 (dd, *J* = 13.8, 4.9 Hz, 1H), 2.92 (dd, *J* = 13.8, 8.5 Hz, 1H), 0.98 (s, 9H), 0.19 (s, 6H).
^{13}C NMR (101 MHz, CDCl$_3$) δ 154.54, 143.93, 130.64, 130.59, 128.49, 127.67, 126.05, 120.25, 75.53, 45.48, 25.84, 18.35, -4.28.
HRMS (APCI pos.) m/z: [M+H] calcd. for $C_{20}H_{29}O_2Si$, 329.19313; found 329.1927.

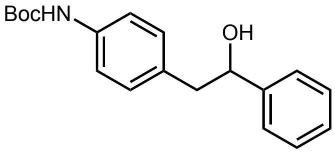

***tert*-butyl (4-(2-hydroxy-2-phenylethyl)phenyl)carbamate (14):** Prepared according to general procedure A using *tert*-butyl (4-iodophenyl)carbamate (0.5 mmol, 160 mg, 1.0 equiv), Na_2CO_3 (1 mmol, 106 mg, 2.0 equiv), Cl-4CzIPN (0.025 mmol, 26 mg, 5 mol%), $(TMS)_3SiOH$ (0.75 mmol, 0.230 mL, 1.5 equiv), and styrene (1.5 mmol, 0.172 mL, 3.0 equiv) in 19:1 MeCN:H_2O (5 mL). The reaction was purified by silica chromatography (0-20% EtOAc/Hex) to afford the title compound as a pale yellow solid (121 mg, 77%). The physical properties and spectral data were consistent with those reported in literature.[10]

^{1}H NMR (600 MHz, CDCl$_3$) δ 7.37 – 7.31 (m, 4H), 7.33 – 7.25 (m, 3H), 7.10 (d, *J* = 8.4 Hz, 2H), 6.43 (s, 1H), 4.85 (m, 1H), 2.99 (dd, *J* = 13.8, 4.9 Hz, 1H), 2.93 (dd, *J* = 13.8, 8.4 Hz, 1H), 1.93 (d, *J* = 2.9 Hz, 1H), 1.51 (s, 9H).

^{13}C NMR (151 MHz, CDCl$_3$) δ 152.92, 143.87, 137.07, 132.69, 130.20, 128.55, 127.74, 126.05, 118.84, 80.66, 75.49, 45.54, 28.50.

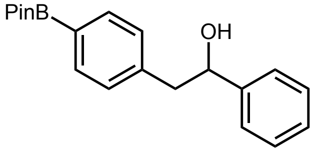

1-phenyl-2-(4-(4,4,5,5-tetramethyl-1,3,2-dioxaborolan-2-yl)phenyl)ethan-1-ol (15): Prepared according to general procedure A using 2-(4-iodophenyl)-4,4,5,5-tetramethyl-1,3,2-dioxaborolane (0.5 mmol, 165 mg, 1.0 equiv), Na_2CO_3 (1 mmol, 106 mg, 2.0 equiv), Cl-4CzIPN (0.025 mmol, 26 mg, 5 mol%), $(TMS)_3SiOH$ (0.75 mmol, 0.230 mL, 1.5 equiv), and styrene (1.5 mmol, 0.172 mL, 3.0 equiv) in 19:1 MeCN:H_2O (5 mL). The reaction was purified by silica chromatography (0-20% EtOAc/Hex) to afford the title compound as a clear oil (40.1 mg, 25%).

^{1}H NMR (400 MHz, CDCl$_3$) δ 7.75 (d, *J* = 7.9 Hz, 2H), 7.38 – 7.27 (m, 5H), 7.21 (d, *J* = 7.9 Hz, 2H), 4.91 (dd, *J* = 8.3, 5.1 Hz, 1H), 3.10 – 2.89 (m, 2H), 1.92 (s, 1H), 1.34 (s, 12H).

^{13}C NMR (151 MHz, CDCl$_3$) δ 143.87, 141.47, 135.15, 129.10, 128.58, 127.78, 126.03, 83.89, 75.41, 46.45, 25.01.

HRMS (APCI pos.) m/z: [M+H] calcd. for $C_{20}H_{24}O_3{}^{10}B$, 322.18493; found 322.18395.

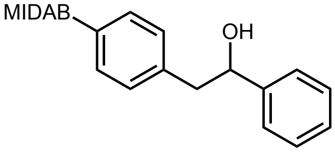

2-(4-(2-hydroxy-2-phenylethyl)phenyl)-6-methyl-1,3,6,2-dioxazaborocane-4,8-dione (16): Prepared according to general procedure B using 2-(4-iodophenyl)-6-methyl-1,3,6,2-dioxazaborocane-4,8-dione (0.3 mmol, 108 mg, 1.0 equiv), Na_2CO_3 (0.6 mmol, 63.6 mg, 2.0 equiv), Cl-4CzIPN (0.015 mmol, 15 mg, 5 mol%), $(TMS)_3SiOH$ (0.45 mmol, 0.138 mL, 1.5 equiv), and styrene (1.5 mmol, 0.172 mL, 3.0 equiv) in 19:1 MeCN:H_2O (3 mL). The reaction was purified by silica chromatography (0-5% MeOH/DCM) to afford the title compound as a pale brown solid (61.2 mg, 58%).

^{1}H NMR (600 MHz, CDCl$_3$) δ 7.45 (d, *J* = 7.8 Hz, 2H), 7.36 – 7.32 (m, 4H), 7.31 – 7.26 (m, 1H), 7.24 (d, *J* = 7.8 Hz, 2H), 4.93 – 4.89 (m, 1H), 3.91 (d, *J* = 16.4 Hz, 2H), 3.75 (d, *J* = 16.4 Hz, 2H), 3.08 – 2.96 (m, 2H), 2.55 (s, 3H), 1.97 (d, *J* = 3.0 Hz, 1H).

^{13}C NMR (151 MHz, CDCl$_3$) δ 167.30, 143.89, 140.25, 132.60, 129.76, 128.60, 127.88, 126.02, 75.38, 61.88, 47.52, 46.08.

HRMS (APCI pos.) m/z: [M+Na] calcd. for $C_{19}H_{20}O_5N^{10}B^{23}Na$, 375.13631; found 375.1352.

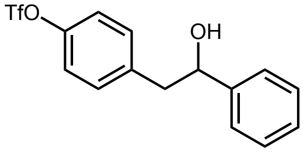

4-(2-hydroxy-2-phenylethyl)phenyl trifluoromethanesulfonate (17): Prepared according to general procedure A using 4-iodophenyl trifluoromethanesulfonate (0.5 mmol, 176 mg, 1.0 equiv), Na_2CO_3 (1 mmol, 106 mg, 2.0 equiv), Cl-4CzIPN (0.025 mmol, 26 mg, 5 mol%), $(TMS)_3SiOH$ (0.75 mmol, 0.230 mL, 1.5 equiv), and styrene (1.5 mmol, 0.172 mL, 3.0 equiv) in 19:1 MeCN:H_2O (5 mL). The reaction was purified by silica chromatography (0-30% EtOAc/Hex) to afford the title compound as a pale yellow solid (136 mg, 78%).
^{1}H NMR (400 MHz, $CDCl_3$) δ 7.38 – 7.27 (m, 5H), 7.24 (d, J = 8.8 Hz, 2H), 7.18 (d, J = 8.8 Hz, 2H), 4.93 – 4.87 (m, 1H), 3.15 – 3.00 (m, 2H), 1.97 (d, J = 2.9 Hz, 1H).
^{13}C NMR (101 MHz, $CDCl_3$) δ 148.43, 143.53, 138.94, 131.47, 128.70, 128.09, 125.98, 121.25, 118.93 (q, J = 321.1 Hz) 75.28, 45.15.
^{19}F NMR (376 MHz, $CDCl_3$) δ -72.84.
HRMS (ESI pos.) m/z: [M+Na] calcd. for $C_{15}H_{13}O_4F_3NaS$, 369.03789; found 369.03764.

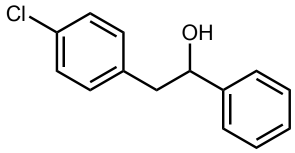

2-(4-chlorophenyl)-1-phenylethan-1-ol (18): Prepared according to general procedure A using 1-chloro-4-iodobenzene (0.5 mmol, 119 mg, 1.0 equiv), Na_2CO_3 (1 mmol, 106 mg, 2.0 equiv), Cl-4CzIPN (0.025 mmol, 26 mg, 5 mol%), $(TMS)_3SiOH$ (0.75 mmol, 0.230 mL, 1.5 equiv), and styrene (1.5 mmol, 0.172 mL, 3.0 equiv) in 19:1 MeCN:H_2O (5 mL). The reaction was purified by silica chromatography (0-30% EtOAc/Hex) to afford the title compound as a yellow oil (105 mg, 90%). The physical properties and spectral data were consistent with those reported in literature.[8]
^{1}H NMR (600 MHz, $CDCl_3$) δ 7.40 – 7.22 (m, 5H), 7.25 (d, J = 8.3 Hz, 2H), 7.10 (d, J = 8.3 Hz, 2H), 4.86 (t, J = 6.6 Hz, 1H), 2.99 (d, J = 6.6 Hz, 2H), 1.97 (broad s, 1H).
^{13}C NMR (101 MHz, $CDCl_3$) δ 143.67, 136.61, 132.53, 131.02, 128.64, 128.62, 127.93, 126.02, 75.40, 45.32.

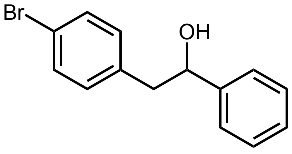

2-(4-bromophenyl)-1-phenylethan-1-ol (19): Prepared according to general procedure A using 1-bromo-4-iodobenzene (0.5 mmol, 141 mg, 1.0 equiv), Na_2CO_3 (1 mmol, 106 mg, 2.0 equiv), Cl-4CzIPN (0.025 mmol, 26 mg, 5 mol%), $(TMS)_3SiOH$ (0.75 mmol, 0.230 mL, 1.5 equiv), and styrene (1.5 mmol, 0.172 mL, 3.0 equiv) in 19:1 MeCN:H_2O (5 mL). The reaction was purified by silica chromatography (0-30% EtOAc/Hex) to afford the title compound as a yellow solid (105 mg, 76%). The physical properties and spectral data were consistent with those reported in literature.[8]
^{1}H NMR (500 MHz, $CDCl_3$) δ 7.41 – 7.27 (m, 5H), 7.26 (d, J = 8.1 Hz, 2H), 7.10 (d, J = 8.1 Hz, 2H), 4.87 (t, J = 6.7 Hz, 1H), 3.00 (d, J = 6.6 Hz, 2H), 1.96 (s, 1H).
^{13}C NMR (101 MHz, $CDCl_3$) δ 143.65, 137.14, 131.59, 131.42, 128.63, 127.94, 126.02, 120.62, 75.34, 45.38.

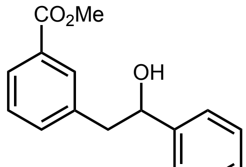

Methyl 3-(2-hydroxy-2-phenylethyl)benzoate (20): Prepared according to general procedure A using methyl 3-iodobenzoate (0.5 mmol, 131 mg, 1.0 equiv), Na_2CO_3 (1 mmol, 106 mg, 2.0 equiv), Cl-4CzIPN (0.025 mmol, 26 mg, 5 mol%), $(TMS)_3SiOH$ (0.75 mmol, 0.230 mL, 1.5 equiv), and styrene (1.5 mmol, 0.172 mL, 3.0 equiv) in 19:1 MeCN:H_2O (5 mL). The reaction was purified by silica chromatography (0-30% EtOAc/Hex) to afford the title compound as a yellow oil (121 mg, 95%).
^{1}H NMR (600 MHz, $CDCl_3$) δ 7.94 – 7.88 (m, 2H), 7.39 – 7.32 (m, 6H), 7.29 (m, 1H), 4.95 – 4.89 (m, 1H), 3.91 (s, 3H), 3.06 (d, J = 6.8 Hz, 2H), 2.01 (d, J = 2.8 Hz, 1H).

^{13}C NMR (101 MHz, CDCl$_3$) δ 167.26, 143.77, 138.69, 134.37, 130.67, 130.40, 128.64, 128.58, 127.97, 127.92, 126.00, 75.36, 52.26, 45.79.
HRMS (APCI pos.) m/z: [M+H] calcd. for $C_{16}H_{17}O_3$, 257.11722; found 257.11694.

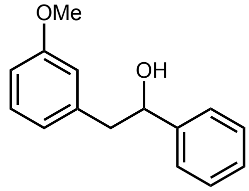

2-(3-methoxyphenyl)-1-phenylethan-1-ol (21): Prepared according to general procedure A using 1-iodo-3-methoxybenzene (0.5 mmol, 117 mg, 1.0 equiv), Na_2CO_3 (1 mmol, 106 mg, 2.0 equiv), Cl-4CzIPN (0.025 mmol, 26 mg, 5 mol%), $(TMS)_3SiOH$ (0.75 mmol, 0.230 mL, 1.5 equiv), and styrene (1.5 mmol, 0.172 mL, 3.0 equiv) in 19:1 MeCN:H_2O (5 mL). The reaction was purified by silica chromatography (0-30% EtOAc/Hex) to afford the title compound as a pale brown oil (88.3 mg, 77%). The physical properties and spectral data were consistent with those reported in literature.[11]
^{1}H NMR (500 MHz, CDCl$_3$) δ 7.41 – 7.32 (m, 4H), 7.35 – 7.26 (m, 1H), 7.23 (t, J = 7.8 Hz, 1H), 6.84 – 6.77 (m, 2H), 6.73 (m, 1H), 4.90 (dd, J = 8.6, 4.7 Hz, 1H), 3.78 (s, 3H, 3.03 (dd, J = 13.6, 4.7 Hz, 1H), 2.97 (dd, J = 13.6, 8.6 Hz, 1H).
^{13}C NMR (101 MHz, CDCl$_3$) δ 159.82, 143.89, 139.71, 129.65, 128.55, 127.75, 126.03, 121.95, 115.19, 112.29, 75.35, 55.28, 46.29.

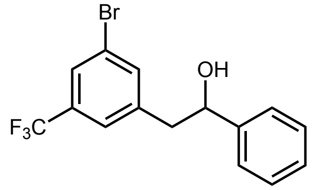

2-(3-bromo-5-(trifluoromethyl)phenyl)-1-phenylethan-1-ol (22): Prepared according to general procedure A using 1-bromo-3-iodo-5-(trifluoromethyl)benzene (0.5 mmol, 175 mg, 1.0 equiv), Na_2CO_3 (1 mmol, 106 mg, 2.0 equiv), Cl-4CzIPN (0.025 mmol, 26 mg, 5 mol%), $(TMS)_3SiOH$ (0.75 mmol, 0.230 mL, 1.5 equiv), and styrene (1.5 mmol, 0.172 mL, 3.0 equiv) in 19:1 MeCN:H_2O (5 mL). The reaction was purified by silica chromatography (0-10% EtOAc/Hex), followed by an alumina plug as described in general procedure A, to afford the title compound as a yellow oil (88.3 mg, 77%).
^{1}H NMR (600 MHz, CDCl$_3$) δ 7.63 (s, 1H), 7.53 (s, 1H), 7.39 – 7.29 (m, 6H), 4.91 (dd, J = 8.2, 4.9 Hz, 1H), 3.06 (dd, J = 13.8, 8.2 Hz, 1H), 3.01 (dd, J = 13.8, 4.9 Hz, 1H), 1.91 (broad s, 1H).
^{13}C NMR (101 MHz, CDCl$_3$) δ 143.36, 141.62, 136.19, 132.33 (q, J = 32.7 Hz), 128.80, 128.26, 126.71 (q, J = 3.9 Hz), 125.92, 125.32 (q, J = 3.7 Hz), 123.34 (q, J = 273.1 Hz), 75.01, 45.27.
^{19}F NMR (376 MHz, CDCl$_3$) δ -62.75.
HRMS (APCI pos.) m/z: [M+H] calcd. for $C_{15}H_{13}OBrF3$, 344.99399; found 344.99368.

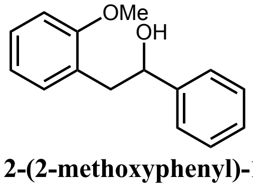

2-(2-methoxyphenyl)-1-phenylethan-1-ol (23): Prepared according to general procedure A using 1-iodo-2-methoxybenzene (0.5 mmol, 117 mg, 1.0 equiv), Na_2CO_3 (1 mmol, 106 mg, 2.0 equiv), Cl-4CzIPN (0.025 mmol, 26 mg, 5 mol%), $(TMS)_3SiOH$ (0.75 mmol, 0.230 mL, 1.5 equiv), and styrene (1.5 mmol, 0.172 mL, 3.0 equiv) in 19:1 MeCN:H_2O (5 mL). The reaction was purified by silica chromatography (0-20% EtOAc/Hex) to afford the title compound as a white solid (89.4 mg, 78%). The physical properties and spectral data were consistent with those reported in literature.[12]
^{1}H NMR (600 MHz, CDCl$_3$) δ 7.39 (d, J = 7.6 Hz, 2H), 7.36 – 7.31 (m, 2H), 7.29 – 7.21 (m, 2H), 7.08 (d, J = 7.4 Hz, 1H), 6.89 (m, 2H), 4.99 – 4.94 (m, 1H), 3.86 (s, 3H), 3.12 (dd, J = 13.7, 4.0 Hz, 1H), 2.98 (dd, J = 13.7, 8.8 Hz, 1H), 2.54 (d, J = 2.8 Hz, 1H).

^{13}C NMR (101 MHz, CDCl3) δ 157.73, 144.67, 131.65, 128.38, 128.18, 127.40, 126.77, 125.90, 120.87, 110.59, 74.44, 55.54, 41.36.

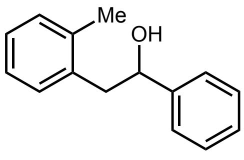

1-phenyl-2-(*o*-tolyl)ethan-1-ol (24): Prepared according to general procedure A using 1-iodo-2-methylbenzene (0.5 mmol, 109 mg, 1.0 equiv), Na_2CO_3 (1 mmol, 106 mg, 2.0 equiv), Cl-4CzIPN (0.025 mmol, 26 mg, 5 mol%), $(TMS)_3SiOH$ (0.75 mmol, 0.230 mL, 1.5 equiv), and styrene (1.5 mmol, 0.172 mL, 3.0 equiv) in 19:1 MeCN:H_2O (5 mL). The reaction was purified by silica chromatography (0-10% EtOAc/Hex), followed by an alumina plug as described in general procedure A, to afford the title compound as a yellow oil (95.0 mg, 90%). The physical properties and spectral data were consistent with those reported in literature.[13]
^{1}H NMR (600 MHz, $CDCl_3$) δ 7.40 – 7.33 (m, 4H), 7.33 – 7.24 (m, 1H), 7.23 – 7.12 (m, 4H), 4.90 (dd, *J* = 8.5, 5.0 Hz, 1H), 3.06 (dd, *J* = 13.9, 5.0 Hz, 1H), 3.01 (dd, *J* = 13.9, 8.5 Hz, 1 H), 2.31 (s, 3H), 1.99 (broad s, 1H).
^{13}C NMR (101 MHz, $CDCl_3$) δ 144.20, 136.92, 136.44, 130.60, 130.42, 128.56, 127.73, 126.90, 126.14, 125.89, 74.53, 43.50, 19.71.

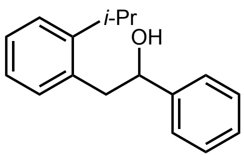

2-(2-isopropylphenyl)-1-phenylethan-1-ol (25): Prepared according to general procedure A using 1-iodo-2-isopropylbenzene (0.5 mmol, 123 mg, 1.0 equiv), Na_2CO_3 (1 mmol, 106 mg, 2.0 equiv), Cl-4CzIPN (0.025 mmol, 26 mg, 5 mol%), $(TMS)_3SiOH$ (0.75 mmol, 0.230 mL, 1.5 equiv), and styrene (1.5 mmol, 0.172 mL, 3.0 equiv) in 19:1 MeCN:H_2O (5 mL). The reaction was purified by silica chromatography (0-20% EtOAc/Hex) to afford the title compound as a clear oil (76.1 mg, 63%).
^{1}H NMR (600 MHz, $CDCl_3$) δ 7.42 – 7.32 (m, 4H), 7.33 – 7.27 (m, 2H), 7.27 – 7.22 (m, 1H), 7.18 – 7.08 (m, 2H), 4.90 – 4.84 (m, 1H), 3.21 (hept, *J* = 7.0 Hz, 1H), 3.11 (dd, *J* = 13.9, 5.0 Hz, 1H), 3.05 (dd, *J* = 13.9, 8.6 Hz, 1H), 1.97 (d, *J* = 2.5 Hz, 1H), 1.23 (d, *J* = 6.9 Hz, 3H), 1.19 (d, *J* = 6.9 Hz, 2H).
^{13}C NMR (101 MHz, $CDCl_3$) δ 147.61, 144.12, 134.75, 130.75, 128.65, 128.57, 127.77, 127.31, 125.95, 125.79, 125.72, 75.42, 42.86, 28.95, 24.22, 24.06.
HRMS (ESI pos.) m/z: [M+Na] calcd. for $C_{17}H_{20}O_4Na$, 263.14064; found 263.14051.

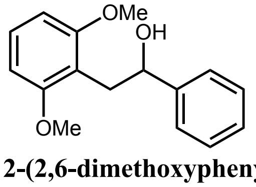

2-(2,6-dimethoxyphenyl)-1-phenylethan-1-ol (26): Prepared according to general procedure A using 2-iodo-1,3-dimethoxybenzene (0.5 mmol, 132 mg, 1.0 equiv), Na_2CO_3 (1 mmol, 106 mg, 2.0 equiv), Cl-4CzIPN (0.025 mmol, 26 mg, 5 mol%), $(TMS)_3SiOH$ (0.75 mmol, 0.230 mL, 1.5 equiv), and styrene (1.5 mmol, 0.172 mL, 3.0 equiv) in 19:1 MeCN:H_2O (5 mL). The reaction was purified by silica chromatography (0-30% EtOAc/Hex) to afford the title compound as a white solid (47.0 mg, 36%).
^{1}H NMR (600 MHz, $CDCl_3$) δ 7.43 – 7.38 (m, 2H), 7.33 (t, *J* = 7.5 Hz, 2H), 7.26 – 7.20 (m, 1H), 7.18 (t, *J* = 8.4 Hz, 1H), 6.56 (d, *J* = 8.4 Hz, 2H), 4.89 (dd, *J* = 9.0, 3.5 Hz, 1H), 3.79 (s, 6H), 3.16 (dd, *J* = 13.7, 3.6 Hz, 1H), 3.09 (dd, *J* = 13.7, 9.0 Hz, 1H), 3.03 (s, 1H).
^{13}C NMR (101 MHz, $CDCl_3$) δ 158.63, 145.44, 128.14, 127.92, 127.05, 125.81, 115.21, 104.03, 74.81, 55.91, 33.74.
HRMS (APCI pos.) m/z: [M+H] calcd. for $C_{16}H_{19}O_3$, 259.13287; found 259.13283.

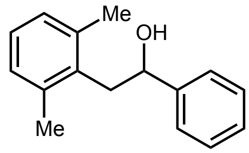

2-(2,6-dimethylphenyl)-1-phenylethan-1-ol (27): Prepared according to general procedure A using 2-iodo-1,3-dimethylbenzene (0.5 mmol, 116 mg, 1.0 equiv), Na_2CO_3 (1 mmol, 106 mg, 2.0 equiv), Cl-4CzIPN (0.025 mmol, 26 mg, 5 mol%), $(TMS)_3SiOH$ (0.75 mmol, 0.230 mL, 1.5 equiv), and styrene (1.5 mmol, 0.172 mL, 3.0 equiv) in 19:1 MeCN:H_2O (5 mL). The reaction was purified by silica chromatography (0-20% EtOAc/Hex) to afford the title compound as a clear oil (66.6 mg, 59%).
^{1}H NMR (600 MHz, $CDCl_3$) δ 7.40 – 7.32 (m, 4H), 7.32 – 7.26 (m, 1H), 7.08 – 6.98 (m, 3H), 4.93 (dd, J = 9.0, 4.9 Hz, 1H), 3.17 (dd, J = 13.9, 9.0 Hz, 1H), 2.99 (dd, J = 14.0, 4.8 Hz, 1H), 2.33 (s, 6H), 1.81 (broad s, 1H).
^{13}C NMR (101 MHz, $CDCl_3$) δ 144.62, 137.58, 135.07, 128.57, 128.52, 127.69, 126.63, 125.70, 74.17, 39.94, 20.55.
HRMS (APCI pos.) m/z: [M+H] calcd. for $C_{16}H_{17}O$, 225.12739; found 225.12721.

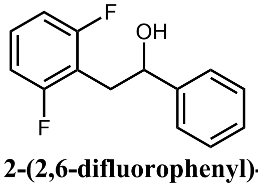

2-(2,6-difluorophenyl)-1-phenylethan-1-ol (28): Prepared according to general procedure A using 1,3-difluoro-2-iodobenzene (0.5 mmol, 120 mg, 1.0 equiv), Na_2CO_3 (1 mmol, 106 mg, 2.0 equiv), Cl-4CzIPN (0.025 mmol, 26 mg, 5 mol%), $(TMS)_3SiOH$ (0.75 mmol, 0.230 mL, 1.5 equiv), and styrene (1.5 mmol, 0.172 mL, 3.0 equiv) in 19:1 MeCN:H_2O (5 mL). The reaction was purified by silica chromatography (0-30% EtOAc/Hex) to afford the title compound as a pale yellow solid (99.2 mg, 85%).
^{1}H NMR (600 MHz, $CDCl_3$) δ 7.37 (d, J = 8.3, 2H), 7.33 (t, J = 7.6 Hz, 2H), 7.30 – 7.23 (m, 1H), 7.16 (tt, J = 8.4, 6.4 Hz, 1H), 6.88 – 6.80 (m, 2H), 4.96 (dd, J = 8.7, 5.1 Hz, 1H), 3.14 (dd, J = 13.7, 8.6, 1H), 3.05 (dd, J = 13.8, 5.0, 1H), 1.96 (s, 1H).
^{13}C NMR (101 MHz, $CDCl_3$) δ 161.97 (dd, J = 247.0, 8.7 Hz), 143.80, 128.57, 128.29 (t, J = 10.3 Hz), 127.96, 125.84, 114.24 (t, J = 20.1 Hz), 111.19 (ddd, J = 26.0, 19.1, 6.82), 73.89, 32.66.
^{19}F NMR (376 MHz, $CDCl_3$) δ -114.69 – -114.78 (m).
HRMS (ESI pos.) m/z: [M+Na] calcd. for $C_{14}H_{12}OF_2Na$, 257.07484; found 257.07487.

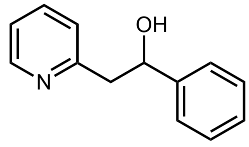

1-phenyl-2-(pyridin-2-yl)ethan-1-ol (29): Prepared according to general procedure B with a 48 hour reaction time using 2-iodopyridine (0.5 mmol, 103 mg, 1.0 equiv), Na_2CO_3 (1 mmol, 106 mg, 2.0 equiv), Cl-4CzIPN (0.025 mmol, 26 mg, 5 mol%), $(TMS)_3SiOH$ (0.75 mmol, 0.230 mL, 1.5 equiv), and styrene (1.5 mmol, 0.172 mL, 3.0 equiv) in 19:1 MeCN:H_2O (5 mL). The reaction was purified by silica chromatography (0-50% EtOAc/Hex) to afford the title compound as a brown solid (41.3 mg, 42%). The physical properties and spectral data were consistent with those reported in literature.[14]
^{1}H NMR (500 MHz, $CDCl_3$) δ 8.55 (d, J = 4.9, 1H), 7.63 (td, J = 7.8, 1.8 Hz, 1H), 7.43 (d, J = 8.0 Hz, 2H), 7.35 (t, J = 7.5 Hz, 2H), 7.35 – 7.24 (m, 1H), 7.24 – 7.14 (m, 1H), 7.12 (d, J = 7.8 Hz, 1H), 5.18 (dd, J = 8.5, 3.7 Hz, 1H), 3.20 – 3.07 (m, 2H).
^{13}C NMR (101 MHz, $CDCl_3$) δ 159.92, 148.71, 144.18, 137.04, 128.47, 127.43, 125.98, 123.96, 121.89, 73.48, 45.78.

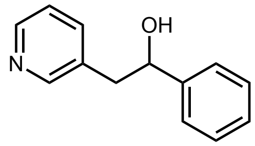

1-phenyl-2-(pyridin-3-yl)ethan-1-ol (30): Prepared according to general procedure B using 3-iodopyridine (0.5 mmol, 103 mg, 1.0 equiv), Na_2CO_3 (1 mmol, 106 mg, 2.0 equiv), Cl-4CzIPN (0.025 mmol, 26 mg, 5 mol%),

$(TMS)_3SiOH$ (0.75 mmol, 0.230 mL, 1.5 equiv), and styrene (1.5 mmol, 0.172 mL, 3.0 equiv) in 19:1 MeCN:H_2O (5 mL). The reaction was purified by silica chromatography (0-5% MeOH/DCM) to afford the title compound as a brown solid (65.9 mg, 66%). The physical properties and spectral data were consistent with those reported in literature.[14]

^{1}H NMR (600 MHz, CDCl$_3$) δ 8.41 (d, *J* = 4.8 Hz, 1H), 8.37 (s, 1H), 7.47 (d, *J* = 7.8 Hz, 1H), 7.38 – 7.24 (m, 5H), 7.19 (dd, *J* = 7.8, 4.8 Hz, 1H), 4.90 (dd, *J* = 7.8, 5.3 Hz, 1H), 3.04 (dd, *J* = 13.9, 7.8 Hz, 1H), 3.00 (dd, *J* = 13.9, 5.3 Hz, 1H), 2.65 (broad s, 1H).

^{13}C NMR (101 MHz, CDCl$_3$) δ 150.60, 147.66, 143.59, 137.58, 133.95, 128.69, 128.05, 126.02, 123.39, 75.04, 42.97.

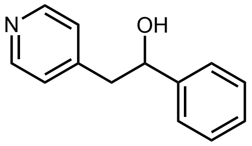

1-phenyl-2-(pyridin-4-yl)ethan-1-ol (31): Prepared according to general procedure B using 4-iodopyridine (0.5 mmol, 103 mg, 1.0 equiv), Na_2CO_3 (1 mmol, 106 mg, 2.0 equiv), Cl-4CzIPN (0.025 mmol, 26 mg, 5 mol%), $(TMS)_3SiOH$ (0.75 mmol, 0.230 mL, 1.5 equiv), and styrene (1.5 mmol, 0.172 mL, 3.0 equiv) in 19:1 MeCN:H_2O (5 mL). The reaction was purified by silica chromatography (0-5% MeOH/DCM) to afford the title compound as a pale yellow solid (75.4 mg, 76%). The physical properties and spectral data were consistent with those reported in literature.[14]

^{1}H NMR (600 MHz, CDCl$_3$) δ 8.42 (d, *J* = 5.9 Hz, 2H), 7.36 – 7.25 (m, 5H), 7.09 (d, *J* = 5.9 Hz, 2H), 4.92 (dd, *J* = 8.1, 5.1 Hz, 1H), 3.03 (dd, *J* = 13.6, 8.1 Hz, 1H), 2.98 (dd, *J* = 13.7, 5.1 Hz, 1H), 2.63 (broad s, 1H).

^{13}C NMR (101 MHz, CDCl$_3$) δ 149.52, 147.67, 143.56, 128.71, 128.09, 125.98, 125.12, 74.64, 45.23.

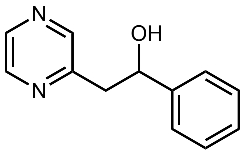

1-phenyl-2-(pyrazin-2-yl)ethan-1-ol (32): Prepared according to general procedure B using 2-iodopyrazine (0.5 mmol, 103 mg, 1.0 equiv), Na_2CO_3 (1 mmol, 106 mg, 2.0 equiv), Cl-4CzIPN (0.025 mmol, 26 mg, 5 mol%), $(TMS)_3SiOH$ (0.75 mmol, 0.230 mL, 1.5 equiv), and styrene (1.5 mmol, 0.172 mL, 3.0 equiv) in 19:1 MeCN:H_2O (5 mL). The reaction was purified by silica chromatography (0-70% EtOAc/Hex) to afford the title compound as a yellow solid (51.4 mg, 51%). The physical properties and spectral data were consistent with those reported in literature.[15]

^{1}H NMR (500 MHz, CDCl$_3$) δ 8.52 (dd, *J* = 2.6, 1.6 Hz, 1H), 8.48 (d, *J* = 2.6 Hz, 1H), 8.44 (d, *J* = 1.6 Hz, 1H), 7.44 – 7.39 (m, 2H), 7.42 – 7.32 (m, 2H), 7.32 – 7.25 (m, 1H), 5.20 (dd, *J* = 8.2, 4.1 Hz, 2H), 3.26 – 3.13 (m, 2H), 1.60 (broad s, 1H)

^{13}C NMR (101 MHz, CDCl$_3$) δ 155.14, 145.62, 143.51, 143.48, 143.04, 128.68, 127.86, 125.87, 73.29, 43.63.

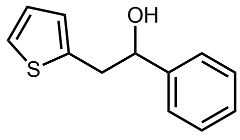

1-phenyl-2-(thiophen-2-yl)ethan-1-ol (33): Prepared according to general procedure A using 2-iodothiophene (0.5 mmol, 105 mg, 1.0 equiv), Na_2CO_3 (1 mmol, 106 mg, 2.0 equiv), Cl-4CzIPN (0.025 mmol, 26 mg, 5 mol%), $(TMS)_3SiOH$ (0.75 mmol, 0.230 mL, 1.5 equiv), and styrene (1.5 mmol, 0.172 mL, 3.0 equiv) in 19:1 MeCN:H_2O (5 mL). The reaction was purified by silica chromatography (0-10% EtOAc/Hex), followed by an alumina plug as described in general procedure A, to afford the title compound as a brown oil (61.3 mg, 60%). The physical properties and spectral data were consistent with those reported in literature.[14]

^{1}H NMR (500 MHz, CDCl$_3$) δ 7.47 – 7.32 (m, 4H), 7.35 – 7.26 (m, 1H), 7.19 (dd, *J* = 5.1, 1.2 Hz, 1H), 6.96 (dd, *J* = 5.1, 3.4 Hz, 1H), 6.87 – 6.84 (m, 1H), 4.91 (dd, *J* = 7.8, 5.2 Hz, 1H), 3.26 (dd, *J* = 11.9, 5.5 Hz, 1H), 3.22 (dd, *J* = 11.9, 7.9 Hz, 1H) – 3.18 (m, 2H), 2.43 – 1.65 (broad s, 1H).

^{13}C NMR (101 MHz, CDCl$_3$) δ 143.38, 140.20, 128.64, 127.95, 127.08, 126.45, 126.02, 124.58, 75.17, 40.18.

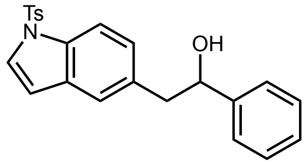

1-phenyl-2-(1-tosyl-1*H*-indol-5-yl)ethan-1-ol (34): Prepared according to general procedure A using 5-iodo-1-tosyl-1*H*-indole (0.5 mmol, 199 mg, 1.0 equiv), Na_2CO_3 (1 mmol, 106 mg, 2.0 equiv), Cl-4CzIPN (0.025 mmol, 26 mg, 5 mol%), $(TMS)_3SiOH$ (0.75 mmol, 0.230 mL, 1.5 equiv), and styrene (1.5 mmol, 0.172 mL, 3.0 equiv) in 19:1 MeCN:H_2O (5 mL). The reaction was purified by silica chromatography (0-30% EtOAc/Hex) to afford the title compound as a yellow solid (122 mg, 62%).

^{1}H NMR (600 MHz, $CDCl_3$) δ 7.90 (d, *J* = 8.6 Hz, 1H), 7.76 (d, *J* = 8.6 Hz, 2H), 7.55 (d, *J* = 3.7 Hz, 1H), 7.38 – 7.31 (m, 5H), 7.33 – 7.20 (m, 1H), 7.23 – 7.17 (m, 2H), 7.15 (dd, *J* = 8.6, 1.7 Hz, 1H), 6.60 (dd, *J* = 3.7, 0.8 Hz, 1H), 4.89 (dd, *J* = 9.0, 4.3 Hz, 1H), 3.09 (dd, *J* = 13.8, 4.3 Hz, 1H), 3.01 (dd, *J* = 13.8, 9.0 Hz, 1H), 2.34 (s, 3H), 1.89 (s, 1H).

^{13}C NMR (151 MHz, $CDCl_3$) δ 145.06, 143.92, 135.46, 133.92, 133.25, 131.23, 130.03, 128.60, 127.81, 126.98, 126.80, 126.27, 126.00, 122.18, 113.67, 109.03, 75.61, 46.09, 21.72.

HRMS (APCI pos.) m/z: [M+H] calcd. for $C_{23}H_{22}O_3NS$, 392.13149; found 392.13128.

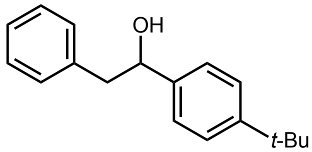

1-(4-(*tert*-butyl)phenyl)-2-phenylethan-1-ol (35): Prepared according to general procedure A using iodobenzene (0.5 mmol, 102 mg, 1.0 equiv), Na_2CO_3 (1 mmol, 106 mg, 2.0 equiv), Cl-4CzIPN (0.025 mmol, 26 mg, 5 mol%), $(TMS)_3SiOH$ (0.75 mmol, 0.230 mL, 1.5 equiv), and 1-(*tert*-butyl)-4-vinylbenzene (1.5 mmol, 0.270 mL, 3.0 equiv) in 19:1 MeCN:H_2O (5 mL). The reaction was purified by silica chromatography (30-100% DCM/Hex) to afford the title compound as a yellow oil (126 mg, 99%). The physical properties and spectral data were consistent with those reported in literature.[16]

^{1}H NMR (500 MHz, $CDCl_3$) δ 7.40 (d, *J* = 8.3 Hz, 2H), 7.40 – 7.28 (m, 4H), 7.30 – 7.18 (m, 3H), 4.89 (dd, *J* = 9.0, 4.3 Hz, 1H), 3.06 (dd, *J* = 13.7, 4.4 Hz, 1H), 2.99 (dd, *J* = 13.7, 9.0 Hz, 1H), 1.92 (broad s, 1H), 1.34 (s, 9H).

^{13}C NMR (101 MHz, $CDCl_3$) δ 150.77, 141.05, 138.52, 129.62, 128.67, 126.73, 125.75, 125.51, 75.28, 46.11, 34.69, 31.52.

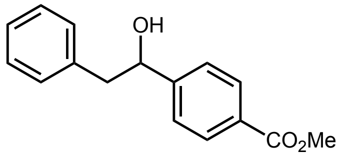

Methyl 4-(1-hydroxy-2-phenylethyl)benzoate (36): Prepared according to general procedure A using iodobenzene (0.5 mmol, 102 mg, 1.0 equiv), Na_2CO_3 (1 mmol, 106 mg, 2.0 equiv), Cl-4CzIPN (0.025 mmol, 26 mg, 5 mol%), $(TMS)_3SiOH$ (0.75 mmol, 0.230 mL, 1.5 equiv), and methyl 4-vinylbenzoate (1.5 mmol, 243 mg, 3.0 equiv) in 19:1 MeCN:H_2O (5 mL). The reaction was purified by silica chromatography (0-30% EtOAc/Hex) to afford the title compound as a pale yellow solid (102 mg, 80%). The physical properties and spectral data were consistent with those reported in literature.[17]

^{1}H NMR (500 MHz, $CDCl_3$) δ 8.01 (d, *J* = 8.2 Hz, 2H), 7.45 – 7.33 (m, 2H), 7.33 – 7.26 (m, 2H), 7.28 – 7.22 (m, 1H), 7.20 – 7.11 (m, 2H), 4.96 (dd, *J* = 8.5, 5.0 Hz, 1H), 3.92 (s, 3H), 3.05 (dd, *J* = 13.7, 4.9 Hz, 1H), 2.97 (dd, *J* = 13.7, 8.4 Hz, 1H), 2.14 (broad s, 1H).

^{13}C NMR (101 MHz, $CDCl_3$) δ 167.10, 148.96, 137.48, 129.84, 129.65, 129.45, 128.73, 126.95, 125.97, 74.98, 52.23, 46.16.

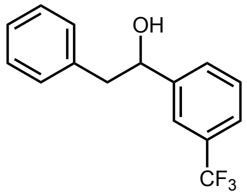

2-phenyl-1-(3-(trifluoromethyl)phenyl)ethan-1-ol (37): Prepared according to general procedure A using iodobenzene (0.5 mmol, 102 mg, 1.0 equiv), Na_2CO_3 (1 mmol, 106 mg, 2.0 equiv), Cl-4CzIPN (0.025 mmol, 26 mg, 5 mol%), $(TMS)_3SiOH$ (0.75 mmol, 0.230 mL, 1.5 equiv), and 1-(trifluoromethyl)-3-vinylbenzene (1.5 mmol, 0.222 mL, 3.0 equiv) in 19:1 MeCN:H_2O (5 mL). The reaction was purified by silica chromatography (0-10% EtOAc/Hex) to afford the title compound as a yellow solid (97.0 mg, 73%).

^{1}H NMR (500 MHz, CDCl$_3$) δ 7.63 (s, 1H), 7.61 – 7.50 (t, *J* = 7.4 Hz, 2H), 7.46 (t, *J* = 7.7 Hz, 1H), 7.35 – 7.30 (m, 2H), 7.29 – 7.24 (m, 1H), 7.22 – 7.15 (m, 2H), 4.97 (dd, *J* = 8.8, 4.6 Hz, 1H), 3.05 (dd, *J* = 13.7, 4.6 Hz, 1H), 2.96 (dd, *J* = 13.7, 8.8 Hz, 1H), 2.04 (s, 1H).

^{13}C NMR (151 MHz, CDCl$_3$) δ 144.81, 137.42, 130.88 (q, *J* = 32.2 Hz), 129.65, 129.40, 128.95, 128.84, 127.08, 124.52 (q, *J* = 3.8 Hz), 124.28 (q, *J* = 273.1 Hz), 122.86 (q, *J* = 3.8 Hz), 74.82, 46.37.

^{19}F NMR (376 MHz, CDCl$_3$) δ -62.57.

HRMS (ESI neg.) m/z: [M-H] calcd. for $C_{15}H_{12}OF_3$, 267.08348; found 267.08359.

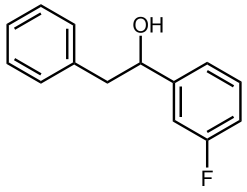

1-(3-fluorophenyl)-2-phenylethan-1-ol (38): Prepared according to general procedure A using iodobenzene (0.5 mmol, 102 mg, 1.0 equiv), Na_2CO_3 (1 mmol, 106 mg, 2.0 equiv), Cl-4CzIPN (0.025 mmol, 26 mg, 5 mol%), $(TMS)_3SiOH$ (0.75 mmol, 0.230 mL, 1.5 equiv), and 1-fluoro-3-vinylbenzene (1.5 mmol, 0.179 mL, 3.0 equiv) in 19:1 MeCN:H_2O (5 mL). The reaction was purified by silica chromatography (0-10% EtOAc/Hex) to afford the title compound as a yellow oil (106 mg, 98%).

^{1}H NMR (600 MHz, CDCl$_3$) δ 7.34 – 7.27 (m, 3H), 7.28 – 7.21 (m, 1H), 7.20 – 7.15 (m, 2H), 7.11 – 7.06 (m, 2H), 6.96 (tdd, *J* = 8.4, 2.6, 1.0 Hz, 1H), 4.92 – 4.87 (m, 1H), 3.04 (dd, *J* = 13.7, 4.7 Hz, 1H), 2.96 (dd, *J* = 13.7, 8.7 Hz, 1H), 2.03 (d, *J* = 2.7, 1H).

^{13}C NMR (101 MHz, CDCl$_3$) δ 163.07 (d, *J* = 245.9 Hz), 146.57 (d, *J* = 6.8 Hz), 137.66, 130.01 (d, *J* = 8.1 Hz), 129.63, 128.75, 126.95, 121.60 (d, *J* = 2.9 Hz), 114.52 (d, *J* = 21.2 Hz), 112.97 (d, *J* = 21.9 Hz), 74.79 (d, *J* = 1.8 Hz), 46.19.

^{19}F NMR (376 MHz, CDCl$_3$) δ -112.88 – 112.98 (m).

HRMS (APCI pos.) m/z: [M+H] calcd. for $C_{14}H_{12}OF$, 215.08667; found 215.08673.

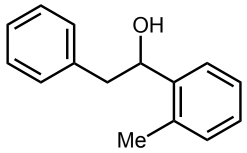

2-phenyl-1-(*o*-tolyl)ethan-1-ol (39): Prepared according to general procedure A using iodobenzene (0.5 mmol, 102 mg, 1.0 equiv), Na_2CO_3 (1 mmol, 106 mg, 2.0 equiv), Cl-4CzIPN (0.025 mmol, 26 mg, 5 mol%), $(TMS)_3SiOH$ (0.75 mmol, 0.230 mL, 1.5 equiv), and 1-methyl-2-vinylbenzene (1.5 mmol, 0.194 mL, 3.0 equiv) in 19:1 MeCN:H_2O (5 mL). The reaction was purified by silica chromatography (0-20% EtOAc/Hex) to afford the title compound as a pale yellow solid (78.1 mg, 74%). The physical properties and spectral data were consistent with those reported in literature.[16]

^{1}H NMR (600 MHz, CDCl$_3$) δ 7.57 (d, *J* = 7.6, 1H), 7.32 (t, *J* = 7.4 Hz, 2H), 7.29 – 7.21 (m, 4H), 7.20 (dt, *J* = 7.5, 1.4 Hz, 1H) 7.13 (d, *J* = 7.6, 1H), 5.13 (dd, *J* = 8.8, 4.2 Hz, 1H), 3.02 (dd, *J* = 13.7, 4.2 Hz, 1H), 2.93 (dd, *J* = 13.8, 8.8 Hz, 1H), 2.29 (s, 3H), 1.88 (s, 1H).

^{13}C NMR (101 MHz, CDCl$_3$) δ 142.17, 138.50, 134.51, 130.43, 129.59, 128.70, 127.45, 126.78, 126.51, 125.37, 71.88, 45.17, 19.15.

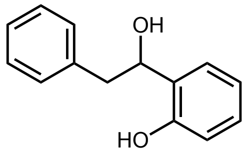

2-(1-hydroxy-2-phenylethyl)phenol (40): Prepared according to general procedure B using iodobenzene (0.5 mmol, 102 mg, 1.0 equiv), Na_2CO_3 (1 mmol, 106 mg, 2.0 equiv), Cl-4CzIPN (0.025 mmol, 26 mg, 5 mol%), $(TMS)_3SiOH$ (0.75 mmol, 0.230 mL, 1.5 equiv), and 2-vinylphenol (1.5 mmol, 180 mg, 3.0 equiv) in 19:1 MeCN:H_2O (5 mL). The reaction was purified by silica chromatography (0-30% EtOAc/Hex) to afford the title compound as a pale yellow solid (70.6 mg, 66%). The physical properties and spectral data were consistent with those reported in literature.[18]
^{1}H NMR (600 MHz, CDCl$_3$) δ 7.98 (s, 1H), 7.35 – 7.31 (m, 2H), 7.29 – 7.25 (m, 1H), 7.23 – 7.21 (m, 2H), 7.18 (t, *J* = 7.8 Hz, 1H), 6.93 (d, *J* = 7.5 Hz, 1H), 6.89 (d, *J* = 8.2, 1H), 6.82 (tt, *J* = 7.4, 1.1 Hz, 1H), 5.04 – 4.99 (m, 1H), 3.11 (d, *J* = 7.1 Hz, 2H), 2.56 (d, *J* = 2.7, 1H).
^{13}C NMR (101 MHz, CDCl$_3$) δ 155.54, 137.22, 129.52, 129.11, 128.83, 127.22, 127.07, 126.26, 119.82, 117.38, 77.02, 44.21.

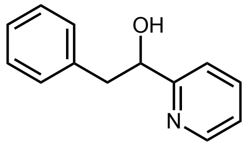

2-phenyl-1-(pyridin-2-yl)ethan-1-ol (41): Prepared according to general procedure B using iodobenzene (0.5 mmol, 102 mg, 1.0 equiv), Na_2CO_3 (1 mmol, 106 mg, 2.0 equiv), Cl-4CzIPN (0.025 mmol, 26 mg, 5 mol%), $(TMS)_3SiOH$ (0.75 mmol, 0.230 mL, 1.5 equiv), and 2-vinylpyridine (1.5 mmol, 158 mg, 3.0 equiv) in 19:1 MeCN:H_2O (5 mL). The reaction was purified by silica chromatography (0-70% EtOAc/Hex) to afford the title compound as a pale brown solid (70.8 mg, 71%). The physical properties and spectral data were consistent with those reported in literature.[19]
^{1}H NMR (500 MHz, CDCl$_3$) δ 8.55 (d, *J* = 4.9, 1H), 7.63 (dt, *J* = 7.6, 1.7 Hz, 1H), 7.30 – 7.25 (m, 2H), 7.24 – 7.18 (m, 2H), 7.18 – 7.14 (m, 2H), 7.12 (d, *J* = 7.8 Hz, 1H), 4.98 (dd, *J* = 7.6, 5.3 Hz, 1H), 3.93 (s, 1H), 3.12 (dd, *J* = 13.6, 5.3 Hz, 1H), 3.03 (dd, *J* = 13.6, 7.6 Hz, 1H).
^{13}C NMR (101 MHz, CDCl$_3$) δ 161.37, 148.51, 137.88, 136.57, 129.74, 128.47, 126.60, 122.57, 120.87, 74.18, 45.33.

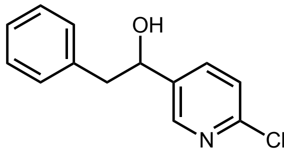

1-(6-chloropyridin-3-yl)-2-phenylethan-1-ol (42): Prepared according to general procedure B using iodobenzene (0.5 mmol, 102 mg, 1.0 equiv), Na_2CO_3 (1 mmol, 106 mg, 2.0 equiv), Cl-4CzIPN (0.025 mmol, 26 mg, 5 mol%), $(TMS)_3SiOH$ (0.75 mmol, 0.230 mL, 1.5 equiv), and 2-chloro-5-vinylpyridine (1.5 mmol, 209 mg, 3.0 equiv) in 19:1 MeCN:H_2O (5 mL). The reaction was purified by silica chromatography (0-50% EtOAc/Hex) to afford the title compound as a white solid (114 mg, 98%).
^{1}H NMR (600 MHz, CDCl$_3$) δ 8.26 (d, *J* = 2.5 Hz, 1H), 7.61 (dd, *J* = 8.2, 2.5 Hz, 1H), 7.32 – 7.20 (m, 4H), 7.16 – 7.11 (m, 2H), 4.91 (dd, *J* = 8.2, 5.3 Hz, 1H), 2.99 (dd, *J* = 13.6, 5.3 Hz, 1H), 2.95 (dd, *J* = 13.6, 8.3 Hz, 1H), 2.32 (s, 1H).
^{13}C NMR (101 MHz, CDCl$_3$) δ 150.62, 147.75, 138.09, 136.79, 136.73, 129.64, 128.91, 127.22, 124.16, 72.48, 46.12.
HRMS (APCI pos.) m/z: [M+H] calcd. for $C_{13}H_{13}ONCl$, 234.06802; found 234.06814.

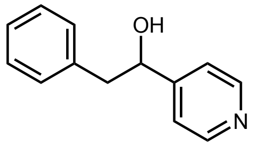

2-phenyl-1-(pyridin-4-yl)ethan-1-ol (43): Prepared according to general procedure B using iodobenzene (0.5 mmol, 102 mg, 1.0 equiv), Na_2CO_3 (1 mmol, 106 mg, 2.0 equiv), Cl-4CzIPN (0.025 mmol, 26 mg, 5 mol%), $(TMS)_3SiOH$ (0.75 mmol, 0.230 mL, 1.5 equiv), and 4-vinylpyridine (1.5 mmol, 158 mg, 3.0 equiv) in 19:1 MeCN:H_2O (5 mL). The reaction was purified by silica chromatography (0-50% EtOAc/Hex) to afford the title compound as a pale brown solid (85.6 mg, 86%). The physical properties and spectral data were consistent with those reported in literature.[20]
^{1}H NMR (500 MHz, $CDCl_3$) δ 8.55 (d, *J* = 5.5 Hz, 2H), 7.36 – 7.28 (m, 2H), 7.31 – 7.23 (m, 3H), 7.21 – 7.15 (m, 2H), 4.91 (dd, *J* = 8.6, 4.6 Hz, 1H), 3.05 (dd, *J* = 13.7, 4.6 Hz, 1H), 2.94 (dd, *J* = 13.7, 8.6 Hz, 1H).
^{13}C NMR (101 MHz, $CDCl_3$) δ 152.91, 149.74, 137.09, 129.66, 128.81, 127.11, 121.05, 73.86, 45.85.

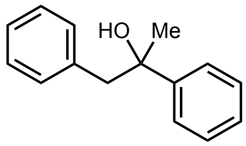

1,2-diphenylpropan-2-ol (44): Prepared according to general procedure A using iodobenzene (0.5 mmol, 102 mg, 1.0 equiv), Na_2CO_3 (1 mmol, 106 mg, 2.0 equiv), Cl-4CzIPN (0.025 mmol, 26 mg, 5 mol%), $(TMS)_3SiOH$ (0.75 mmol, 0.230 mL, 1.5 equiv), and prop-1-en-2-ylbenzene (1.5 mmol, 0.195 mL, 3.0 equiv) in 19:1 MeCN:H_2O (5 mL). The reaction was purified by silica chromatography (0-50% EtOAc/Hex) to afford the title compound as a white solid (102 mg, 96%). The physical properties and spectral data were consistent with those reported in literature.[21]
^{1}H NMR (600 MHz, $CDCl_3$) δ 7.42 – 7.37 (m, 2H), 7.32 (t, *J* = 7.6 Hz, 2H), 7.28 – 7.17 (m, 4H), 7.02 – 6.96 (m, 2H), 3.13 (d, *J* = 13.3 Hz, 1H), 3.02 (d, *J* = 13.4 Hz, 1H), 1.86 (s, 1H), 1.56 (s, 3H).
^{13}C NMR (101 MHz, $CDCl_3$) δ 147.68, 136.85, 130.74, 128.20, 128.19, 126.80, 126.79, 125.10, 74.58, 50.62, 29.53.

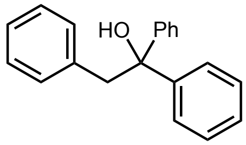

1,1,2-triphenylethan-1-ol (45): Prepared according to general procedure A using iodobenzene (0.5 mmol, 102 mg, 1.0 equiv), Na_2CO_3 (1 mmol, 106 mg, 2.0 equiv), Cl-4CzIPN (0.025 mmol, 26 mg, 5 mol%), $(TMS)_3SiOH$ (0.75 mmol, 0.230 mL, 1.5 equiv), and ethene-1,1-diyldibenzene (1.5 mmol, 0.265 mL, 3.0 equiv) in 19:1 MeCN:H_2O (5 mL). The reaction was purified by silica chromatography (0-50% EtOAc/Hex) to afford the title compound as a yellow solid (136 mg, 99%). The physical properties and spectral data were consistent with those reported in literature.[22]
^{1}H NMR (500 MHz, $CDCl_3$) δ 7.51 – 7.36 (m, 4H), 7.35 – 7.28 (m, 4H), 7.27 – 7.22 (m, 2H), 7.21 – 7.14 (m, 3H) 6.91 (dd, *J* = 7.6, 2.0 Hz, 2H), 3.67 (s, 2H), 2.33 (s, 1H).
^{13}C NMR (101 MHz, $CDCl_3$) δ 146.68, 135.91, 131.02, 128.21, 128.19, 127.02, 126.93, 126.31, 78.01, 48.07.

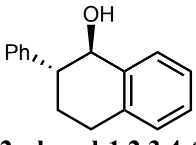

2-phenyl-1,2,3,4-tetrahydronaphthalen-1-ol (46): Prepared according to general procedure A using iodobenzene (0.5 mmol, 102 mg, 1.0 equiv), Na_2CO_3 (1 mmol, 106 mg, 2.0 equiv), Cl-4CzIPN (0.025 mmol, 26 mg, 5 mol%), $(TMS)_3SiOH$ (0.75 mmol, 0.230 mL, 1.5 equiv), and 1,2-dihydronaphthalene (1.5 mmol, 195 mg, 3.0 equiv) in 19:1 MeCN:H_2O (5 mL). The reaction was purified by silica chromatography (50-100% DCM/Hex) to afford the title compound as a white solid (34.7 mg, 31%). The compound was characterized as a mixture of diastereomers with the diastereomeric ratio determined by ^{1}H NMR (4.9:1.0 dr, *denotes major diastereomer, #denotes minor diastereomer). The minor diastereomer has been characterized and the spectral properties are consisted with literature.[23]
^{1}H NMR (500 MHz, $CDCl_3$) δ 7.63 (d, *J* = 7.8 Hz, 1H*), 7.42 – 7.34 (m, 3H), 7.33 – 7.29 (m, 3H), 7.27 – 7.18 (m, 4H), 7.14 (d, *J* = 7.4 Hz, 1H*), 4.88 (dd, *J* = 9.5, 4.7 Hz, 1H*), 4.81 (apparent t, *J* = 3.21 Hz, 1H#), 3.13 (dt, *J* = 12.9, 2.9 Hz, 1H#), 3.09 – 2.98 (m, 1H), 2.97 – 2.86 (m, 2H), 2.46 (qd, *J* = 12.6, 5.4 Hz, 1H#), 2.17 – 2.07 (m, 2H), 2.00 – 1.94 (m, 1H#), 1.90 (d, *J* = 4.7 Hz, 1H*), 1.57 (d, *J* = 3.8 Hz, 1H#).
^{13}C NMR (151 MHz, $CDCl_3$) δ 143.39, 142.70, 138.83, 137.73, 136.86, 136.49, 130.57, 129.23, 128.97, 128.80, 128.64, 128.33, 128.28, 127.92, 127.36, 127.26, 127.13, 126.96, 126.41, 126.31, 74.13, 71.48, 50.07, 46.15, 29.83, 29.59, 28.92, 21.63.

HRMS (APCI pos.) m/z: [M+Na] calcd. for $C_{16}H_{16}ONa$, 247.10934; found 247.10842.

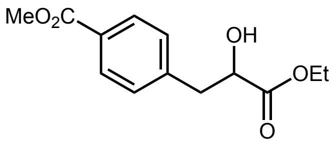

Methyl 4-(3-ethoxy-2-hydroxy-3-oxopropyl)benzoate (47): Prepared according to general procedure A using methyl 4-iodobenzoate (0.5 mmol, 131 mg, 1.0 equiv), Na_2CO_3 (1 mmol, 106 mg, 2.0 equiv), Cl-4CzIPN (0.025 mmol, 26 mg, 5 mol%), $(TMS)_3SiOH$ (0.75 mmol, 0.230 mL, 1.5 equiv), and ethyl acrylate (1.5 mmol, 0.163 mL, 3.0 equiv) in 19:1 MeCN:H_2O (5 mL). The reaction was purified by silica chromatography (0-30% EtOAc/Hex) to afford the title compound as a yellow oil (77.9 mg, 62%).

^{1}H NMR (500 MHz, $CDCl_3$) δ 7.97 (d, *J* = 8.3 Hz, 2H), 7.31 (d, *J* = 8.3 Hz, 2H), 4.46 (dd, *J* = 11.5, 5.6, 1H), 4.22 (q, *J* = 7.1 Hz, 2H), 3.90 (s, 3H), 3.18 (dd, *J* = 13.9, 4.4 Hz, 1H), 3.02 (dd, *J* = 13.9, 6.8 Hz, 1H), 2.82 (d, *J* = 5.7 Hz, 1H), 1.28 (t, *J* = 7.1, 3H).

^{13}C NMR (101 MHz, $CDCl_3$) δ 174.08, 167.15, 142.05, 129.79, 129.74, 128.93, 70.96, 62.12, 52.21, 40.54, 14.34.

HRMS (APCI pos.) m/z: [M+H] calcd. for $C_{13}H_{17}O_5$, 253.10705; found 253.10673.

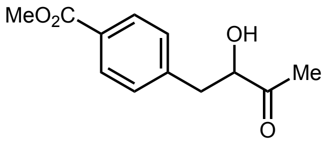

Methyl 4-(2-hydroxy-3-oxobutyl)benzoate (48): Prepared according to general procedure A using methyl 4-iodobenzoate (0.5 mmol, 131 mg, 1.0 equiv), Na_2CO_3 (1 mmol, 106 mg, 2.0 equiv), Cl-4CzIPN (0.025 mmol, 26 mg, 5 mol%), $(TMS)_3SiOH$ (0.75 mmol, 0.230 mL, 1.5 equiv), and but-3-en-2-one (1.5 mmol, 0.123 mL, 3.0 equiv) in 19:1 MeCN:H_2O (5 mL). The reaction was purified by silica chromatography (0-30% EtOAc/Hex) to afford the title compound as a yellow oil (58.0 mg, 52%).

^{1}H NMR (500 MHz, $CDCl_3$) δ 7.98 (d, *J* = 8.4 Hz, 2H), 7.32 (d, *J* = 8.4 Hz, 2H), 4.47 – 4.42 (m, 1H), 3.90 (s, 3H), 3.46 (d, *J* = 4.5 Hz, 1H), 3.21 (dd, *J* = 14.2, 4.4 Hz, 1H), 2.91 (dd, *J* = 14.2, 7.4 Hz, 1H), 2.23 (s, 3H).

^{13}C NMR (101 MHz, $CDCl_3$) δ 208.68, 167.05, 142.02, 129.95, 129.47, 129.03, 77.40, 52.23, 39.91, 25.89.

HRMS (APCI pos.) m/z: [M+H] calcd. for $C_{12}H_{15}O_4$, 253.10705; found 253.10673.

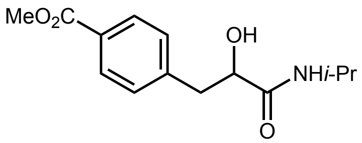

Methyl 4-(2-hydroxy-3-(isopropylamino)-3-oxopropyl)benzoate (49): Prepared according to general procedure A using methyl 4-iodobenzoate (0.5 mmol, 131 mg, 1.0 equiv), Na_2CO_3 (1 mmol, 106 mg, 2.0 equiv), Cl-4CzIPN (0.025 mmol, 26 mg, 5 mol%), $(TMS)_3SiOH$ (0.75 mmol, 0.230 mL, 1.5 equiv), and *N*-isopropylacrylamide (1.5 mmol, 170 mg, 3.0 equiv) in 19:1 MeCN:H_2O (5 mL). The reaction was purified by silica chromatography (0-70% EtOAc/Hex) to afford the title compound as a white solid (68.4 mg, 52%).

^{1}H NMR (600 MHz, $CDCl_3$) δ 7.98 (d, *J* = 8.4 Hz, 2H), 7.32 (d, *J* = 8.4 Hz, 2H), 6.21 (d, *J* = 8.2 Hz, 1H), 4.29 (dt, *J* = 7.9, 4.5 Hz, 1H), 4.09 – 4.01 (m, 1H), 3.90 (s, 3H), 3.24 (dd, *J* = 13.9, 4.2 Hz, 1H), 2.98 (dd, *J* = 13.9, 7.9 Hz, 1H), 2.58 (d, *J* = 5.4 Hz, 1H), 1.12 (d, *J* = 6.5 Hz, 3H), 1.08 (d, *J* = 6.5 Hz, 3H).

^{13}C NMR (101 MHz, $CDCl_3$) δ 171.24, 167.09, 142.53, 130.01, 129.83, 129.03, 72.63, 52.27, 41.28, 41.11, 22.82, 22.77.

HRMS (APCI pos.) m/z: [M+H] calcd. for $C_{14}H_{20}O_4N$, 266.13868; found 266.13861.

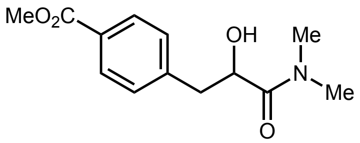

Methyl 4-(3-(dimethylamino)-2-hydroxy-3-oxopropyl)benzoate (50): Prepared according to general procedure A using methyl 4-iodobenzoate (0.5 mmol, 131 mg, 1.0 equiv), Na_2CO_3 (1 mmol, 106 mg, 2.0 equiv), Cl-4CzIPN (0.025

mmol, 26 mg, 5 mol%), $(TMS)_3SiOH$ (0.75 mmol, 0.230 mL, 1.5 equiv), and *N*,*N*-dimethylacrylamide (1.5 mmol, 0.155 mL, 3.0 equiv) in 19:1 $MeCN:H_2O$ (5 mL). The reaction was purified by silica chromatography (0-70% EtOAc/Hex) to afford the title compound as a pale yellow solid (68.0 mg, 54%).

^{1}H NMR (500 MHz, $CDCl_3$) δ 7.97 (d, *J* = 8.3 Hz, 2H), 7.29 (d, *J* = 8.3 Hz,2H), 4.60 (dd, *J* = 7.4, 4.7 Hz, 2H), 3.90 (s, 3H), 2.98 (s, 3H), 2.98 (dd, *J* = 13.8 Hz, 4.8 Hz), 2.89 (dd, *J* = 13.8, 7.4 Hz), 2.86 (s, 3H).

^{13}C NMR (101 MHz, $CDCl_3$) δ 173.40, 167.09, 142.45, 129.86, 129.54, 128.88, 68.69, 52.21, 41.82, 36.51, 36.04.

HRMS (APCI pos.) m/z: [M+H] calcd. for $C_{13}H_{18}O_4N$, 252.12303; found 252.12304.

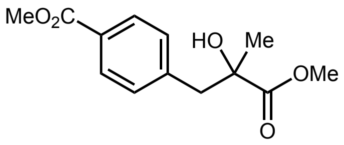

Methyl 4-(2-hydroxy-3-methoxy-2-methyl-3-oxopropyl)benzoate (51): Prepared according to general procedure A using methyl 4-iodobenzoate (0.5 mmol, 131 mg, 1.0 equiv), Na_2CO_3 (1 mmol, 106 mg, 2.0 equiv), Cl-4CzIPN (0.025 mmol, 26 mg, 5 mol%), $(TMS)_3SiOH$ (0.75 mmol, 0.230 mL, 1.5 equiv), and methyl methacrylate (1.5 mmol, 0.160 mL, 3.0 equiv) in 19:1 $MeCN:H_2O$ (5 mL). The reaction was purified by silica chromatography (0-50% EtOAc/Hex) to afford the title compound as a pale yellow solid (118 mg, 94%).

^{1}H NMR (500 MHz, $CDCl_3$) δ 7.94 (d, *J* = 8.2 Hz, 2H), 7.25 (d, *J* = 8.2 Hz, 2H), 3.90 (s, 3H), 3.73 (s, 3H), 3.11 (d, *J* = 13.3 Hz, 1H), 3.10 (s, 1H), 2.97 (d, *J* = 13.3 Hz, 2H), 1.50 (s, 3H).

^{13}C NMR (101 MHz, $CDCl_3$) δ 176.48, 167.15, 141.54, 130.21, 129.59, 128.99, 75.29, 52.87, 52.19, 46.36, 26.17.

HRMS (APCI pos.) m/z: [M+H] calcd. for $C_{13}H_{17}O_5$, 253.10705; found 253.10713.

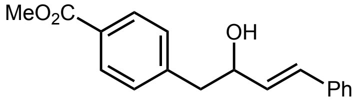

Methyl 4-(2-hydroxy-4-phenylbut-3-en-1-yl)benzoate (52): Prepared according to general procedure A using methyl 4-iodobenzoate (0.5 mmol, 131 mg, 1.0 equiv), Na_2CO_3 (1 mmol, 106 mg, 2.0 equiv), Cl-4CzIPN (0.025 mmol, 26 mg, 5 mol%), $(TMS)_3SiOH$ (0.75 mmol, 0.230 mL, 1.5 equiv), and (*E*)-buta-1,3-dien-1-ylbenzene (1.5 mmol, 195 mg, 3.0 equiv) in 19:1 $MeCN:H_2O$ (5 mL). The reaction was purified by silica chromatography (150 mL 50% DCM/Hex then 10-20% EtOAc/Hex) to afford the title compound as a white solid (82.1 mg, 58%).

^{1}H NMR (400 MHz, $CDCl_3$) δ 7.99 (d, *J* = 8.3 Hz, 2H), 7.39 – 7.20 (m, 6H), 7.27 – 7.22 (m, 1H), 6.58 (d, *J* = 15.9 Hz, 1H), 6.25 (dd, *J* = 15.9, 6.5 Hz, 1H), 4.61 – 4.51 (m, 1H), 3.91 (s, 3H), 3.06 – 2.90 (m, 2H), 1.72 (s, 1H).

^{13}C NMR (101 MHz, $CDCl_3$) δ 167.18, 143.41, 136.63, 131.27, 131.00, 129.93, 129.80, 128.77, 128.75, 127.98, 126.67, 73.48, 52.17, 44.22.

HRMS (APCI pos.) m/z: [M+H] calcd. for $C_{18}H_{19}O_3$, 283.13287; found 283.13305.

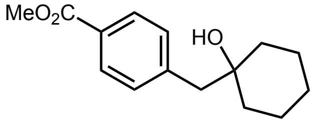

Methyl 4-(3-(dimethylamino)-2-hydroxy-3-oxopropyl)benzoate (53): Prepared according to general procedure A using methyl 4-iodobenzoate (0.5 mmol, 131 mg, 1.0 equiv), Na_2CO_3 (1 mmol, 106 mg, 2.0 equiv), Cl-4CzIPN (0.025 mmol, 26 mg, 5 mol%), $(TMS)_3SiOH$ (0.75 mmol, 0.230 mL, 1.5 equiv), and methylenecyclohexane (1.5 mmol, 0.180 mL, 3.0 equiv) in 19:1 $MeCN:H_2O$ (5 mL). The reaction was purified by silica chromatography (150 mL 50% DCM/Hex then 10-20% EtOAc/Hex) to afford the title compound as a yellow oil (57.8 mg, 47%).

^{1}H NMR (500 MHz, $CDCl_3$) δ 7.97 (d, *J* = 8.1 Hz, 2H), 7.29 (d, *J* = 8.1 Hz, 2H), 3.91 (s, 3H), 2.80 (s, 2H), 1.65 – 1.40 (m, 8H), 1.30 – 1.20 (m, 2H).

^{13}C NMR (101 MHz, $CDCl_3$) δ 167.27, 143.04, 130.78, 129.45, 128.47, 71.50, 52.17, 48.88, 37.54, 25.80, 22.20.

HRMS (APCI pos.) m/z: [M+H] calcd. for $C_{15}H_{21}O_3$, 249.14852; found 249.14845.

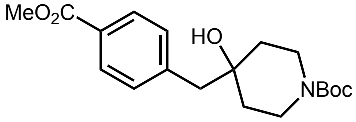

***tert*-butyl 4-hydroxy-4-(4-(methoxycarbonyl)benzyl)piperidine-1-carboxylate (54):** Prepared according to general procedure A using methyl 4-iodobenzoate (0.5 mmol, 131 mg, 1.0 equiv), Na_2CO_3 (1 mmol, 106 mg, 2.0 equiv), Cl-4CzIPN (0.025 mmol, 26 mg, 5 mol%), $(TMS)_3SiOH$ (0.75 mmol, 296 mg, 1.5 equiv), and *tert*-butyl 4-methylenepiperidine-1-carboxylate (1.5 mmol, 195 mg, 3.0 equiv) in 19:1 MeCN:H_2O (5 mL). The reaction was purified by silica chromatography (150 mL 50% DCM/Hex then 10-50% EtOAc/Hex) to afford the title compound as a yellow solid (92.4 mg, 52%).
^{1}H NMR (400 MHz, $CDCl_3$) δ 7.99 (d, *J* = 8.4 Hz, 2H), 7.27 (d, *J* = 8.4 Hz, 2H), 3.91 (s, 3H), 3.85 (d, *J* = 12.7 Hz, 2H), 3.09 (t, *J* = 12.3 Hz, 2H), 2.81 (s, 1H), 1.66 – 1.47 (m, 4H), 1.45 (s, 9H), 1.20 (s, 1H).
^{13}C NMR (101 MHz, $CDCl_3$) δ 167.09, 154.95, 141.75, 130.71, 129.75, 128.99, 79.63, 69.84, 52.22, 49.42, 39.83 36.94, 28.61.
HRMS (ESI neg.) m/z: [M+Cl] calcd. for $C_{19}H_{27}O_5NCl$, 384.15832; found 384.15816.

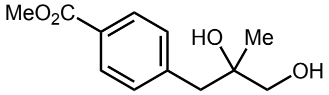

Methyl 4-(2,3-dihydroxy-2-methylpropyl)benzoate (55): Prepared according to general procedure A using methyl 4-iodobenzoate (0.5 mmol, 131 mg, 1.0 equiv), Na_2CO_3 (1 mmol, 106 mg, 2.0 equiv), Cl-4CzIPN (0.025 mmol, 26 mg, 5 mol%), $(TMS)_3SiOH$ (0.75 mmol, 0.230 mL, 1.5 equiv), and 2-methylprop-2-en-1-ol (1.5 mmol, 0.127 mL, 3.0 equiv) in 19:1 MeCN:H_2O (5 mL). The reaction was purified by silica chromatography (0-50% EtOAc/Hex) to afford the title compound as a white solid (37.4 mg, 33%).
^{1}H NMR (500 MHz, $CDCl_3$) δ 7.98 (d, *J* = 8.1 Hz ,2H), 7.32 (d, *J* = 8.1 Hz, 2H), 3.91 (s, 3H), 3.46 (q, *J* = 10.8 Hz, 2H), 2.90 (d, *J* = 13.2 Hz, 1H), 2.84 (d, *J* = 13.2 Hz, 1H), 1.95 (broad s, 1H), 1.13 (s, 3H).
^{13}C NMR (101 MHz, $CDCl_3$) δ 167.22, 142.76, 130.63, 129.65, 128.65, 73.09, 69.38, 52.25, 44.67, 23.71.
HRMS (APCI pos.) m/z: [M+H] calcd. for $C_{12}H_{17}O_4$, 225.11214; found 225.11205.

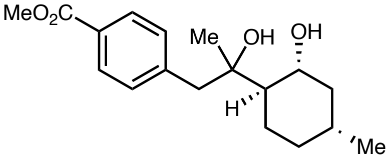

Methyl 4-(2-hydroxy-2-((1*R*,2*R*,4*R*)-2-hydroxy-4-methylcyclohexyl)propyl)benzoate (56): Prepared according to general procedure A using methyl 4-iodobenzoate (0.5 mmol, 131 mg, 1.0 equiv), Na_2CO_3 (1 mmol, 106 mg, 2.0 equiv), Cl-4CzIPN (0.025 mmol, 26 mg, 5 mol%), $(TMS)_3SiOH$ (0.75 mmol, 0.230 mL, 1.5 equiv), and (-) – isopulegol (1.5 mmol, 0.254 mL, 3.0 equiv) in 19:1 MeCN:H_2O (5 mL). The reaction was purified by silica chromatography (0-50% EtOAc/Hex) to afford the title compound as a white solid (98.3 mg, 67%). The compound was characterized as a mixture of diastereomers with the diastereomeric ratio determined by ^{1}H NMR (1.4:1.0 dr, *denotes major diastereomer, #denotes minor diastereomer).
^{1}H NMR (400 MHz, $CDCl_3$) δ 8.99 – 7.95 (m, 2H), 7.34 (t, *J* = 7.8 Hz, 2H), 3.91 (s, 3H#), 3.90 (s, 3H*), 3.87 (td, *J* = 10.5, 4.3 Hz, 1H*), 3.77 (td, *J* = 10.5, 4.3 Hz, 1H#), 3.55 (broad s, 1H), 3.06 (d, *J* = 13.2 Hz, 1H*), 2.85, 2.83 (AB_q, *J* = 13.4 Hz, 2H#), 2.68 (d, *J* = 13.2 Hz, 1H*)#), , 2.02 – 1.89 (m, 1H), 1.88 – 1.77 (m, 1H), 1.76 – 1.63 (m, 1H), 1.60 – 1.52 (m, H*), 1.52 – 1.42 (m, 1H), 1.41 – 1.34 (m, 1H#), 1.21 (s, 3H#), 1.16 – 0.78 (m, 3H), 1.00 (s, 3H), 0.94 (d, *J* = 6.5 Hz, 3H*), 0.90 (d, *J* = 6.5 Hz, 3H#).
^{13}C NMR (101 MHz, $CDCl_3$) δ 167.32, 167.28, 143.54, 142.78, 131.14, 131.13, 129.50, 129.42, 128.64, 128.48, 76.73, 76.56, 72.72, 72.66, 54.67, 52.16, 52.14, 51.27, 47.29, 45.11, 44.87, 42.17, 34.81, 34.45, 31.57, 31.47, 27.24, 27.09, 27.05, 22.88, 22.07, 22.03.
HRMS (APCI pos.) m/z: [M-OH] calcd. for $C_{18}H_{25}O_3$, 289.17982; found 289.17907.

VII. Scaling Experiments:

Table S2. Optimization of reaction set-up on 0.5 mmol scale.

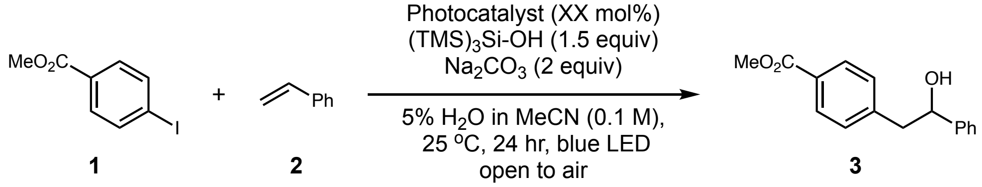

Entry	1	Scale	Septum	Stir bar	Stir rate	Vessel	3
1	0%	0.1 mmol	pierced	10 mm x 3 mm	1000 rpm	8 mL	88%
2	18%	0.2 mmol	pierced	10 mm x 3 mm	1000 rpm	8 mL	59%
3	0%	0.2 mmol	open	10 mm x 3 mm	1000 rpm	8 mL	85%
4	43%	0.5 mmol	open	10 mm x 3 mm	1000 rpm	16 mL	40%
5	0%	0.5 mmol	open	12.7 mm x 3 mm	1400 rpm	20 mL	87%

^{1}H NMR yields with dibromomethane as internal standard

Figure S1. Picture of glassware and stir bars used in scale up reactions.

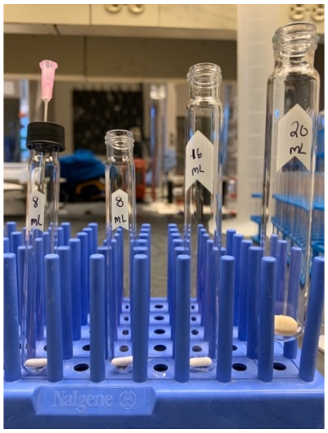

We found the scaling of this reaction to be sensitive to parameters such as O_2 exposure, stir rate, and reaction vessel. Entry 1 in Table S1 shows the optimized reaction conditions on 0.1 mmol scale. Doubling the reaction scale from 0.1 mmol to 0.2 mmol with the exact conditions and reaction set-up resulted in a lower yield of **3** (Entry 2). We postulated on the 0.2 mmol scale that O_2 incorporation into the reaction mixture was not adequate enough with just the pierced septum. Performing the reaction open to air without a septum resulted in a comparable reaction yield the 0.1 mmol scale (Entry 3). Upon scaling to 0.5 mmol, with identical conditions and set-up, we noticed a drastic reduction in starting material consumption and yield (Entry 4). We moved to a large reaction vessel allowing for more headspace and more O_2 exposure, as well as increased stir bar size (12.7 mm length x 8 mm diameter) and stir rate for smooth "vortexing" of the reaction which resulted in yields identical to the 0.1 mmol scale (Entry 5).

Table S3. Optimization of reaction conditions on 2 mmol scale:

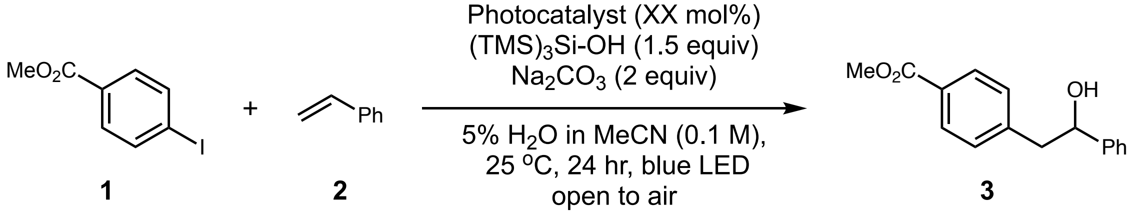

Entry	Scale	Photocatalyst (mol%)	**3**
1	1 mmol	Cl-4CzIPN (5 mol%)	59%
2	2 mmol	Cl-4CzIPN (5 mol%)	46%
3	2 mmol	$[Ir(dFCF_3ppy)_2(5,5'\text{-}dFCF_3bpy)]PF_6$ (1 mol%)	86%

Isolated yields of **3** shown

Figure S2. Picture of set-up for 2 mmol scale reaction:

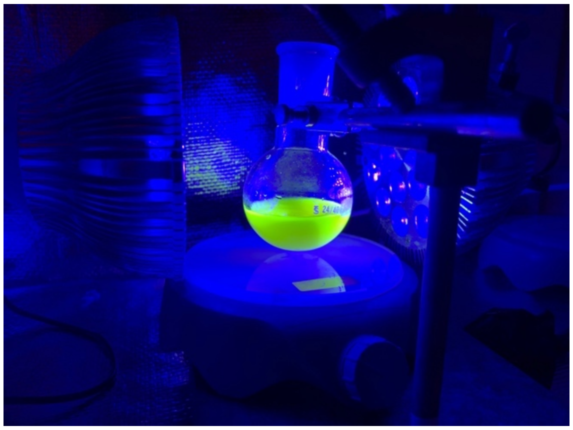

Procedure for hydroxyarylation reaction on 2 mmol scale:

A 100 mL round-bottom 24/40 single-neck flask was equipped with a XX mm x XX mm stir bar and charged with $[Ir(dFCF_3ppy)_2(5,5'\text{-}dFCF_3bpy)]PF_6$ (22.9 mg, 0.02 mmol, 1 mol%), methyl 4-iodobenzoate (524 mg, 2.00 mmol, 1 equiv), Na_2CO_3 (424 mg, 4.00 mmol, 2 equiv), and benchtop MeCN and deionized H_2O (19:1, 20 mL, 0.1 M). Styrene (0.687 mL, 6.00 mmol, 3 equiv) and $(TMS)_3SiOH$ (0.924 mL, 3.00 mmol, 1.5 equiv). The resulting mixture was stirred at 500 rpm for 24 hours under irradiation of 2x Hydrofarm® PPB1002 PowerPAR LED Bulb-Blue 15W/E27 placed perpindicular to the reaction vessel at approximately 6 cm away. After this time, the crude reaction was filtered through a pad of silica using 30 mL of EtOAc and the filtrate as concentrated *in vacuo*. The reaction was purified by silica chromatography (0-20% EtOAc/Hex) to afford **3** (446 mg, 86% yield).

VIII. Preparation of Products from Product Diversification Figure:

Kinetic Resolution of 3:

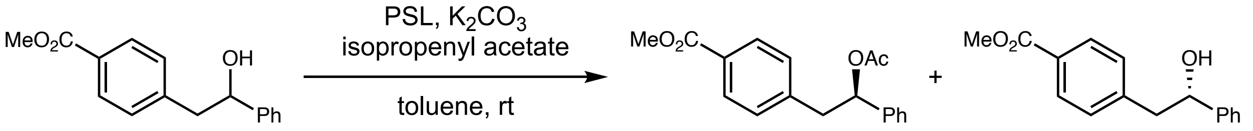

3 **57, 40% yield 96% ee** **58, 60% yield 62% ee**

The following procedure was modified from a previously reported procedure by Park et al.[24] An oven-dried 8 mL screw-top test tube equipped with a stir bar was charged with methyl 4-(2-hydroxy-2-phenylethyl)benzoate (76.9 mg, 0.300 mmol, 1 equiv), isopropenyl acetate (98 µL, 0.900 mmol, 3 equiv), K_2CO_3 (62.2 mg, .450 mmol, 1.5 equiv), and toluene (1 mL, 0.3 M). To this was added PSL (30 mg, 50 wt%) under an N_2 atmosphere. This mixture was stirred at room temperature for 12 hours. After this time, the reaction was diluted with EtOAc (10 mL), filtered through a pad of celite to remove the enzyme and base, and concentrated *in vacuo*. The crude reaction was purified by silica chromatography (0-10% EtOAc/Hex) to afford **methyl (*R*)-4-(2-acetoxy-2-phenylethyl)benzoate** (**57**, 35.4 mg, 40%, 96% e.e.) as a white solid and **methyl (*S*)-4-(2-hydroxy-2-phenylethyl)benzoate** (**58**, 46.3 mg, 60%, 62% ee) as a white solid. The spectral properties of **58** were identical to those of **3**.

methyl (*R*)-4-(2-acetoxy-2-phenylethyl)benzoate (57)

^{1}H NMR (400 MHz, $CDCl_3$) δ 7.91 (d, J = 8.3 Hz, 2H), 7.36 – 7.21 (m, 5H), 7.16 (d, J = 8.3 Hz, 2H), 5.95 (dd, J = 7.8, 6.1 Hz, 2H), 3.90 (s, 3H), 3.25 (dd, J = 13.7, 7.9 Hz, 1H), 3.10 (dd, J = 13.7, 6.1 Hz, 2H), 2.02 (s, 3H).

^{13}C NMR (101 MHz, $CDCl_3$) δ 170.18, 167.18, 142.50, 139.69, 129.72, 129.69, 128.67, 128.60, 128.30, 126.67, 76.28, 52.21, 43.06, 21.28.

HRMS (ESI pos.) m/z: [M+Na] calcd. for $C_{18}H_{18}O_4Na$, 321.10973; found 321.11004.

High pressure liquid chromatography (HPLC) analysis of **57** was performed on an Agilent 1260 Infinity II series HPLC utilizing CHIRALPAK® IH 4.6 x 150 mm analytical column using 5% 2-propanol in hexanes, 1.0 mL/min.

(±)-methyl (*R*)-4-(2-acetoxy-2-phenylethyl)benzoate:

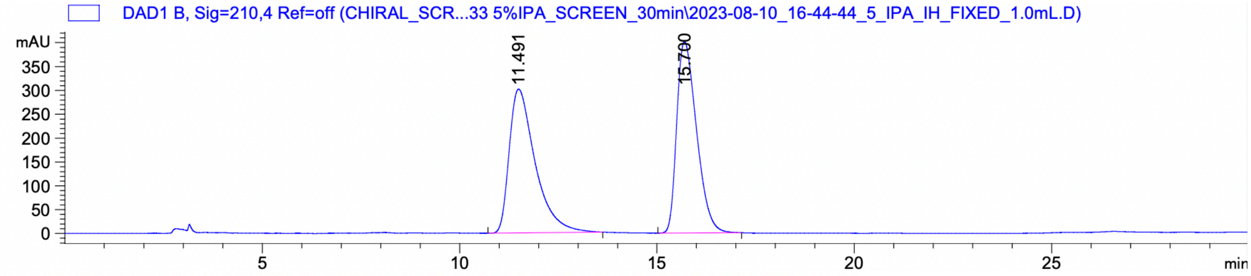

Signal 2: DAD1 B, Sig=210,4 Ref=off

Peak #	RetTime [min]	Type	Width [min]	Area [mAU*s]	Height [mAU]	Area %
1	11.491	BV R	0.5379	1.37848e4	301.79742	49.6535
2	15.700	VV R	0.4220	1.39772e4	401.18539	50.3465
Totals :				2.77619e4	702.98282	

methyl (*R*)-4-(2-acetoxy-2-phenylethyl)benzoate (57):

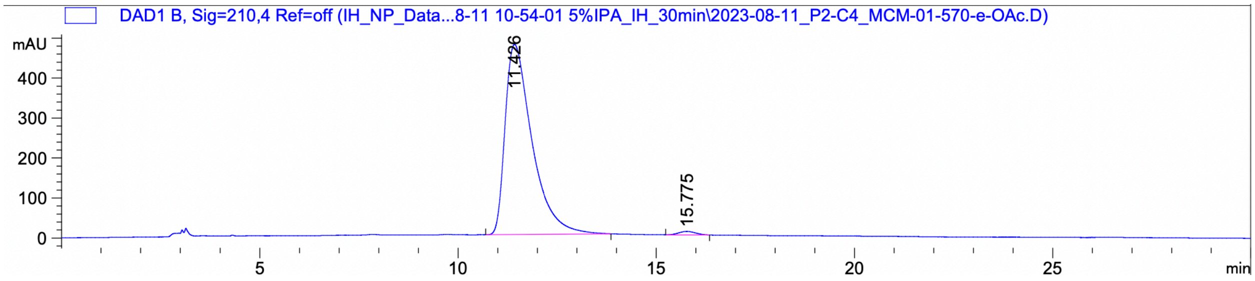

Signal 2: DAD1 B, Sig=210,4 Ref=off

Peak #	RetTime [min]	Type	Width [min]	Area [mAU*s]	Height [mAU]	Area %
1	11.426	BB	0.5553	2.25002e4	475.74182	98.8754
2	15.775	VB R	0.3425	255.90735	8.79478	1.1246
Totals :				2.27561e4	484.53660	

HPLC analysis of **58** was performed on an Agilent 1260 Infinity II series HPLC using CHIRALPAK® IK 4.6 x 150 mm analytical column using 10% 2-propanol in hexanes, 1.0 mL/min.

(±)-Methyl 4-(2-hydroxy-2-phenylethyl)benzoate (3):

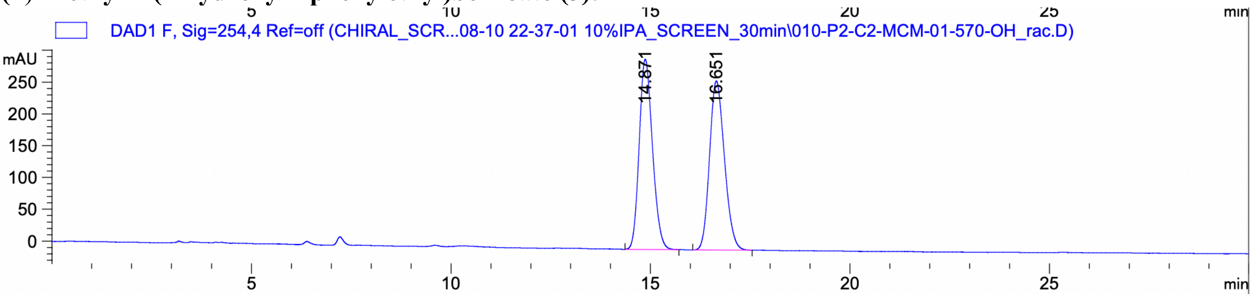

Signal 2: DAD1 B, Sig=210,4 Ref=off

Peak #	RetTime [min]	Type	Width [min]	Area [mAU*s]	Height [mAU]	Area %
1	14.872	VB R	0.3065	1.78815e4	758.56970	50.0954
2	16.654	VV R	0.3081	1.78134e4	678.89563	49.9046
Totals :				3.56949e4	1437.46533	

Methyl (*S*)-4-(2-hydroxy-2-phenylethyl) (58):

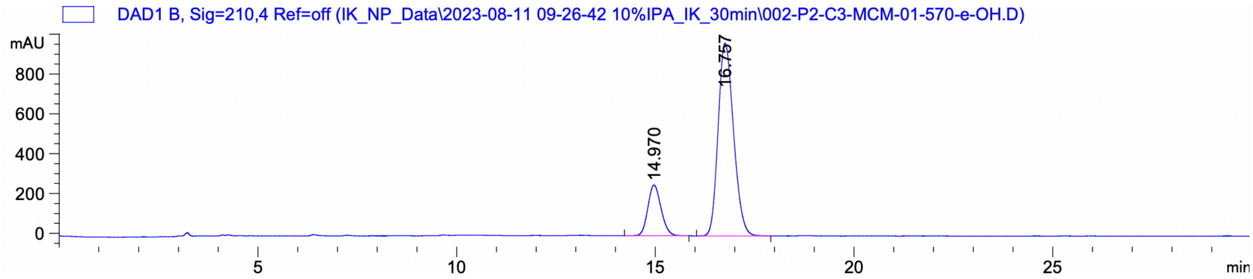

Signal 2: DAD1 B, Sig=210,4 Ref=off

Peak #	RetTime [min]	Type	Width [min]	Area [mAU*s]	Height [mAU]	Area %
1	14.970	VV R	0.3017	5894.60010	254.99280	18.4511
2	16.757	VV R	0.3202	2.60526e4	967.27197	81.5489

Totals : 3.19472e4 1222.26477

Deoxygenative Alkylation of 54:

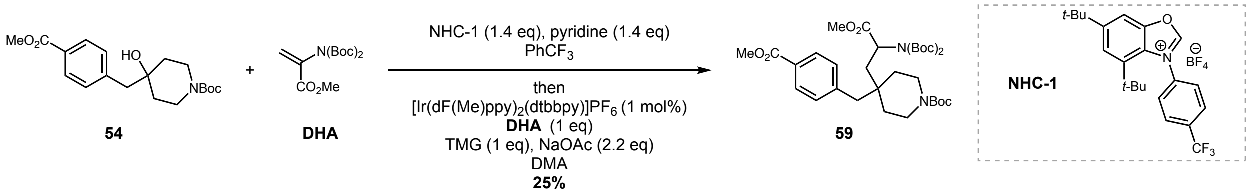

***tert*-butyl 4-(2-(bis(*tert*-butoxycarbonyl)amino)-3-methoxy-3-oxopropyl)-4-(4-(methoxycarbonyl)benzyl)piperidine-1-carboxylate (59):** The following procedure was modified from a previously reported procedure by MacMillan et al.[25] An oven-dried 20 mL screw-top test tube equipped with a stir bar was charged with NHC-1 (139 mg, 0.350 mmol, 1.4 equiv), **54** (122 mg, 0.350 mmol, 1.4 equiv). The tube was sealed with a PTFE/silicon septum, placed under an N_2 atmosphere, and anhydrous $PhCF_3$ (2.5 mL) was added. The reaction mixture was cooled to -25 °C. Pyridine (28.3 µL, 0.350 mmol, 1.4 equiv) was added under an N_2 atmosphere and the reaction was warmed to room temperature while stirring for 2 hours. After this time, [Ir(F(Me)ppy)2(dtbbpy)](PF6) (2.5 mg, .0025 mmol, 1 mol%), sodium acetate (45.1 mg, 0.550 mmol, 2.2 equiv, methyl 2-(bis(*tert*-butoxycarbonyl)amino)acrylate (75.3 mg, 0.250 mmol, 1 equiv), and anhydrous dimethylacetamide (5mL) were added to the suspension. The reaction was degassed by sparging for 15 minutes with N_2. Following sparging, 1,1,3,3-tetramethylguanidine (31.4 µL, 0.250 mmol, 1 equiv) was added and the reaction was irradiated in the integrated photoreactor for 2 hours (100% intensity, 6800 rpm fans, 1000 rpm stirring). After this time, the reaction mixture was diluted with EtOAc (10 mL), washed 3 x with 1 M LiCl (10 mL), 3 x with brine (10 mL), dried over Na_2SO_4, and concentrated *in vacuo*. The reaction was purified by silica gel chromatography (0-30% EtOAc/Hex) to give the title compound as a clear oil (40.2 mg, 25%).
^{1}H NMR (400 MHz, $CDCl_3$) δ 7.92 (d, *J* = 8.2 Hz, 2H), 7.15 (d, *J* = 8.4 Hz, 2H), 5.09 (dd, *J* = 7.2, 2.9 Hz, 1H), 3.89 (s, 3H), 3.71 (s, 3H), broad s (3.54, 2H), 3.36 – 3.19 (m, 2H), 2.73 (s, 2H), 2.41 (dd, *J* = 15.7, 2.9 Hz, 1H), 1.84 (dd, *J* = 15.7, 7.2 Hz, 1H), 1.48 (s, 18H), 1.43 – 1.35 (m, 4H), 1.40 (s, 9H).
^{13}C NMR (101 MHz, $CDCl_3$) δ 171.92, 167.15, 154.97, 152.29, 143.10, 131.00, 129.33, 128.33, 83.65, 79.52, 54.59, 52.73, 52.15, 43.68, 39.08, 36.39, 35.65, 33.62, 28.51, 28.12.
HRMS (ESI pos.) m/z: [M+Na] calcd. for $C_{33}H_{50}O_{10}N_2Na$, 657.33577; found 657.336.

C–H annulation of 54:

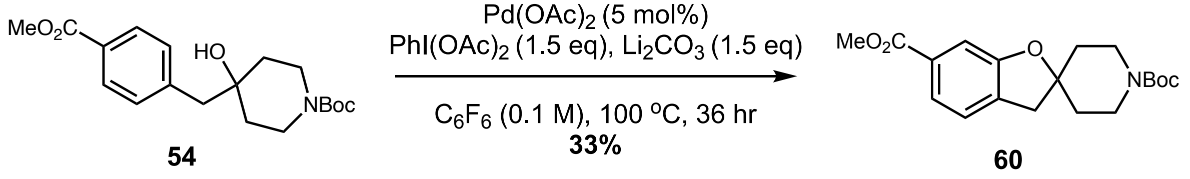

1'-(*tert*-butyl) 6-methyl 3*H*-spiro[benzofuran-2,4'-piperidine]-1',6-dicarboxylate (60): The following procedure was modified from a previously reported procedure by Yu et al.[26] A 20 mL high-pressure tube was equipped with a stir bar and charged with **54** (69.9 mg, 0.200 mmol, 1.0 equiv), $Pd(OAc)_2$ (2.3 mg, 0.01 mmol, 5 mol%), Li_2CO_3 (22.2 mg, 0.300 mmol, 1.5 equiv), $PhI(OAc)_2$ (96.6 mg, 0.300 mmo, 1.5 equiv) under air. The tube was sealed and the reaction mixture was stirred at 100 °C for 36 hours. After this time, the reaction was cooled to room temperature and

diluted with Et_2O (10 mL), filtered through celite, and concentrated *in vacuo*. The reaction was purified by silica gel chromatography (0-10% EtOAc/Hex) to afford the title compound as a white solid.
^{1}H NMR (400 MHz, $CDCl_3$) δ 7.56 (dd, *J* = 7.7, 1.5 Hz, 1H), 7.40 (d, *J* = 1.4 Hz, 1H), 7.19 (d, *J* = 7.7 Hz, 1H), 3.88 (s, 3H), 3.76 (broad s, 2H), 3.45 – 3.34 (m, 2H), 3.02 (s, 2H), 1.95 – 1.85 (m, 2H), 1.70 (ddd, *J* = 14.4, 10.5, 4.6 Hz, 2H), 1.47 (s, 9H).
^{13}C NMR (151 MHz, $CDCl_3$) δ 167.12, 158.78, 154.91, 132.00, 130.65, 125.07, 122.41, 110.74, 86.56, 79.81, 52.22, 41.51, 40.7, 36.54, 28.59.
HRMS (ESI pos.) m/z: [M+Na] calcd. for $C_{19}H_{25}O_5NNa$, 370.16249; found 370.1631.

C–H amination of 54:

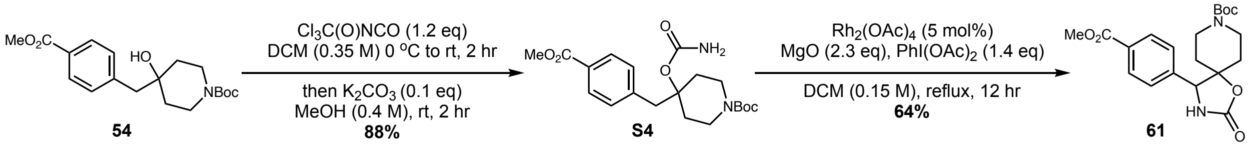

***tert*-butyl 4-(carbamoyloxy)-4-(4-(methoxycarbonyl)benzyl)piperidine-1-carboxylate (S4):** The following procedure was modified from a previously reported procedure by Du Bois et al.[27] An oven-dried 10 mL round-bottom flask equipped with a stir bar was charged with **54** (200 mg, 0.805 mmol, 1 equiv) and the flask was sealed with a rubber septum. DCM (2.3 mL, 0.35 M) was added to the flask under an N_2 atmosphere, and the reaction was cooled to 0 °C. Once cooled, tricholoracetyl isocyanate (0.118 mL, 0.966 mmol, 1.2 equiv) was added to the reaction dropwise. The reaction was warmed to room temperature and stirred until TLC indicated full consumption of **54** (2 hours). After this time, the contents were evaporated *in vacuo*. To the concentrated residue was added K_2CO_3 (11.1 mg, 0.080 mmol, 0.1 equiv) and MeOH (2 mL, 0.4 M) and the reaction was stirred under air at room temperature for 2 hours. After this time, the reaction was diluted with DCM (25 mL) and poured into a separatory funnel containing sat. NH_4Cl (50 mL). The organic phase was collected, and the aqueous phase was washed twice more with 25 mL of DCM. The combined organic extracts were wash with brine (50 mL), dried over Na_2SO_4, and concentrated *in vacuo*. The reaction was purified by dissolving the crude reaction in a minimal amount of MeOH (~500 µL) and adding hexanes (~10 mL) until the solution became cloudy. This solution was heated to boiling where the solution became clear. The solution was cooled to room temperature and place in the freezer overnight to collect the title compound as white crystals that were used in the next step (206 mg, 88%).
^{1}H NMR (400 MHz, $CDCl_3$) δ 7.96 (d, *J* = 8.3 Hz, 2H), 7.24 (d, *J* = 8.3 Hz, 2H), 4.59 (broad s, 2H), 4.00 – 3.80 (m, 2H 3.90 (s, 3H), 3.30 (s, 2H), 3.05 – 2.85 (m, 2H), 2.21 (d, *J* = 13.7 Hz, 2H), 1.58 – 1.47 (m, 2H), 1.44 (s, 9H).
^{13}C NMR (101 MHz, $CDCl_3$) δ 167.16, 155.78, 154.93, 141.77, 130.69, 129.53, 128.74, 80.22, 79.77, 52.21, 43.68, 39.50, 34.34, 28.56.
HRMS (ESI pos.) m/z: [M+Na] calcd. for $C_{20}H_{28}O_6N_2Na$, 415.18396; found 415.18412.

***tert*-butyl 4-(4-(methoxycarbonyl)phenyl)-2-oxo-1-oxa-3,8-diazaspiro[4.5]decane-8-carboxylate (61):** The following procedure was modified from a previously reported procedure by Du Bois et al.[27] An oven-dried 10 mL round-bottom flask equipped with a stir bar was charged with **S1** (150 mg, 0.515 mmol, 1 equiv) and equipped with a reflux condenser. To this flask was added DCM (3.4 mL, 0.15 M) under N_2. Under a stream of N_2, MgO (47.7 mg, 1.18 mmol, 2.3 equiv), $PhI(OAc)_2$ (250 mg, 0.777 mmol, 1.5 equiv), and $Rh_2(OAc)_4$ (11.4 mg, 0.026 mmol, 5 mol%) were added successively. The reaction was heated to 40 °C and stirred for 12 hours. After this time, the reaction was cooled to room temperature and filtered through a pad of celite and the filtrate was concentrated *in vacuo*. The reaction was purified by silica gel chromatography (0-3% MeOH/DCM) to afford the title product as a clear oil.
^{1}H NMR (400 MHz, $CDCl_3$) δ 8.07 (d, *J* = 8.3 Hz, 1H), 7.34 (d, *J* = 8.3 Hz, 1H), 5.54 (broad s, 1H), 4.64 (s, 1H), 4.07 (broad s, 1H), 3.93 (s, 3H), 3.84 (broad s, 1H), 3.16 (t, *J* = 12.5 Hz, 1H), 2.97 (t, *J* = 12.5 Hz, 1H), 2.12 (dd, *J* = 13.7, 2.7 Hz, 1H), 1.83 (td, *J* = 12.9, 5.0 Hz, 1H), 1.41 (s, 9H), 1.38 – 1.29 (m, 1H), 0.91 (broad s, 1H).
^{13}C NMR (151 MHz, $CDCl_3$) δ 166.47, 157.89, 154.54, 141.01, 131.15, 130.36, 126.95, 83.66, 80.08, 65.52, 52.50, 36.95, 32.80, 28.49.
HRMS (ESI pos.) m/z: [M+Na] calcd. for $C_{20}H_{26}O_6N_2Na$, 413.1696; found 413.16885.

IX. References

(1) Wiles, R. J.; Phelan, J. P.; Molander, G. A. Metal-free defluorinative arylation of trifluoromethyl alkenes via photoredox catalysis. *Chem. Comm.* **2019**, *55* (53), 7599-7602.
(2) Le, C.; Chen, T. Q.; Liang, T.; Zhang, P.; MacMillan, D. W. C. A radical approach to the copper oxidative addition problem: Trifluoromethylation of bromoarenes. *Science* **2018**, *360* (6392), 1010-1014.
(3) Kustiana, B. A.; Melen, R. L.; Morrill, L. C. One-Pot Synthesis of Styrene Derivatives from Allyl Silanes via B(C6F5)3-Catalyzed Isomerization–Hiyama Coupling. *Org. Lett.* **2022**, *24* (47), 8694-8697.
(4) Lee, W.-C. C.; Wang, J.; Zhu, Y.; Zhang, X. P. Asymmetric Radical Bicyclization for Stereoselective Construction of Tricyclic Chromanones and Chromanes with Fused Cyclopropanes. *J. Am. Chem. Soc.* **2023**, *145* (21), 11622-11632.
(5) Sharland, J. C.; Wei, B.; Hardee, D. J.; Hodges, T. R.; Gong, W.; Voight, E. A.; Davies, H. M. L. Asymmetric synthesis of pharmaceutically relevant 1-aryl-2-heteroaryl- and 1,2-diheteroarylcyclopropane-1-carboxylates. *Chem. Sci.* **2021**, *12* (33), 11181-11190.
(6) Spitz, C.; Matteudi, M.; Tintori, G.; Broggi, J.; Terme, T.; Vanelle, P. Metal-Free Addition of Benzyl Halides to Aldehydes Using Super Electron Donors: Access to 3,4-Dihydroisocoumarins and 1,2-Diarylethanols. *J. Org. Chem.* **2020**, *85* (23), 15736-15742.
(7) Wang, H.; Wang, Z.; Zhao, G.; Ramadoss, V.; Tian, L.; Wang, Y. Electrochemical Deoxygenative Barbier-Type Reaction. *Org. Lett.* **2022**, *24* (20), 3668-3673.
(8) Kim, S.-H.; Rieke, R. D. Benzylic Manganese Halides, Sulfonates, and Phosphates: Preparation, Coupling Reactions, and Applications in Organic Synthesis. *J. Org. Chem.* **2000**, *65* (8), 2322-2330.
(9) Wang, J.; Xue, L.; Hong, M.; Ni, B.; Niu, T. Heterogeneous visible-light-induced Meerwein hydration reaction of alkenes in water using mpg-C3N4 as a recyclable photocatalyst. *Green Chem.* **2020**, *22* (2), 411-416,
(10) Potrząsaj, A.; Musiejuk, M.; Chaładaj, W.; Giedyk, M.; Gryko, D. Cobalt Catalyst Determines Regioselectivity in Ring Opening of Epoxides with Aryl Halides. *J. Am. Chem. Soc.* **2021**, *143* (25), 9368-9376.
(11) Lu, X.-Y.; Yang, C.-T.; Liu, J.-H.; Zhang, Z.-Q.; Lu, X.; Lou, X.; Xiao, B.; Fu, Y. Cu-Catalyzed cross-coupling reactions of epoxides with organoboron compounds. *Chem. Comm.* **2015**, *51* (12), 2388-2391.
(12) Dilek, Ö.; Tezeren, M. A.; Tilki, T.; Ertürk, E. Chiral 2-(2-hydroxyaryl)alcohols (HAROLs) with a 1,4-diol scaffold as a new family of ligands and organocatalysts. *Tetrahedron* **2018**, *74* (2), 268-286.
(13) Nielsen, D. K.; Doyle, A. G. Nickel-Catalyzed Cross-Coupling of Styrenyl Epoxides with Boronic Acids. *Angew. Chem. Int. Ed.* **2011**, *50* (27), 6056-6059.
(14) Barrios-Rivera, J.; Xu, Y.; Clarkson, G. J.; Wills, M. Asymmetric transfer hydrogenation of heterocycle-containing acetophenone derivatives using N-functionalised [(benzene)Ru(II)(TsDPEN)] complexes. *Tetrahedron* **2022**, *103*, 132562.
(15) Houminer, Y. Studies on the thermolysis of 2-(2-hydroxy-2-arylethyl)pyrazines. An example of a retro-ene-type reaction. *J. Org. Chem.* **1980**, *45* (6), 999-1003.
(16) Hollerbach, M. R.; Barker, T. J. Chemoselective Benzylation of Aldehydes Using Lewis Base Activated Boronate Nucleophiles. *Organometallics* **2018**, *37* (9), 1425-1427.
(17) Alam, R.; Molander, G. A. Direct Synthesis of Secondary Benzylic Alcohols Enabled by Photoredox/Ni Dual-Catalyzed Cross-Coupling. *J. Org. Chem.* **2017**, *82* (24), 13728-13734.
(18) Makarov, A. S.; Kekhvaeva, A. E.; Chalikidi, P. N.; Abaev, V. T.; Trushkov, I. V.; Uchuskin, M. G. A Simple Synthesis of Densely Substituted Benzofurans by Domino Reaction of 2-Hydroxybenzyl Alcohols with 2-Substituted Furans. *Synthesis* **2019**, *51* (19), 3747-3757.
(19) Lebedev, Y.; Polishchuk, I.; Maity, B.; Dinis Veloso Guerreiro, M.; Cavallo, L.; Rueping, M. Asymmetric Hydroboration of Heteroaryl Ketones by Aluminum Catalysis. *J. Am. Chem. Soc.* **2019**, *141* (49), 19415-19423.
(20) Das, M.; O'Shea, D. F. Synthesis and application of benzyl-TMS derivatives as bench stable benzyl anion equivalents. *Tetrahedron* **2013**, *69* (31), 6448-6460.
(21) Li, C.-C.; Dai, X.-J.; Wang, H.; Zhu, D.; Gao, J.; Li, C.-J. Iron-Catalyzed Nucleophilic Addition Reaction of Organic Carbanion Equivalents via Hydrazones. *Org. Lett.* **2018**, *20* (13), 3801-3805.
(22) Hatano, M.; Suzuki, S.; Ishihara, K. Highly Efficient Alkylation to Ketones and Aldimines with Grignard Reagents Catalyzed by Zinc(II) Chloride. *J. Am. Chem. Soc.* **2006**, *128* (31), 9998-9999.
(23) Peach, P.; Cross, D. J.; Kenny, J. A.; Mann, I.; Houson, I.; Campbell, L.; Walsgrove, T.; Wills, M. Asymmetric transfer hydrogenation of α,β-unsaturated, α-tosyloxy and α-substituted ketones. *Tetrahedron* **2006**, *62* (8), 1864-1876.
(24) Kim, M.-J.; Choi, Y. K.; Kim, S.; Kim, D.; Han, K.; Ko, S.-B.; Park, J. Highly Enantioselective Dynamic Kinetic Resolution of 1,2−Diarylethanols by a Lipase−Ruthenium Couple. *Org. Lett.* **2008**, *10* (6), 1295-1298.

(25) Wang, J. Z.; Sakai, H. A.; MacMillan, D. W. C. Alcohols as Alkylating Agents: Photoredox-Catalyzed Conjugate Alkylation via In Situ Deoxygenation. *Angew. Chem. Int. Ed.* **2022**, *61* (35), e202207150.
(26) Wang, X.; Lu, Y.; Dai, H.-X.; Yu, J.-Q. Pd(II)-Catalyzed Hydroxyl-Directed C−H Activation/C−O Cyclization: Expedient Construction of Dihydrobenzofurans. *J. Am. Chem. Soc.* **2010**, *132* (35), 12203-12205.
(27) Espino, C. G.; Du Bois, J. A Rh-Catalyzed C−H Insertion Reaction for the Oxidative Conversion of Carbamates to Oxazolidinones. *Angew. Chem. Int. Ed* **2001**, *40* (3), 598-600.

X. NMR Spectra

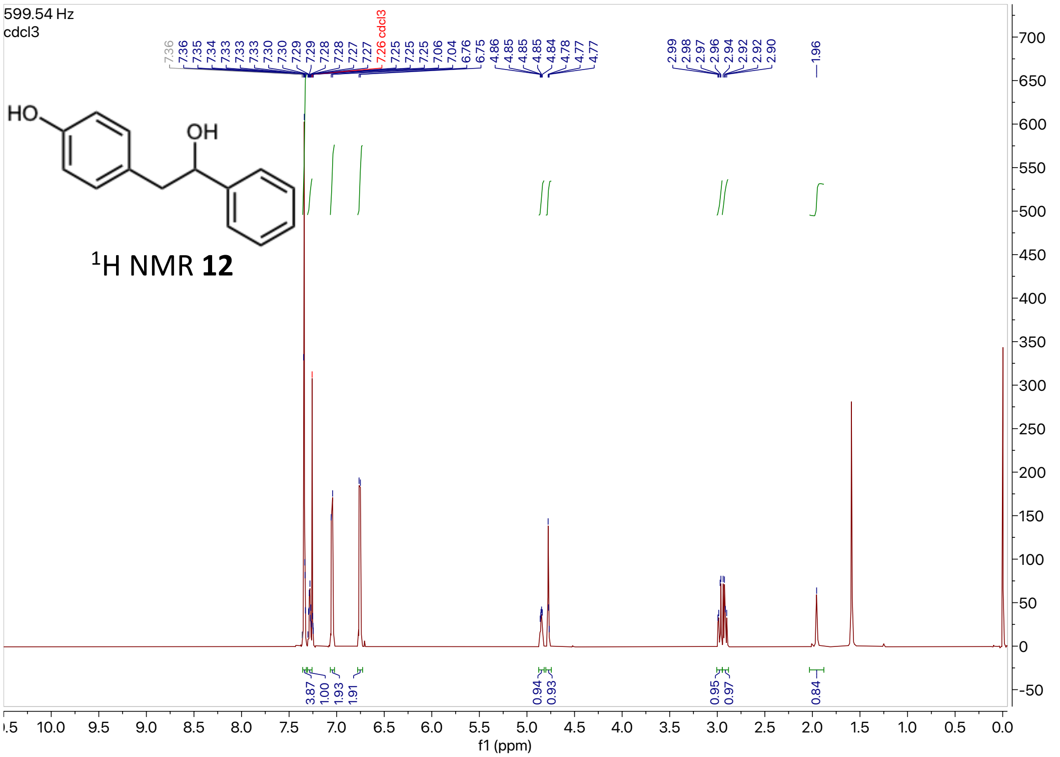

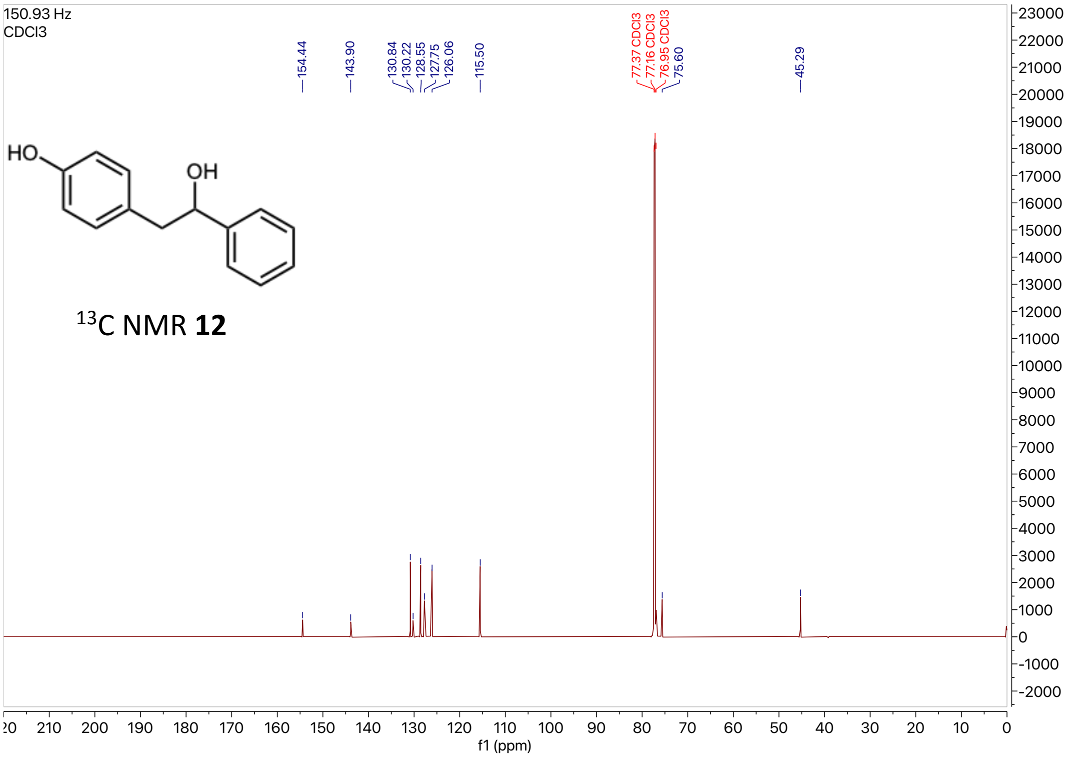

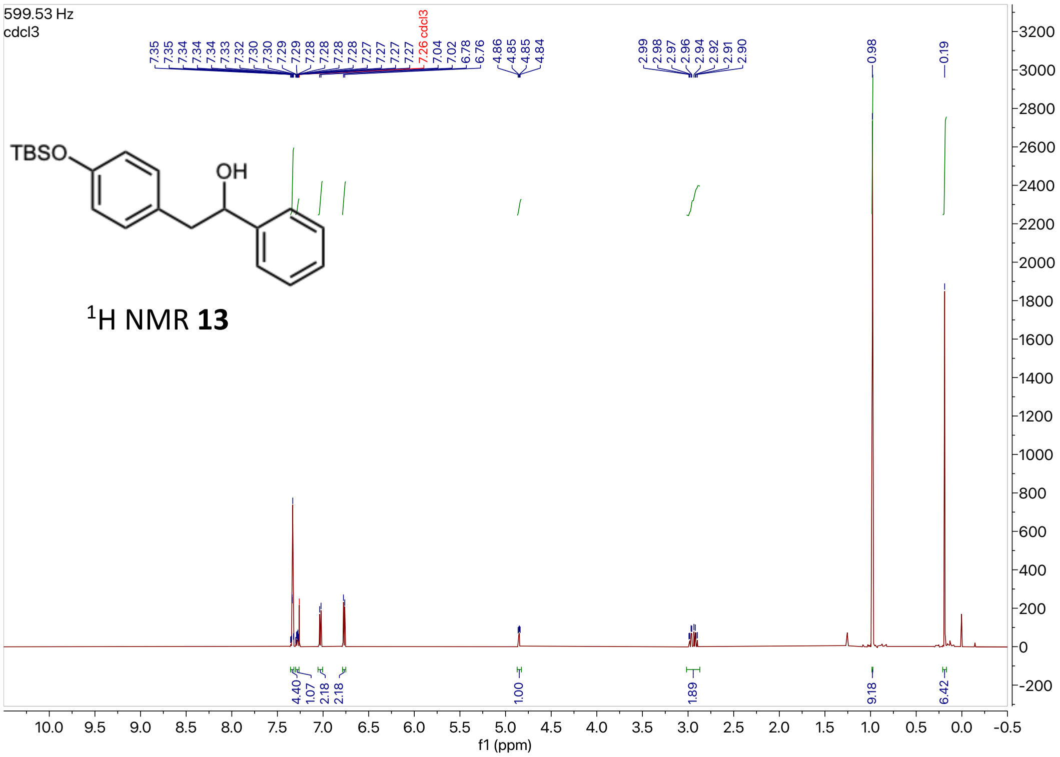
599.53 Hz
cdcl3
7.35
7.35
7.34
7.34
7.34
7.33
7.32
7.30
7.30
7.29
7.29
7.29
7.28
7.28
7.28
7.28
7.27
7.27
7.27
7.27
7.26 cdcl3
7.04
7.02
6.78
6.76
4.86
4.85
4.85
4.84
2.99
2.98
2.97
2.96
2.94
2.92
2.91
2.90
0.98
0.19
TBSO
OH
¹H NMR 13
4.40
1.07
2.18
2.18
1.00
1.89
9.18
6.42
f1 (ppm)

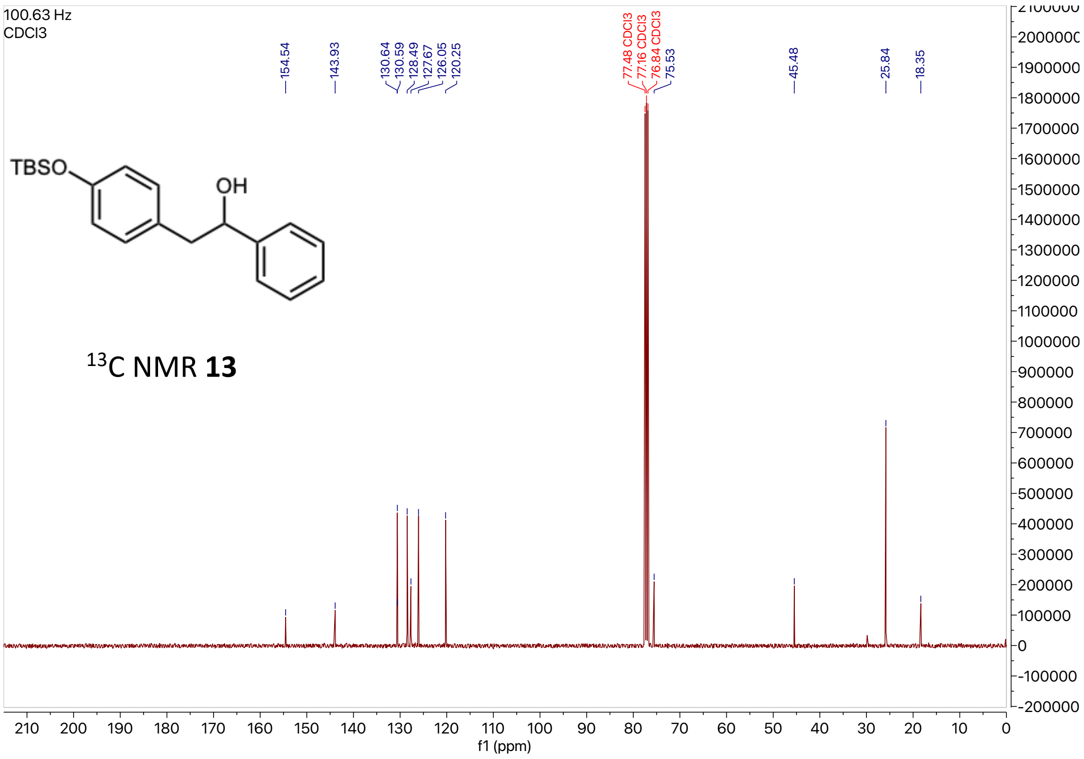
100.63 Hz
CDCl3
154.54
143.93
130.64
130.59
128.49
127.67
126.05
120.25
77.48 CDCl3
77.16 CDCl3
76.84 CDCl3
75.53
45.48
25.84
18.35
TBSO
OH
¹³C NMR 13
f1 (ppm)

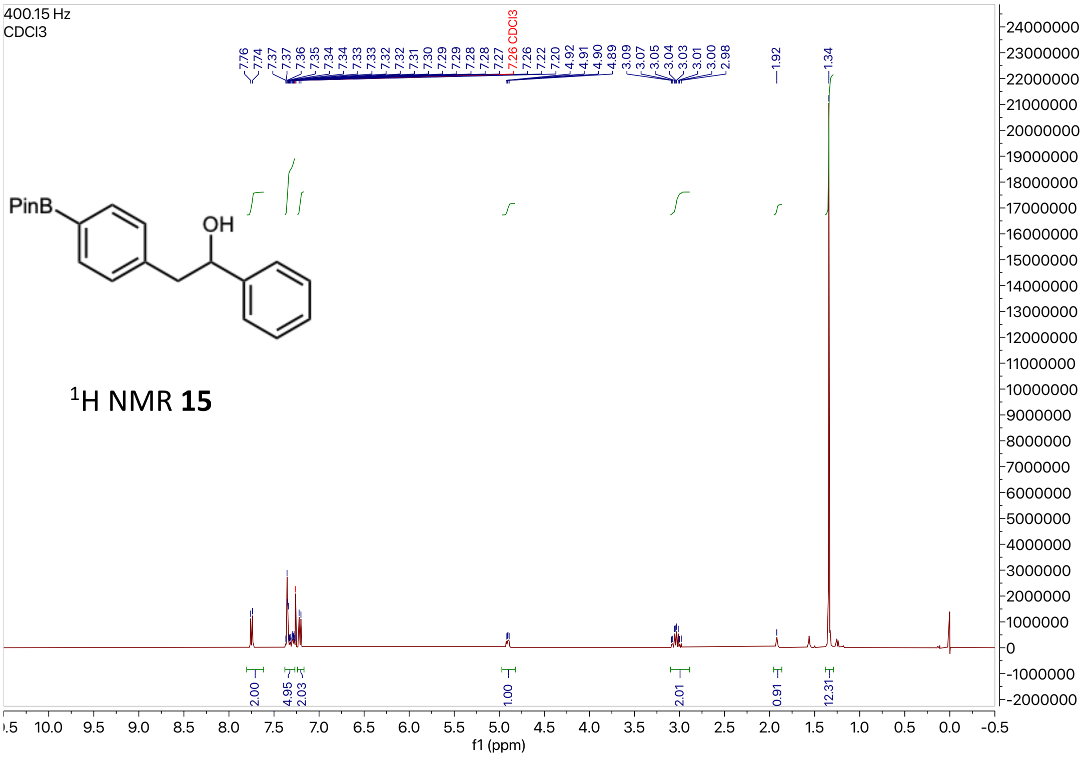

^{1}H NMR **15**

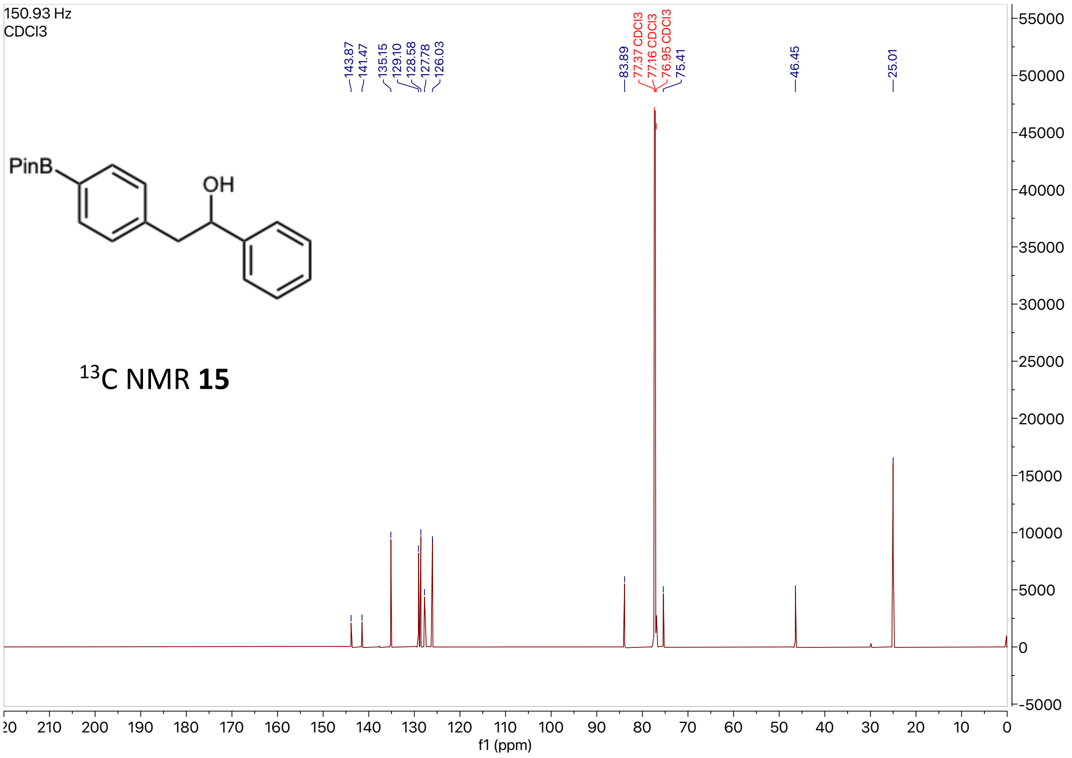

^{13}C NMR **15**

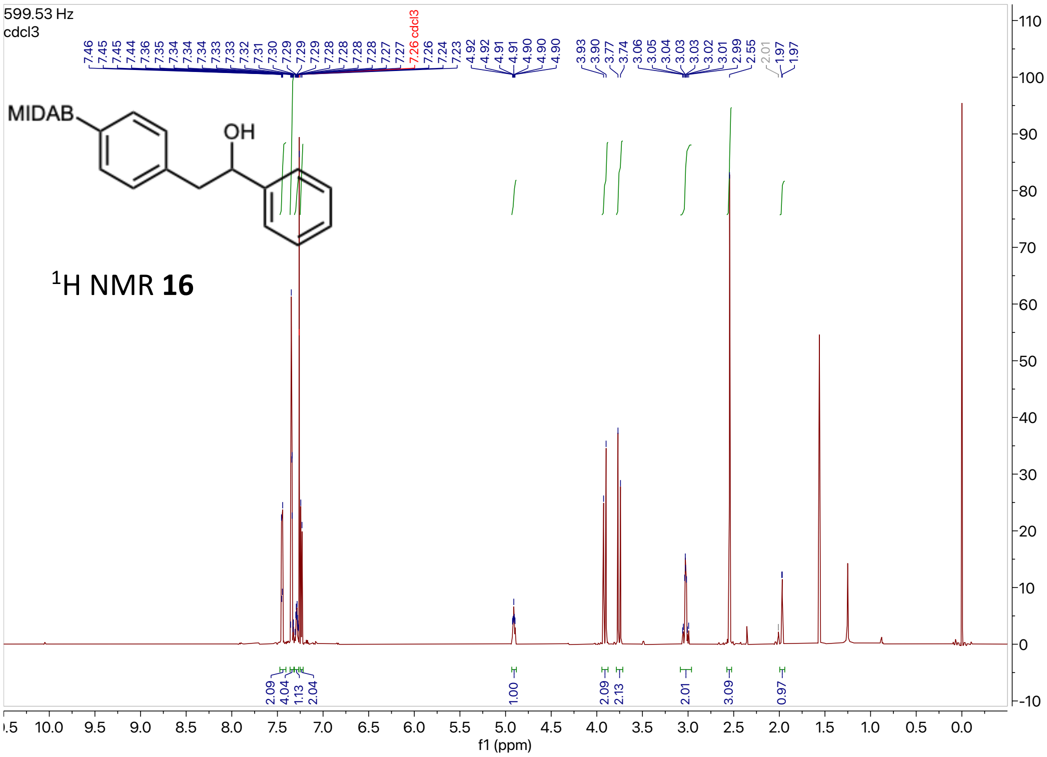
599.53 Hz
cdcl3
MIDAB
OH
1H NMR 16
f1 (ppm)

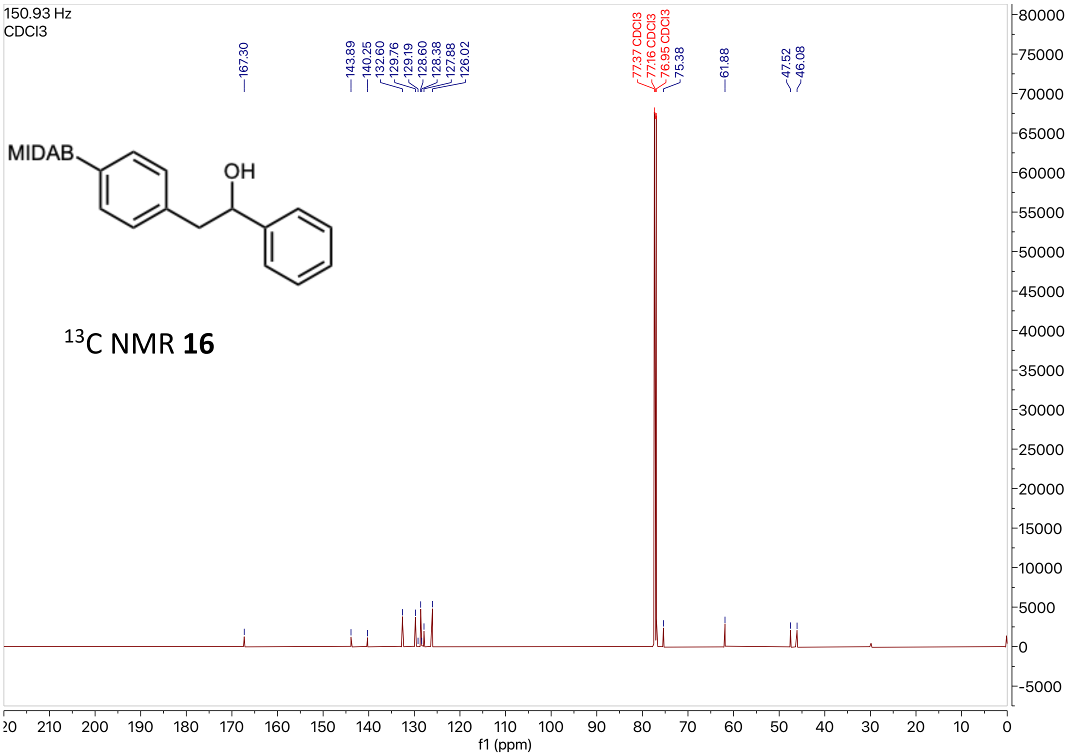
150.93 Hz
CDCl3
MIDAB
OH
13C NMR 16
f1 (ppm)

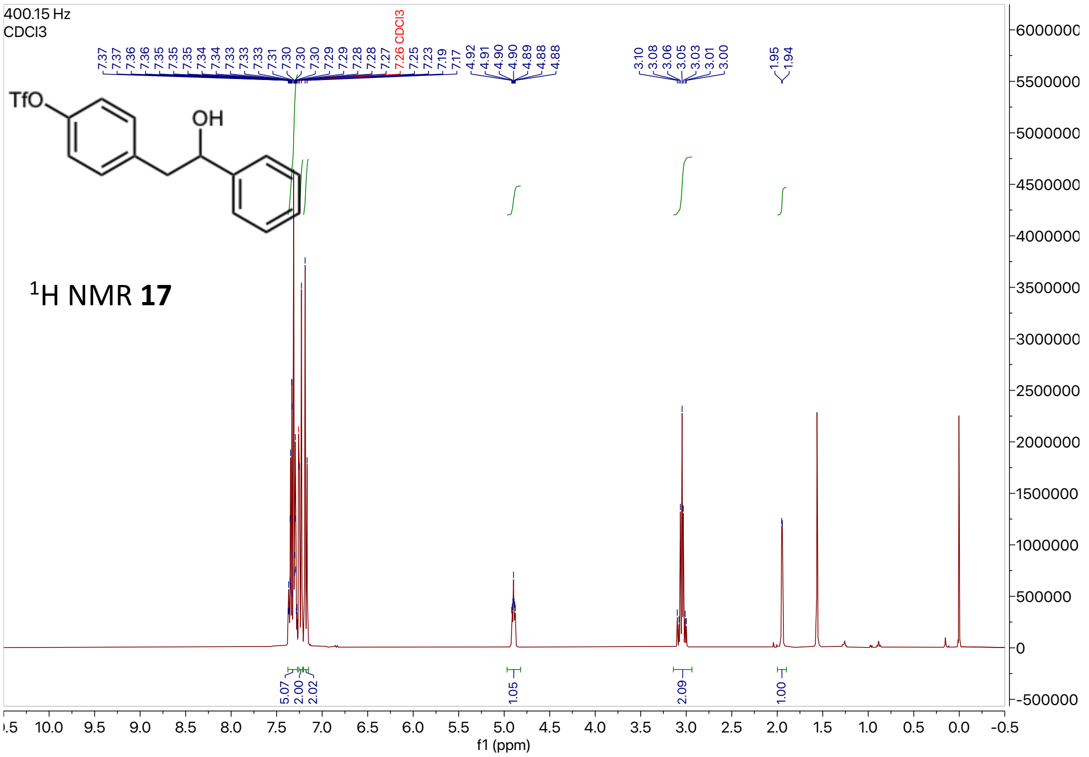
400.15 Hz
CDCl3
7.37 7.37 7.36 7.36 7.35 7.35 7.35 7.34 7.34 7.33 7.33 7.33 7.31 7.30 7.30 7.30 7.29 7.29 7.28 7.28 7.27 7.26 CDCl3 7.25 7.23 7.19 7.17 4.92 4.91 4.90 4.90 4.89 4.88 4.88
3.10 3.08 3.06 3.05 3.03 3.01 3.00
1.95 1.94
TfO
OH
1H NMR 17
5.07 2.00 2.02
1.05
2.09
1.00
f1 (ppm)

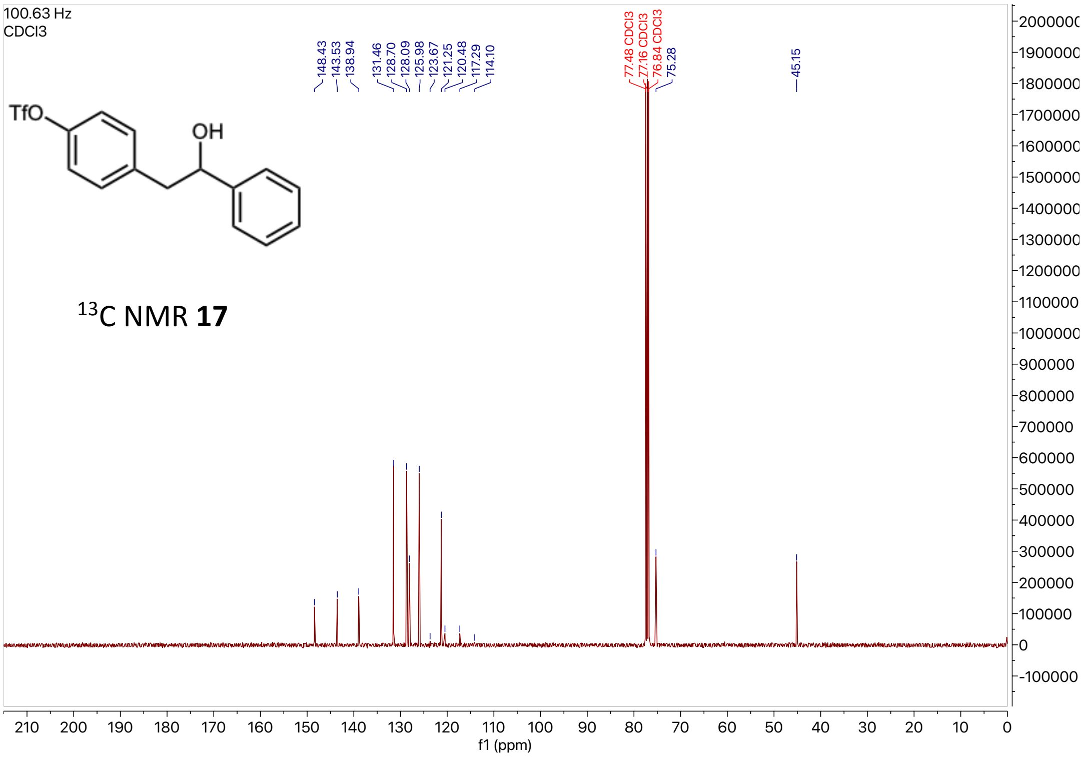
100.63 Hz
CDCl3
148.43 143.53 138.94
131.46 128.70 128.09 125.98 123.67 121.25 120.48 117.29 114.10
77.48 CDCl3 77.16 CDCl3 76.84 CDCl3 75.28
45.15
TfO
OH
13C NMR 17
f1 (ppm)

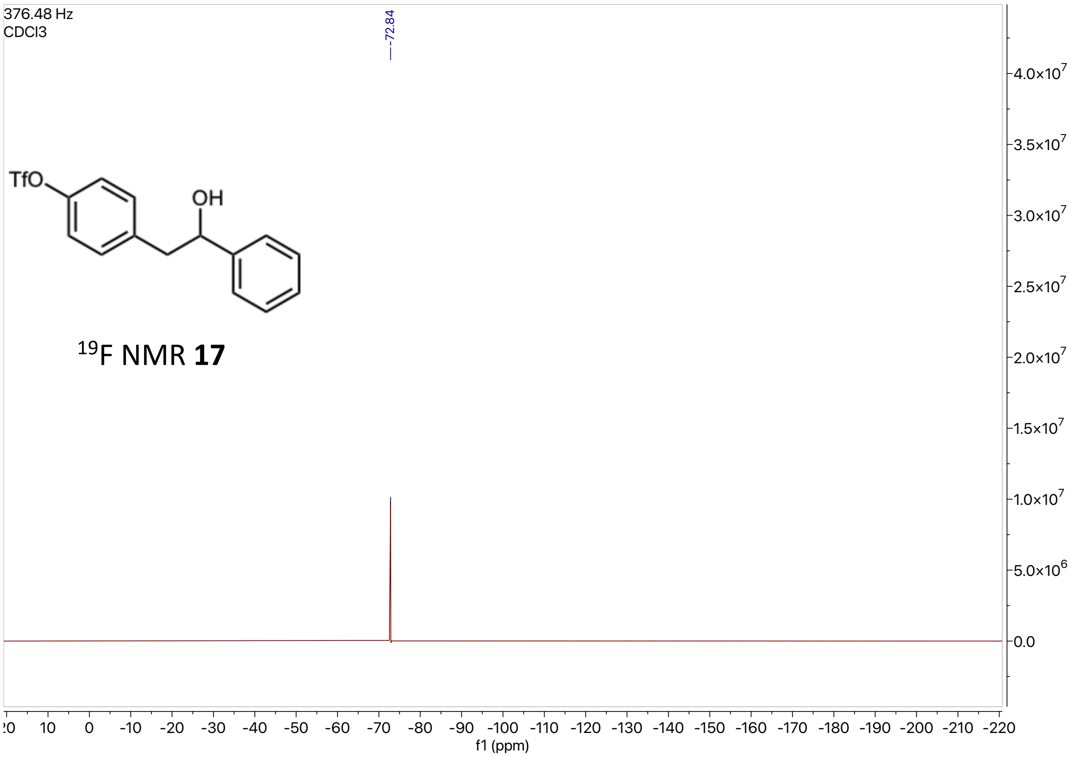

376.48 Hz
CDCl3
-72.84
TfO
OH
19F NMR 17
4.0×10^7
3.5×10^7
3.0×10^7
2.5×10^7
2.0×10^7
1.5×10^7
1.0×10^7
5.0×10^6
0.0
10 0 -10 -20 -30 -40 -50 -60 -70 -80 -90 -100 -110 -120 -130 -140 -150 -160 -170 -180 -190 -200 -210 -220
f1 (ppm)

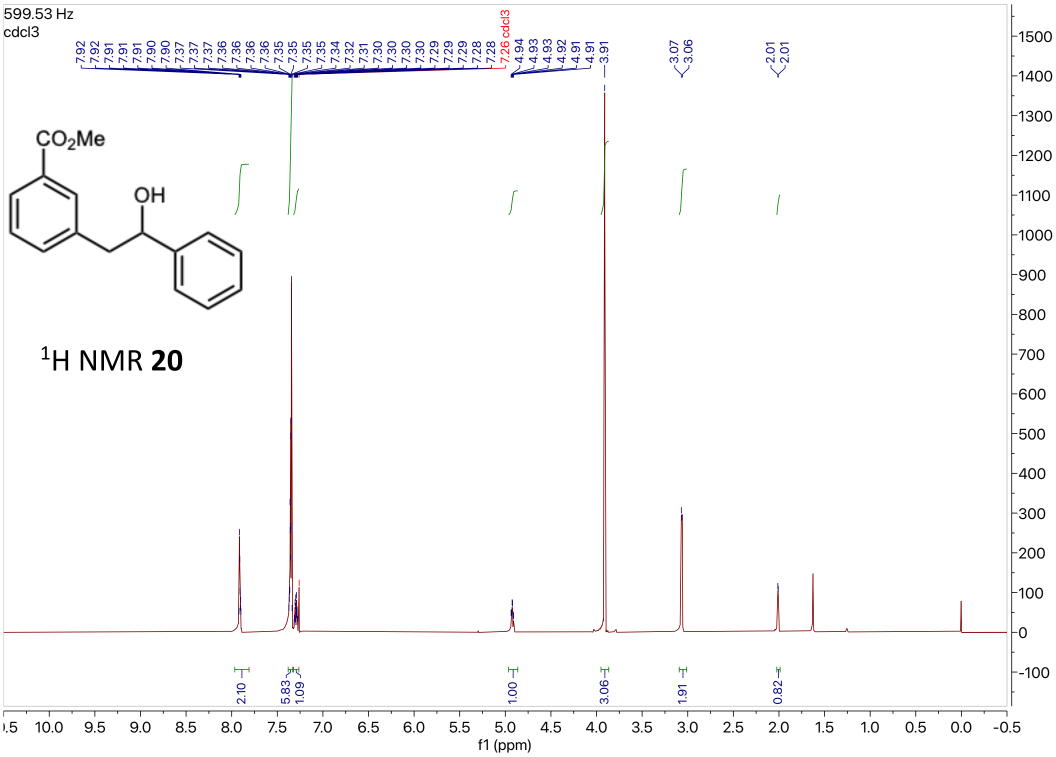
599.53 Hz
cdcl3
1H NMR 20
CO2Me
OH
f1 (ppm)

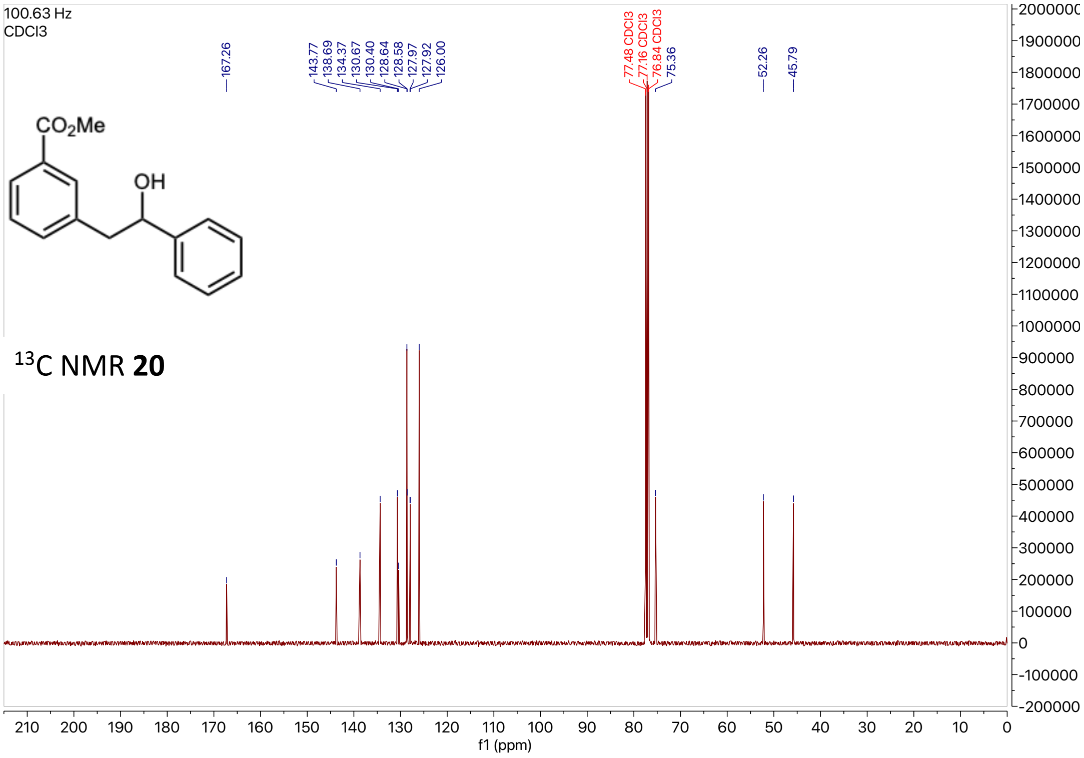
100.63 Hz
CDCl3
13C NMR 20
CO2Me
OH
f1 (ppm)

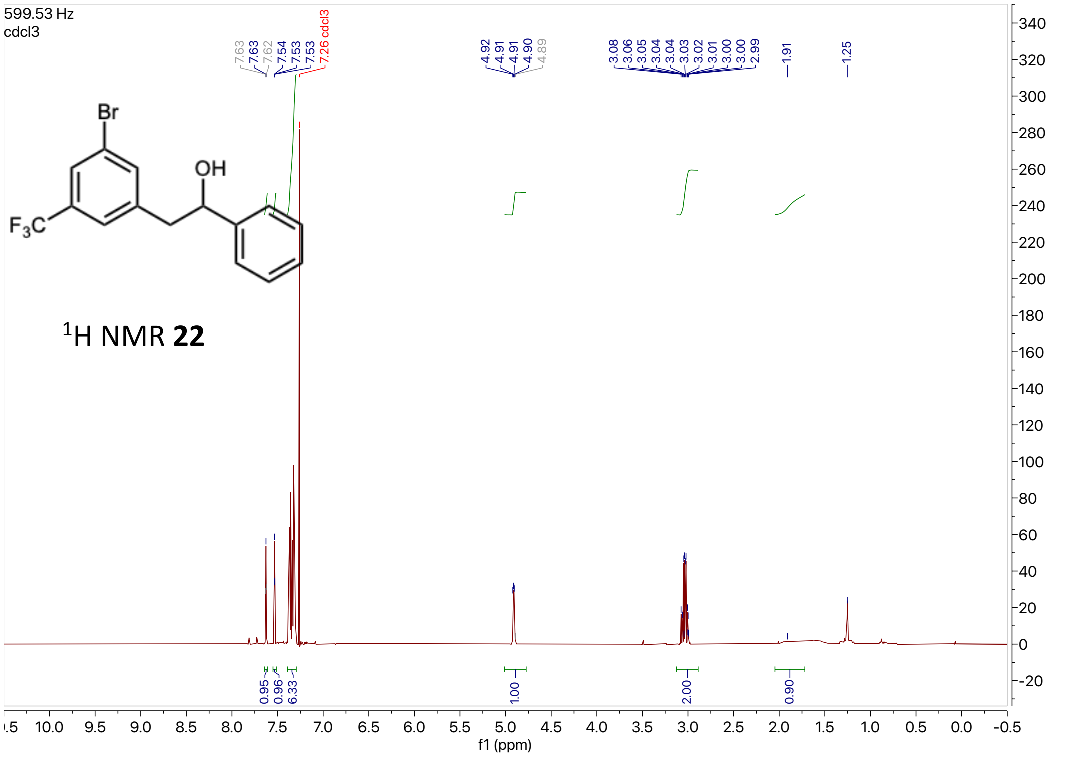
599.53 Hz
cdcl3
Br
OH
F3C
1H NMR 22
f1 (ppm)

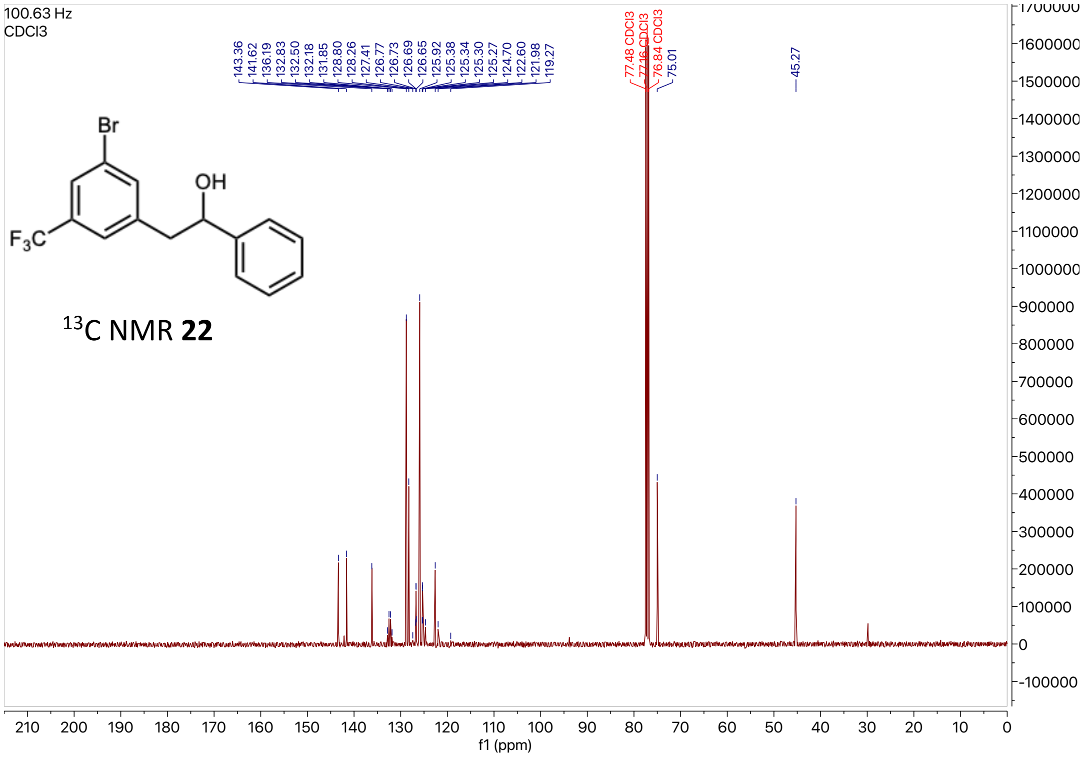
100.63 Hz
CDCl3
Br
OH
F3C
13C NMR 22
f1 (ppm)

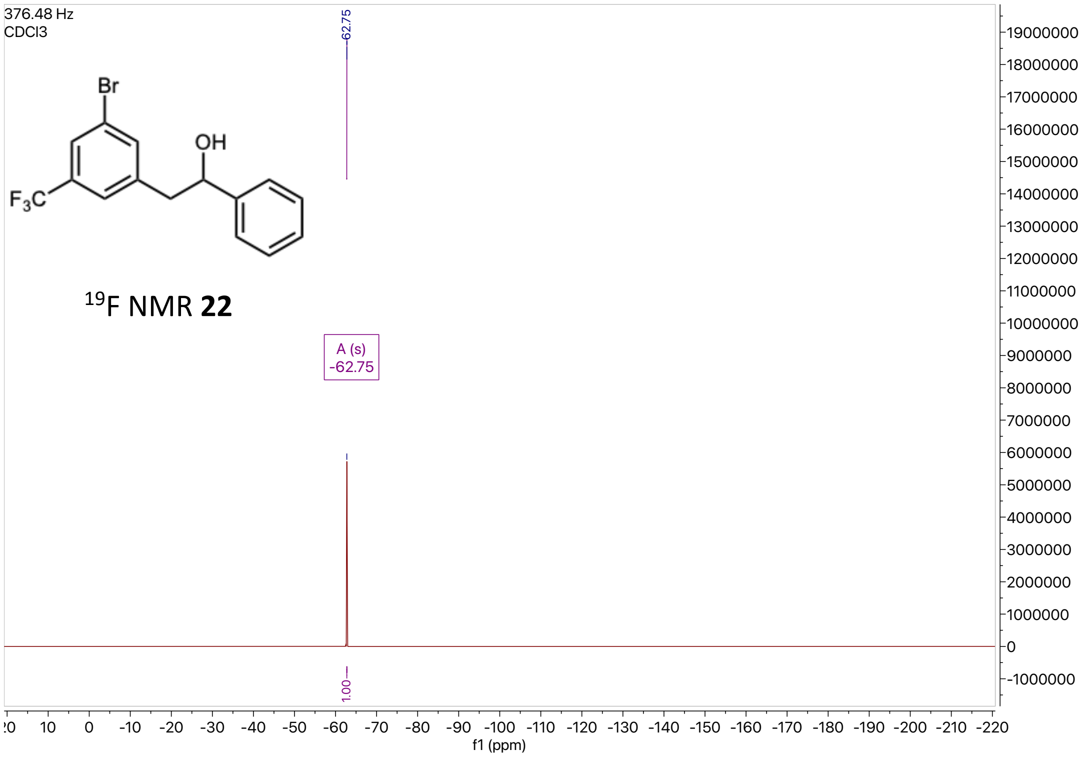
376.48 Hz
CDCl3
Br
OH
F3C
19F NMR 22
-62.75
A (s)
-62.75
1.00
f1 (ppm)

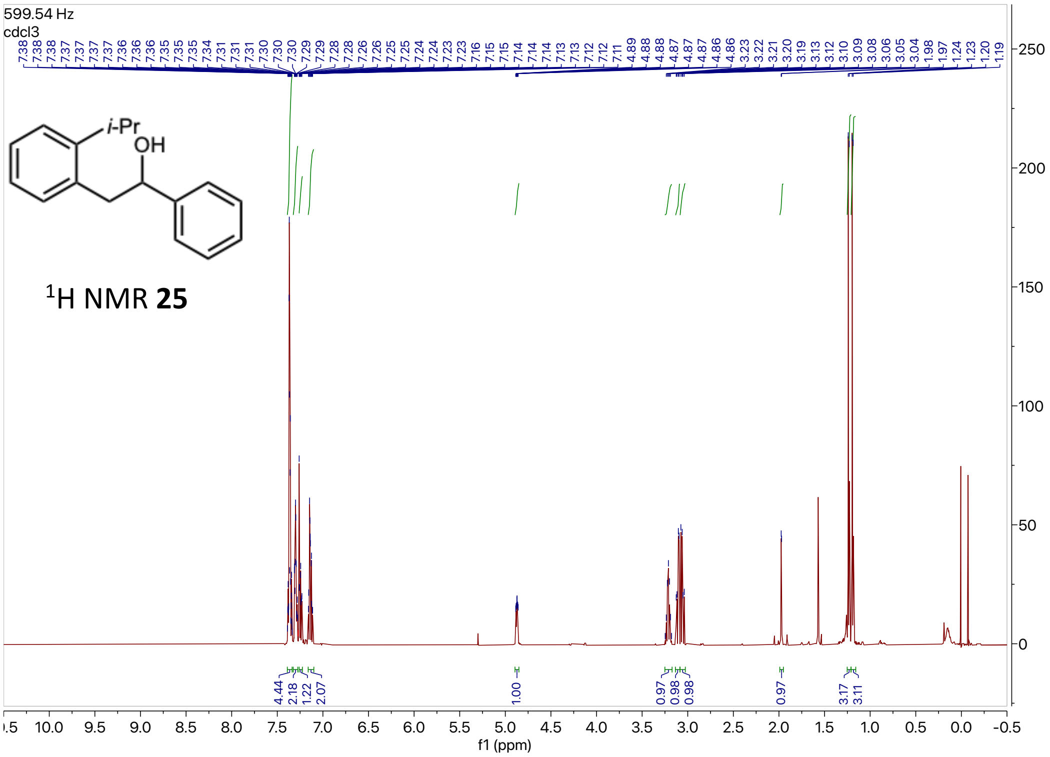
599.54 Hz
cdcl3
i-Pr
OH
^{1}H NMR **25**
f1 (ppm)

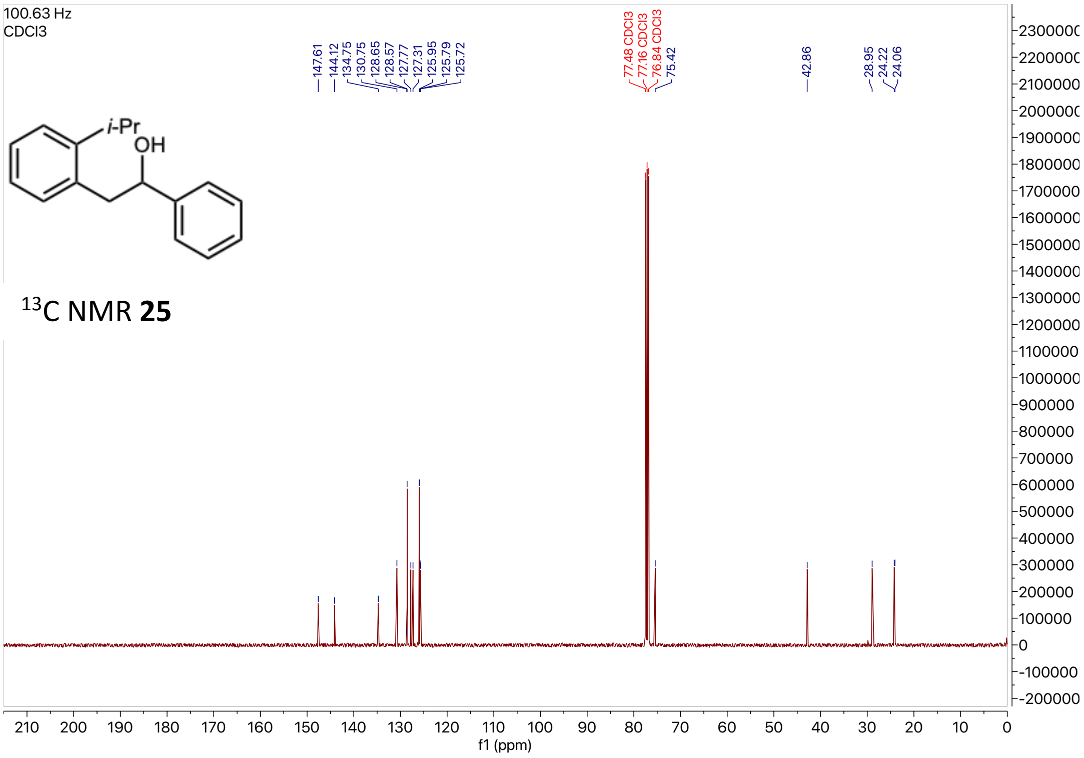
100.63 Hz
CDCl3
147.61
144.12
134.75
130.75
128.65
128.57
127.77
127.31
125.95
125.79
125.72
77.48 CDCl3
77.16 CDCl3
76.84 CDCl3
75.42
42.86
28.95
24.22
24.06
i-Pr
OH
^{13}C NMR **25**
f1 (ppm)

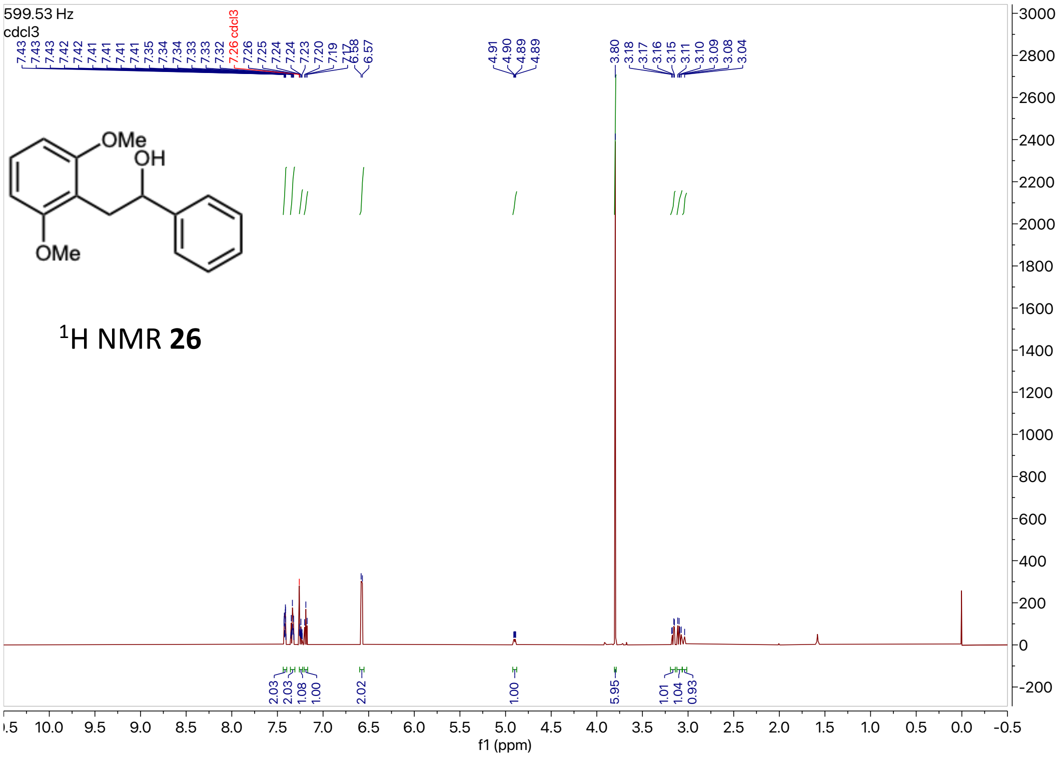
599.53 Hz
cdcl3
7.43 7.43 7.43 7.42 7.42 7.41 7.41 7.41 7.41 7.35 7.34 7.34 7.33 7.33 7.33 7.32 7.26 cdcl3 7.26 7.25 7.24 7.24 7.23 7.20 7.19 7.17 6.58 6.57
4.91 4.90 4.89 4.89
3.80 3.18 3.17 3.16 3.15 3.11 3.10 3.09 3.08 3.04
OMe
OH
OMe
^{1}H NMR **26**
2.03 2.03 1.08 1.00 2.02 1.00 5.95 1.01 1.04 0.93
f1 (ppm)

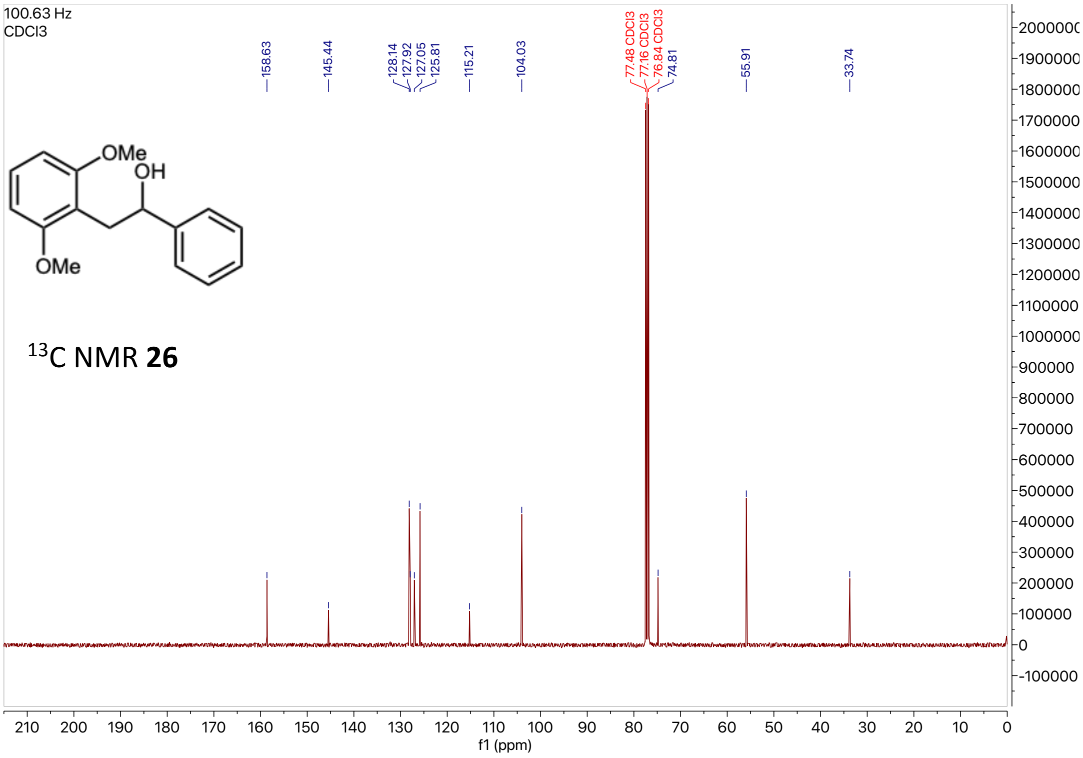
100.63 Hz
CDCl3
158.63
145.44
128.14 127.92 127.05 125.81
115.21
104.03
77.48 CDCl3 77.16 CDCl3 76.84 CDCl3 74.81
55.91
33.74
OMe
OH
OMe
^{13}C NMR **26**
f1 (ppm)

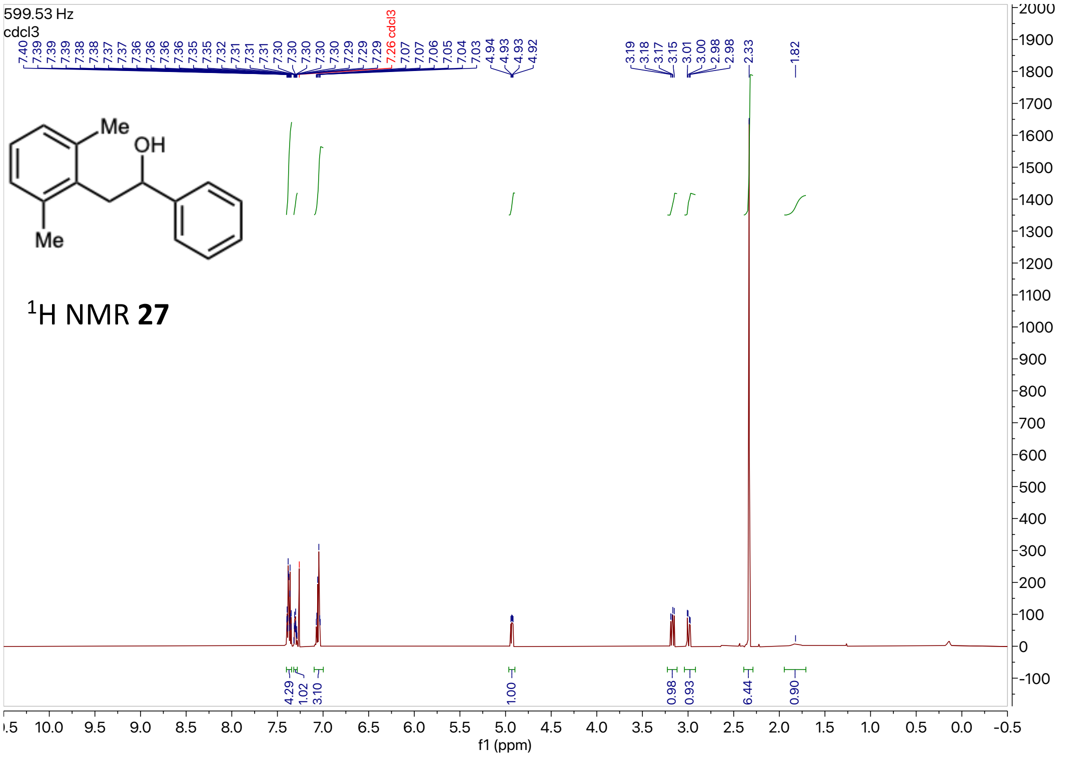

599.53 Hz
cdcl3
^{1}H NMR **27**
f1 (ppm)

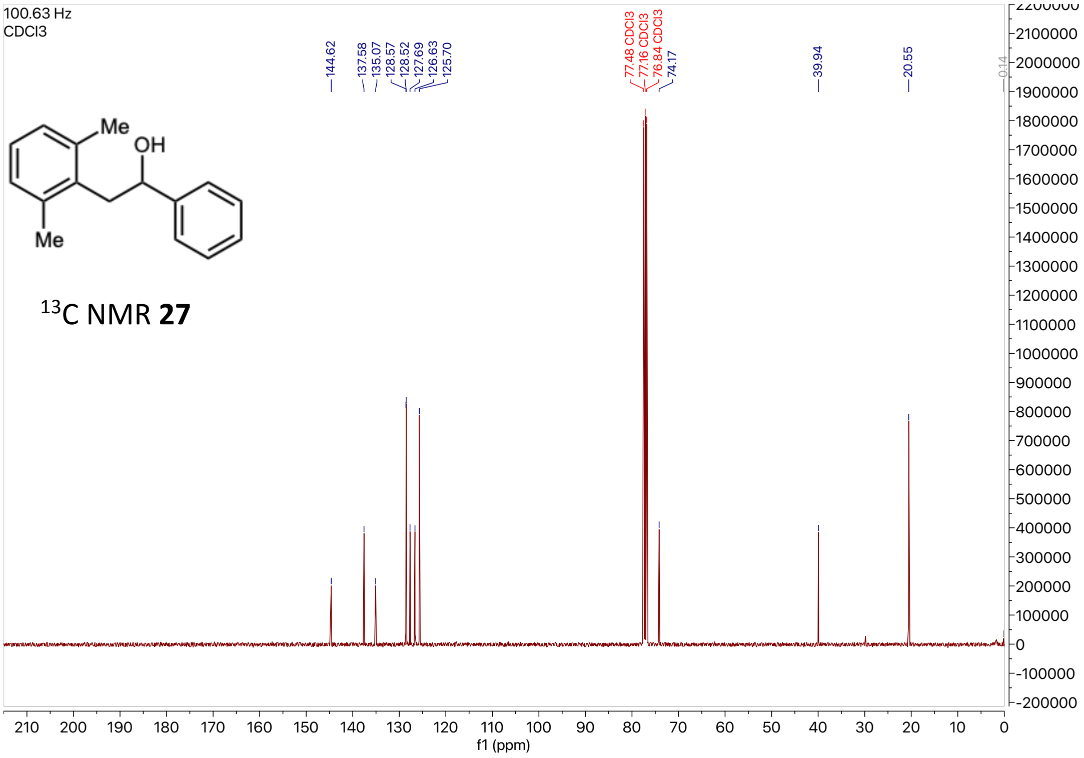

100.63 Hz
CDCl3
^{13}C NMR **27**
f1 (ppm)

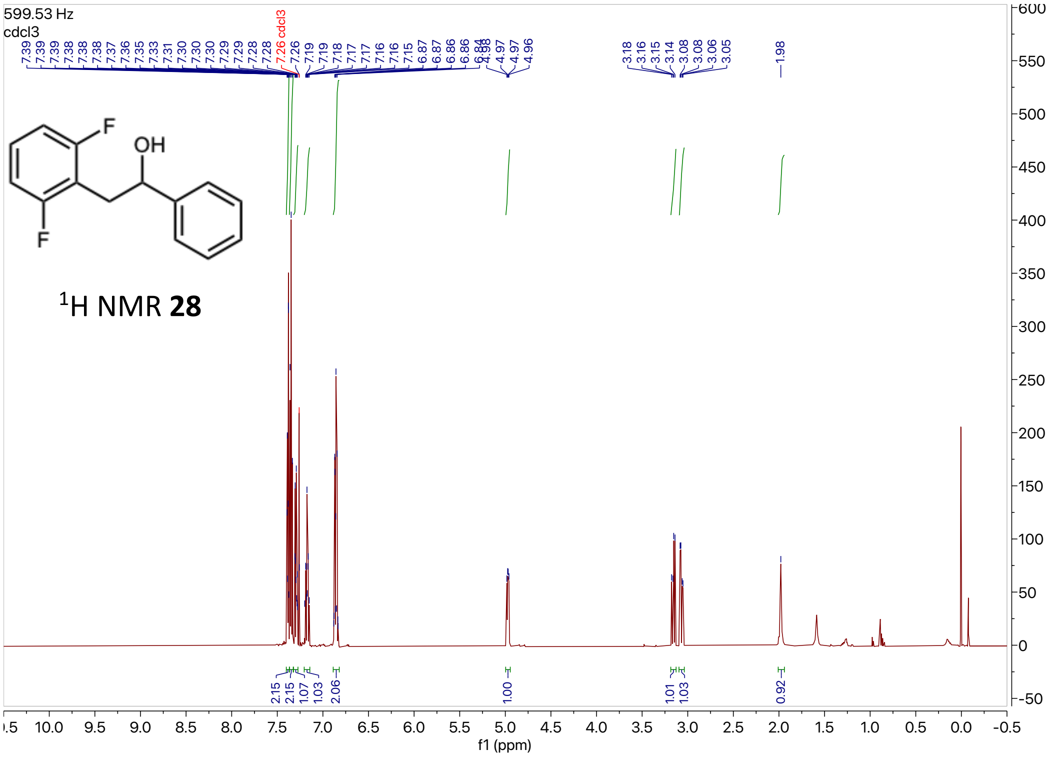
1H NMR 28
599.53 Hz
cdcl3
f1 (ppm)

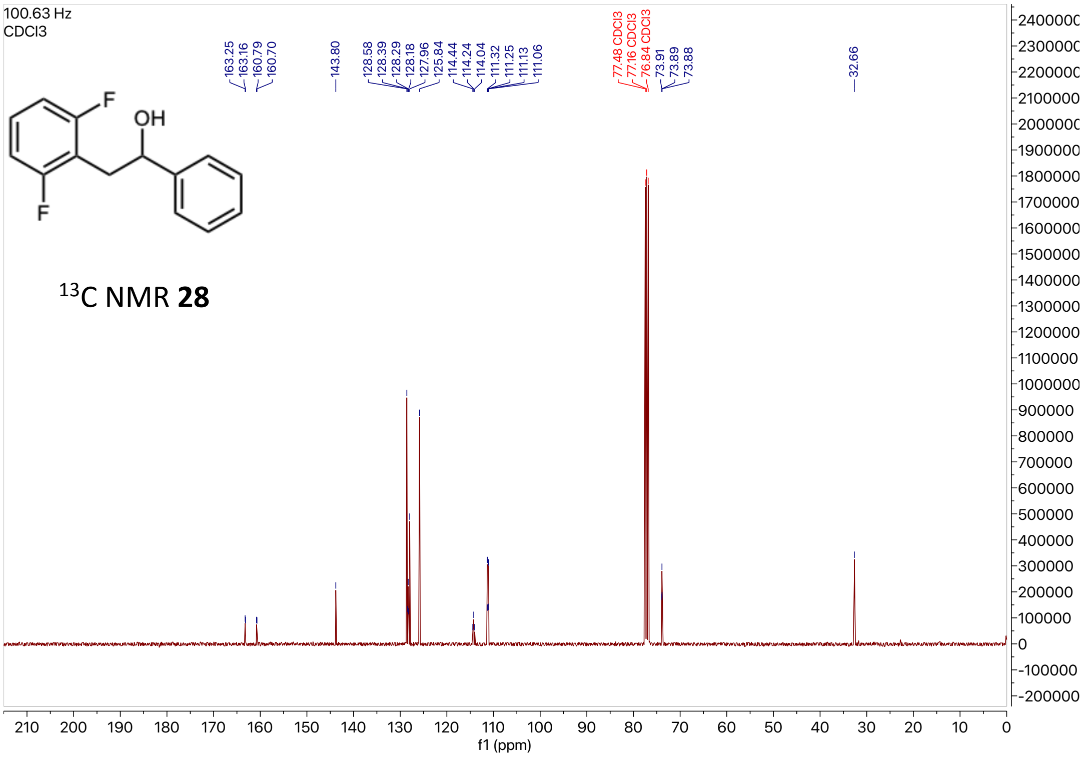
13C NMR 28
100.63 Hz
CDCl3
f1 (ppm)

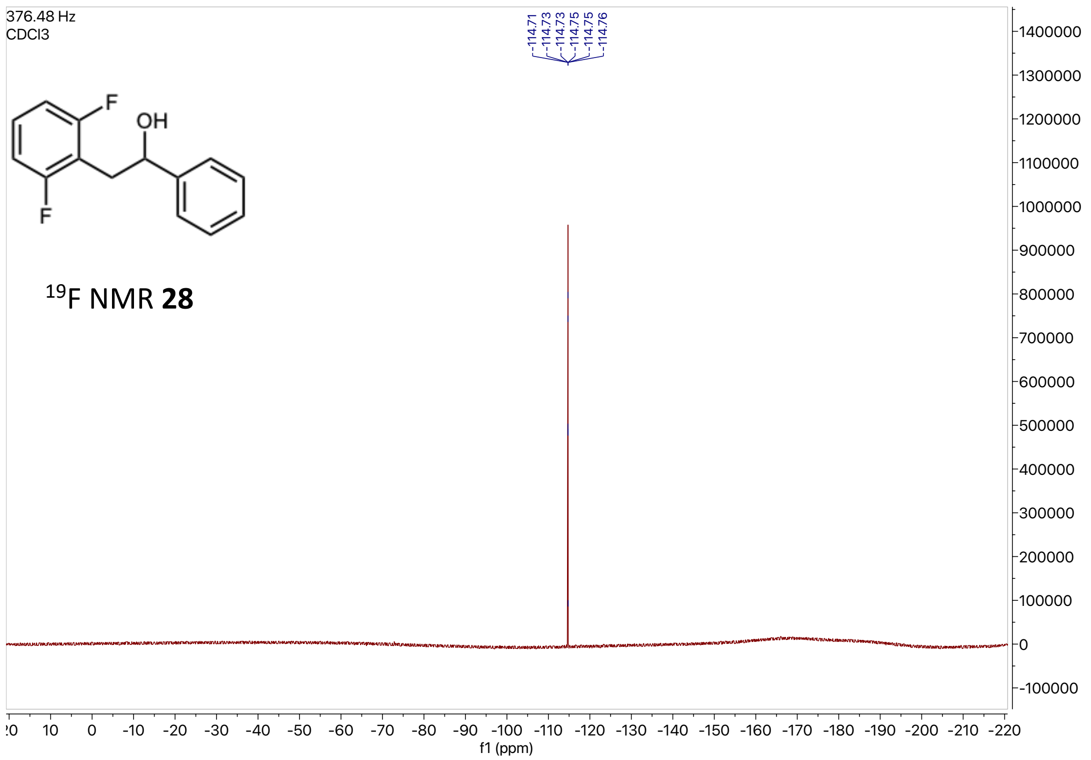
376.48 Hz
CDCl3
-114.71
-114.73
-114.73
-114.75
-114.75
-114.76
F
OH
F
19F NMR 28
1400000
1300000
1200000
1100000
1000000
900000
800000
700000
600000
500000
400000
300000
200000
100000
0
-100000
10 0 -10 -20 -30 -40 -50 -60 -70 -80 -90 -100 -110 -120 -130 -140 -150 -160 -170 -180 -190 -200 -210 -220
f1 (ppm)

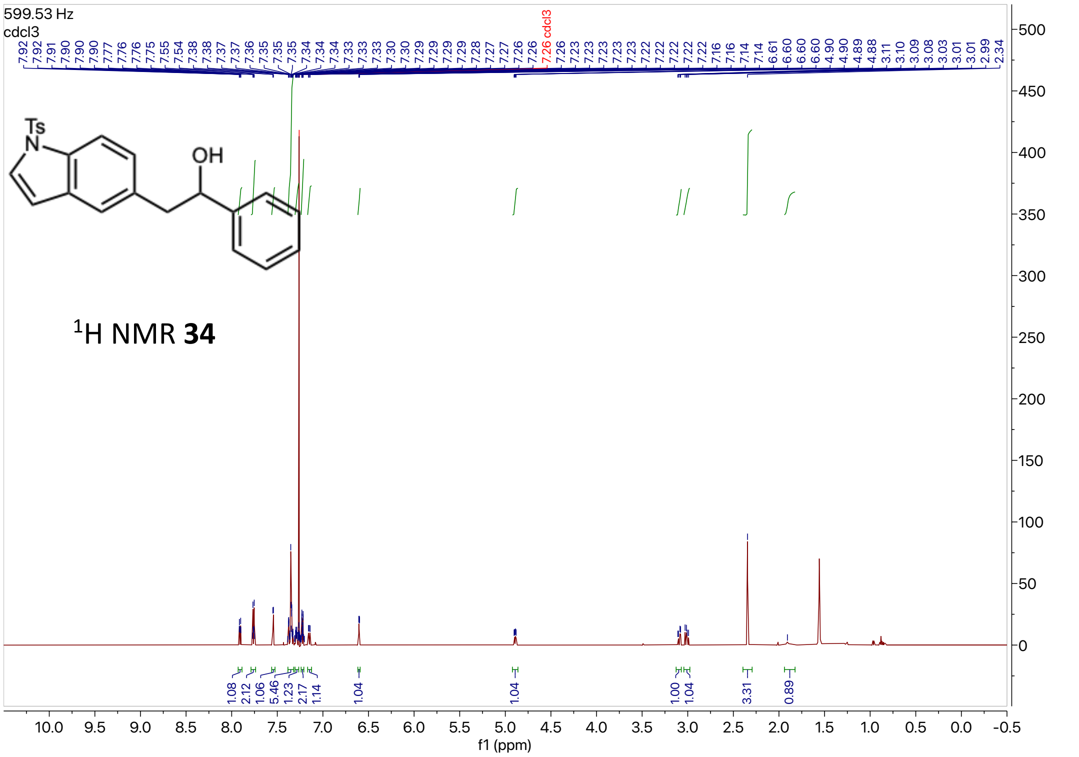

^{1}H NMR **34**

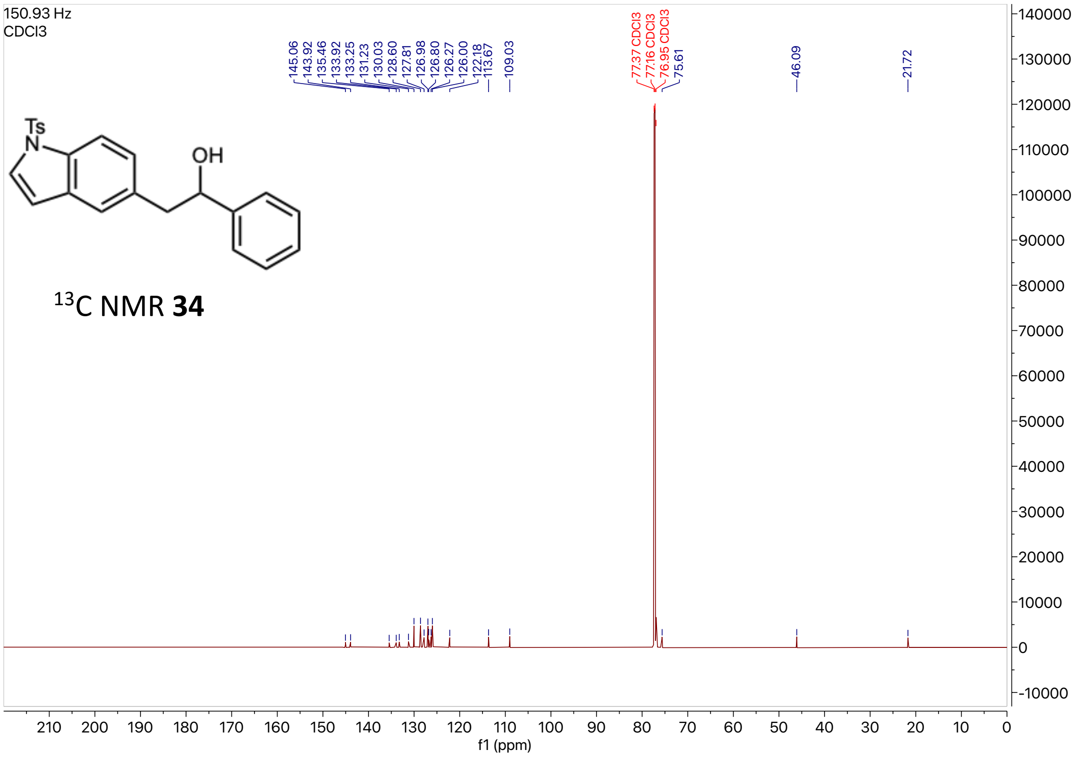

^{13}C NMR **34**

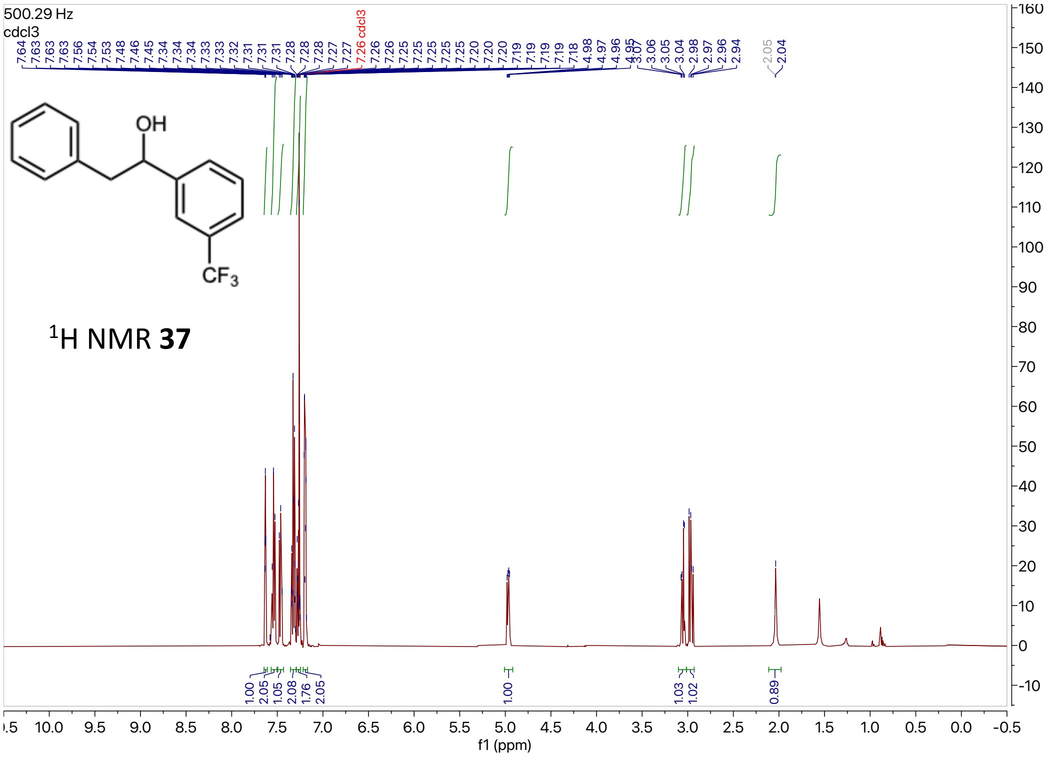

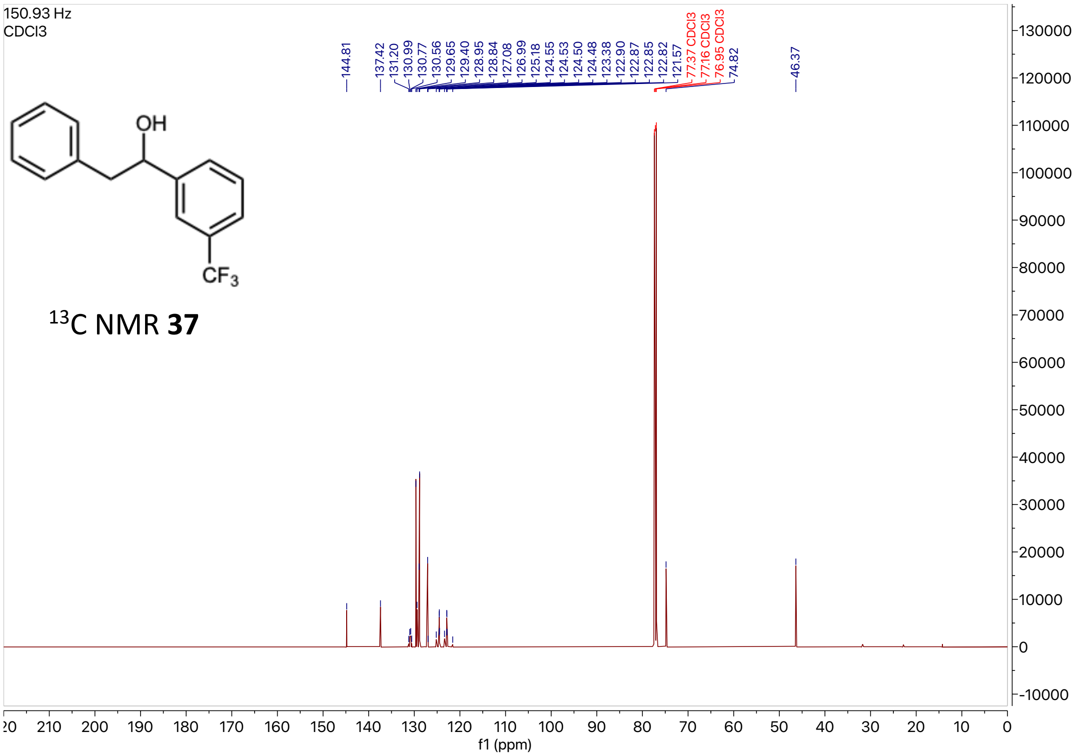

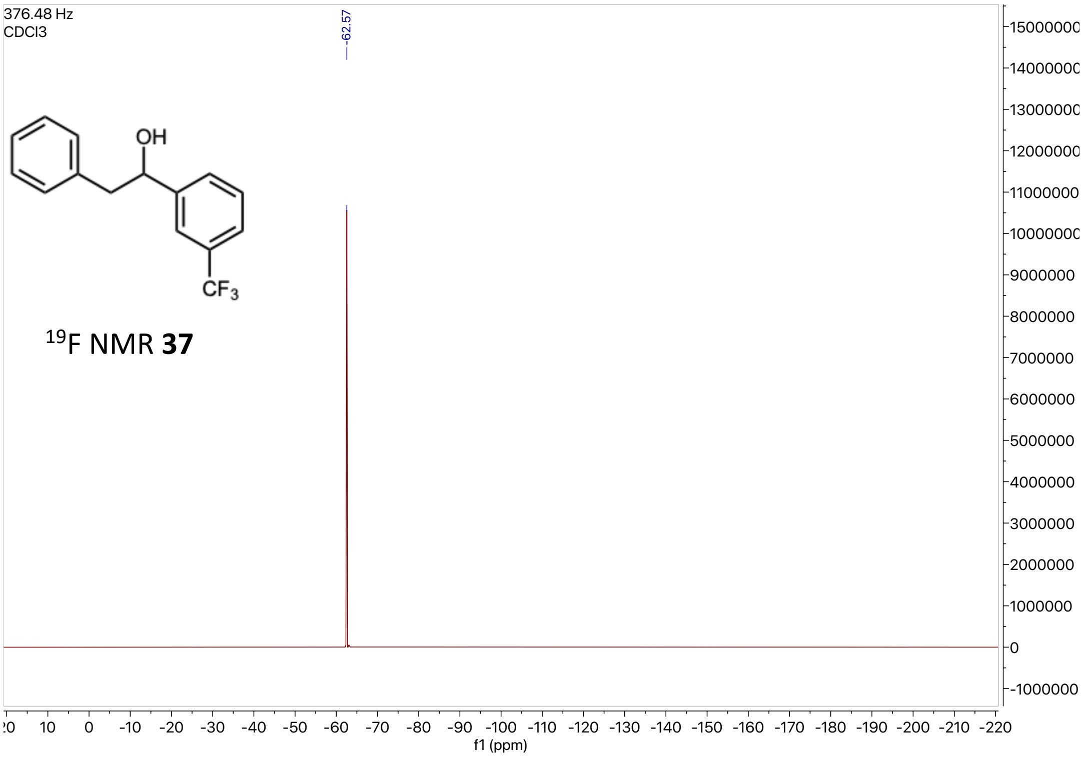

^{19}F NMR **37**

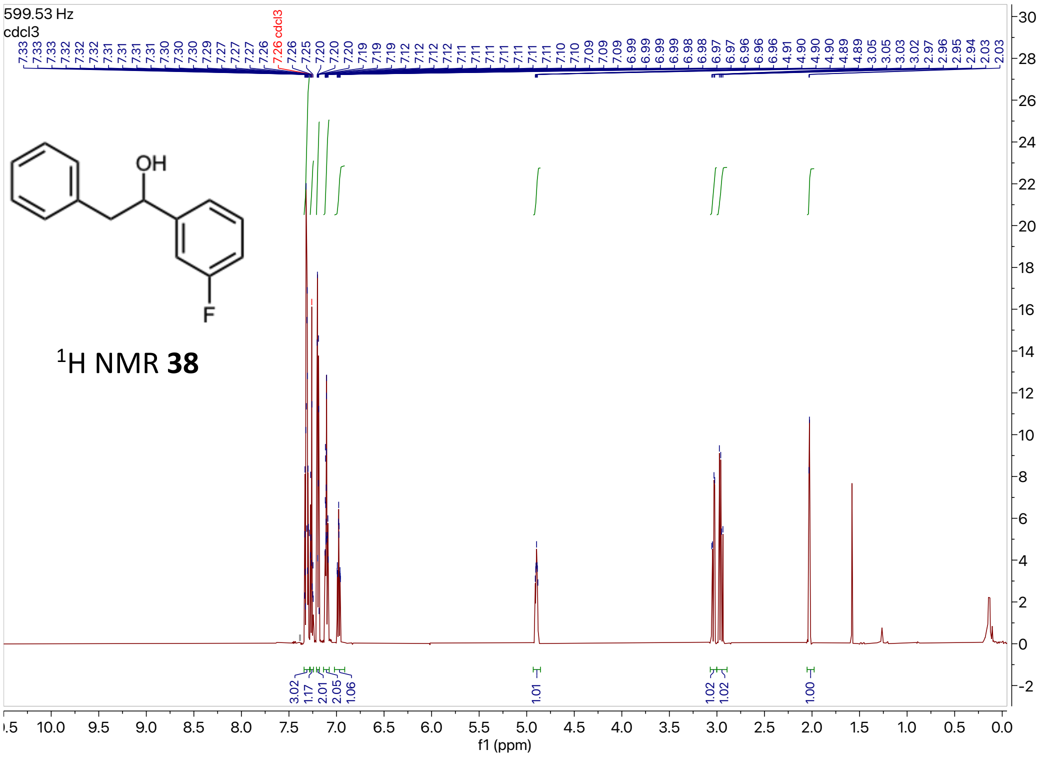

599.53 Hz
cdcl3
OH
F
1H NMR 38
f1 (ppm)

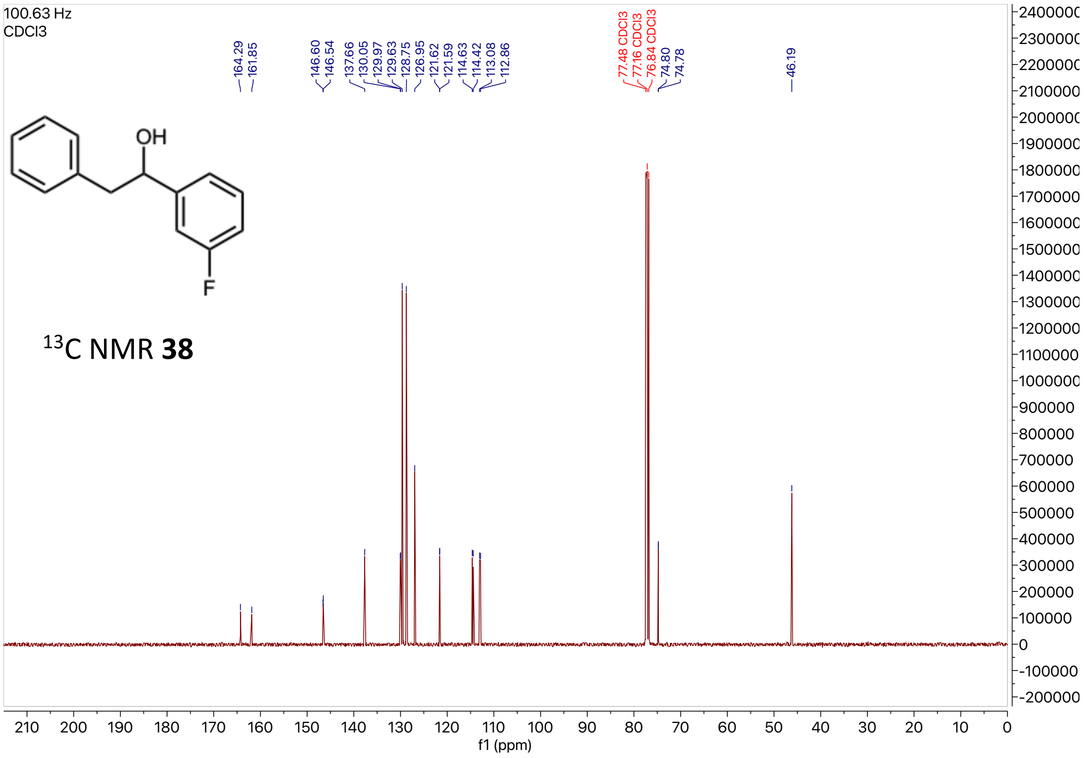

100.63 Hz
CDCl3
164.29
161.85
146.60
146.54
137.66
130.05
129.97
129.63
128.75
126.95
121.62
121.59
114.63
114.42
113.08
112.86
77.48 CDCl3
77.16 CDCl3
76.84 CDCl3
74.80
74.78
46.19
OH
F
13C NMR 38
f1 (ppm)

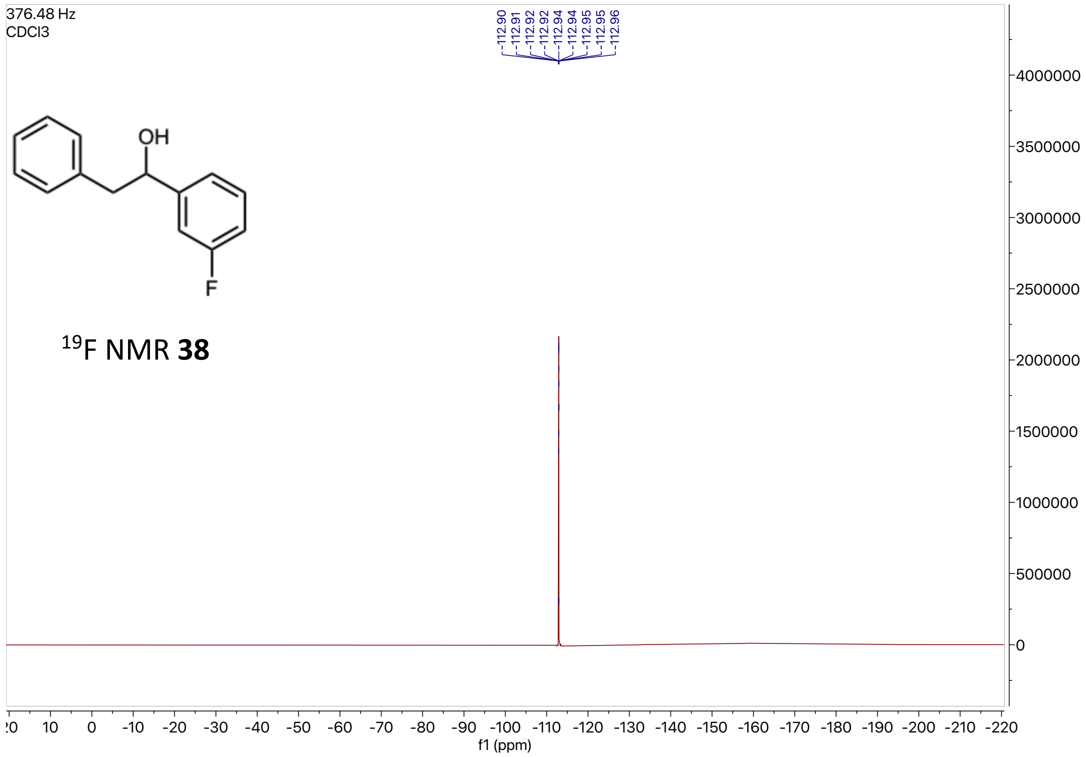
376.48 Hz
CDCl3
-112.90
-112.91
-112.92
-112.92
-112.94
-112.94
-112.95
-112.95
-112.96
OH
F
^{19}F NMR **38**
4000000
3500000
3000000
2500000
2000000
1500000
1000000
500000
0
10 0 -10 -20 -30 -40 -50 -60 -70 -80 -90 -100 -110 -120 -130 -140 -150 -160 -170 -180 -190 -200 -210 -220
f1 (ppm)

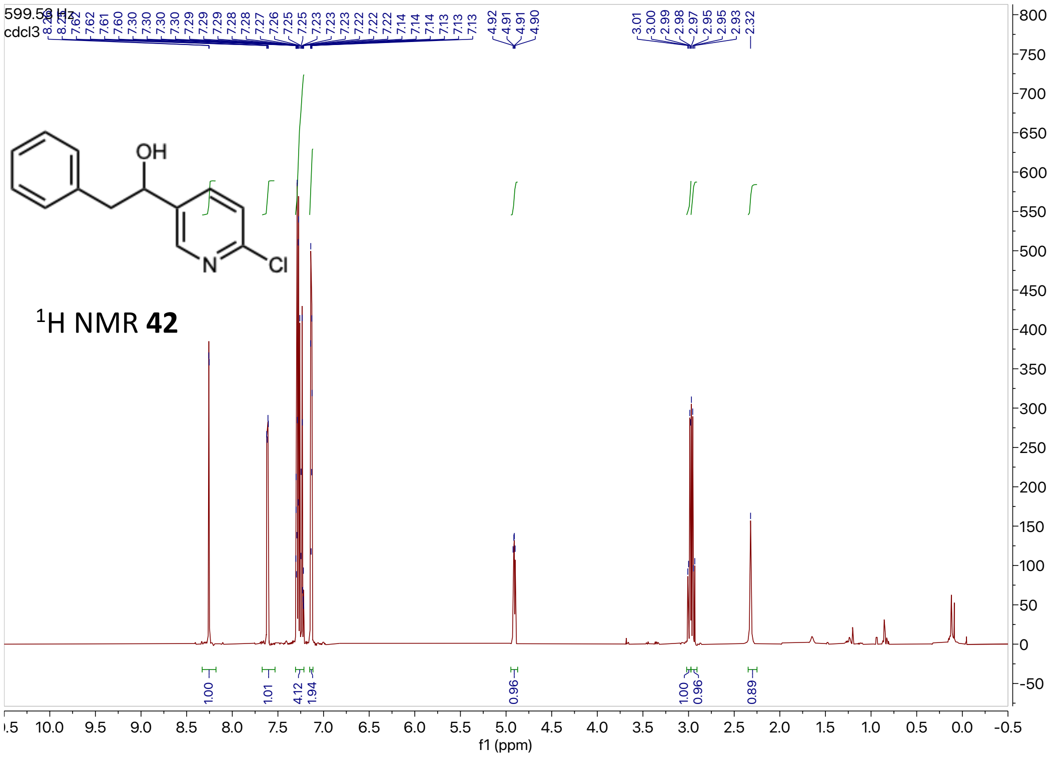

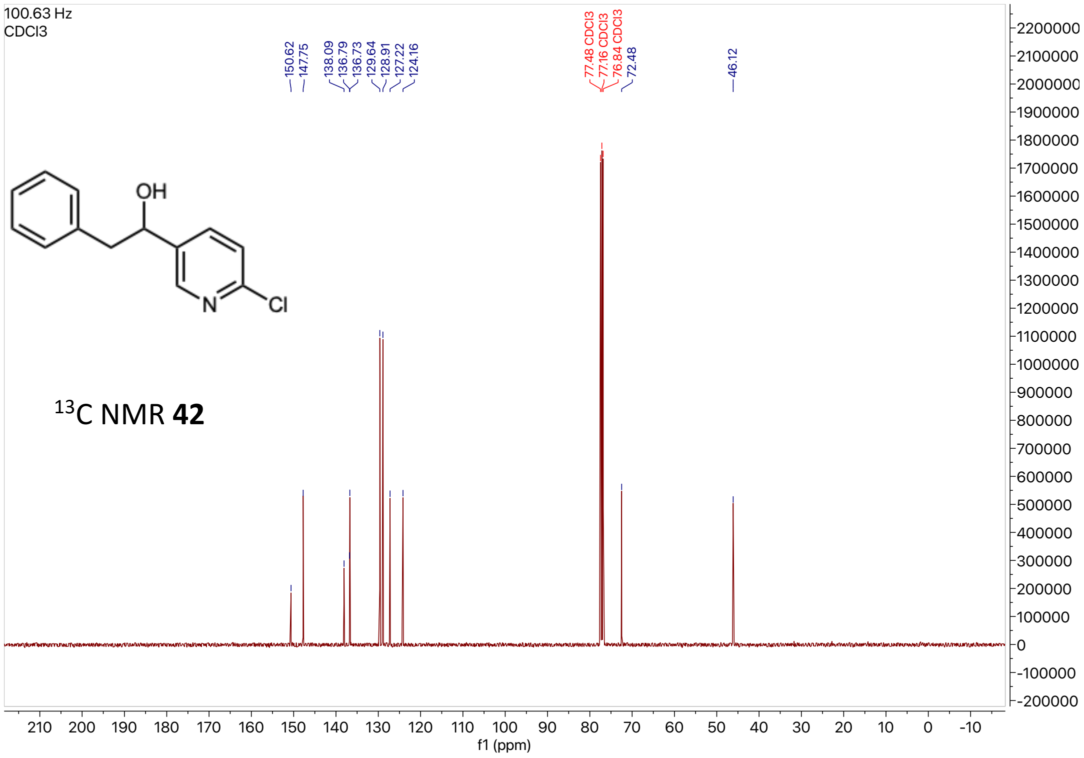

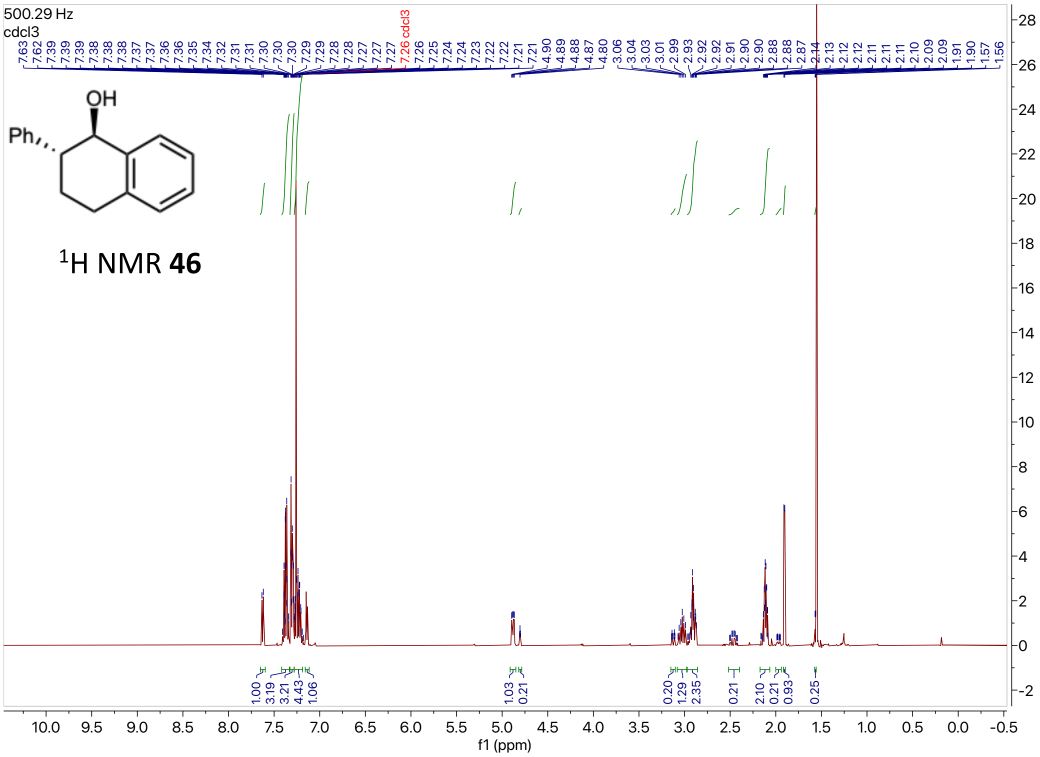

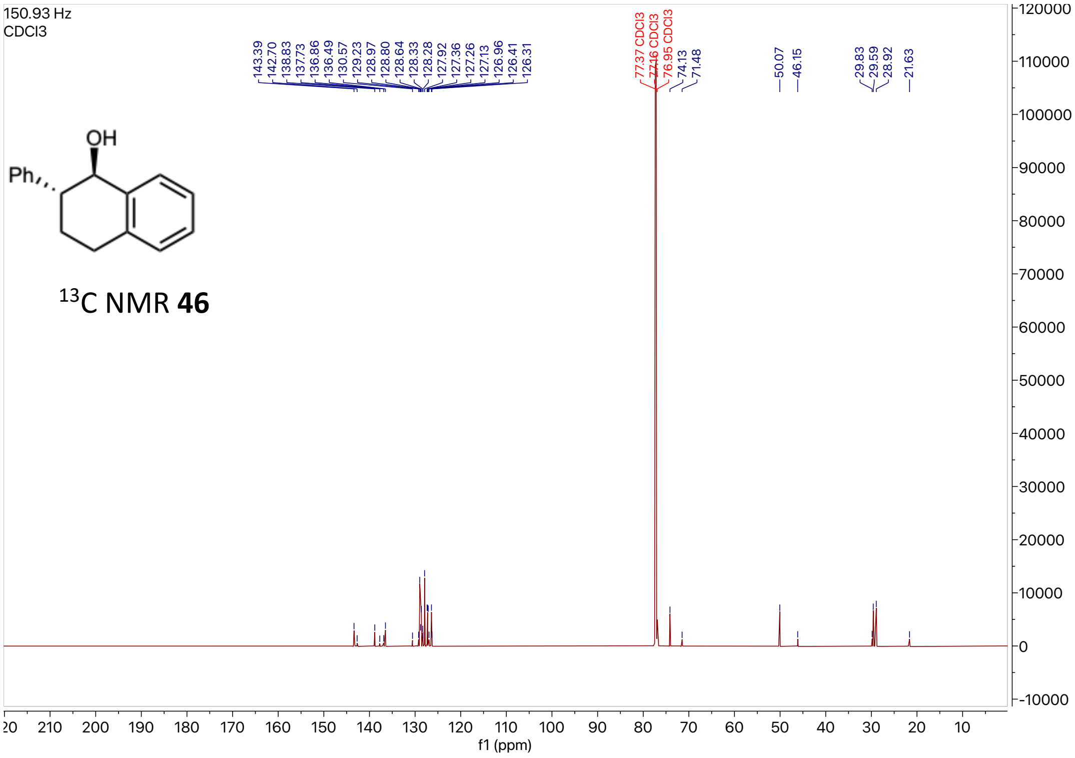

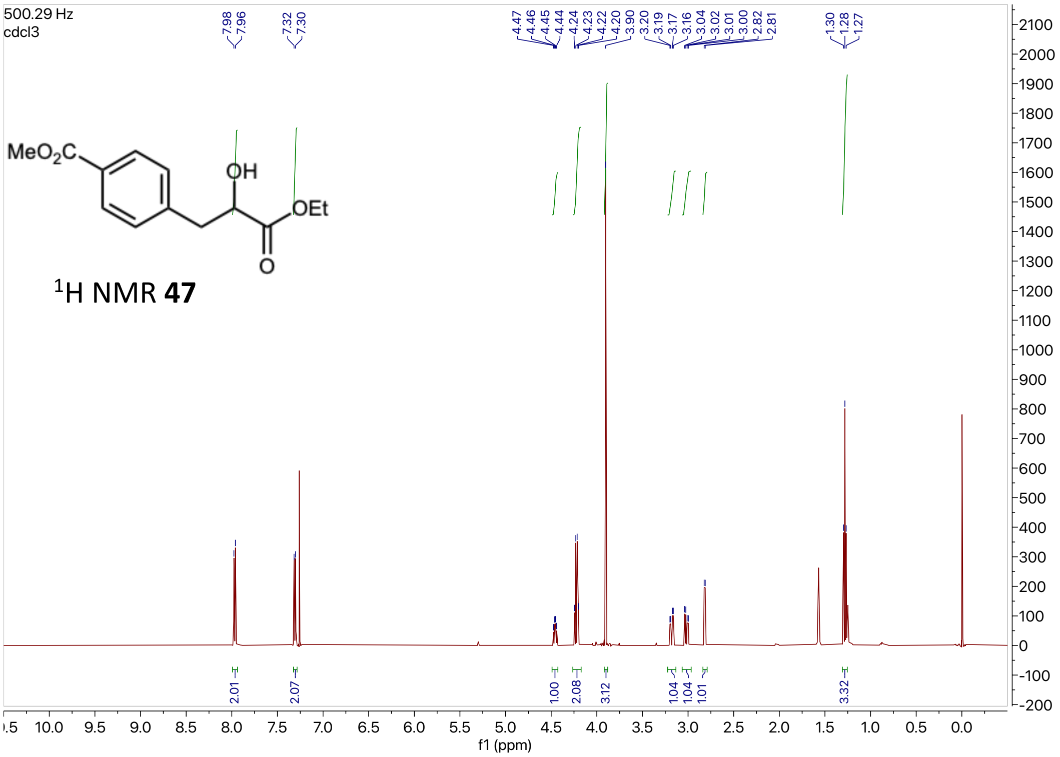

^{1}H NMR **47**

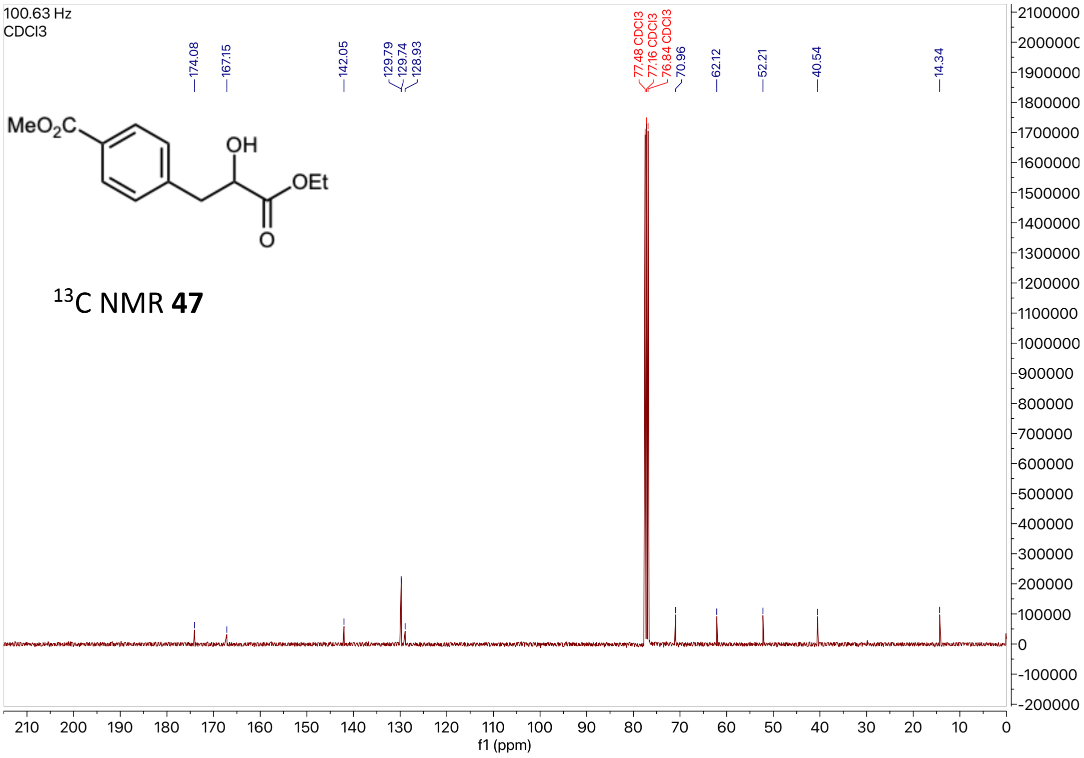

^{13}C NMR **47**

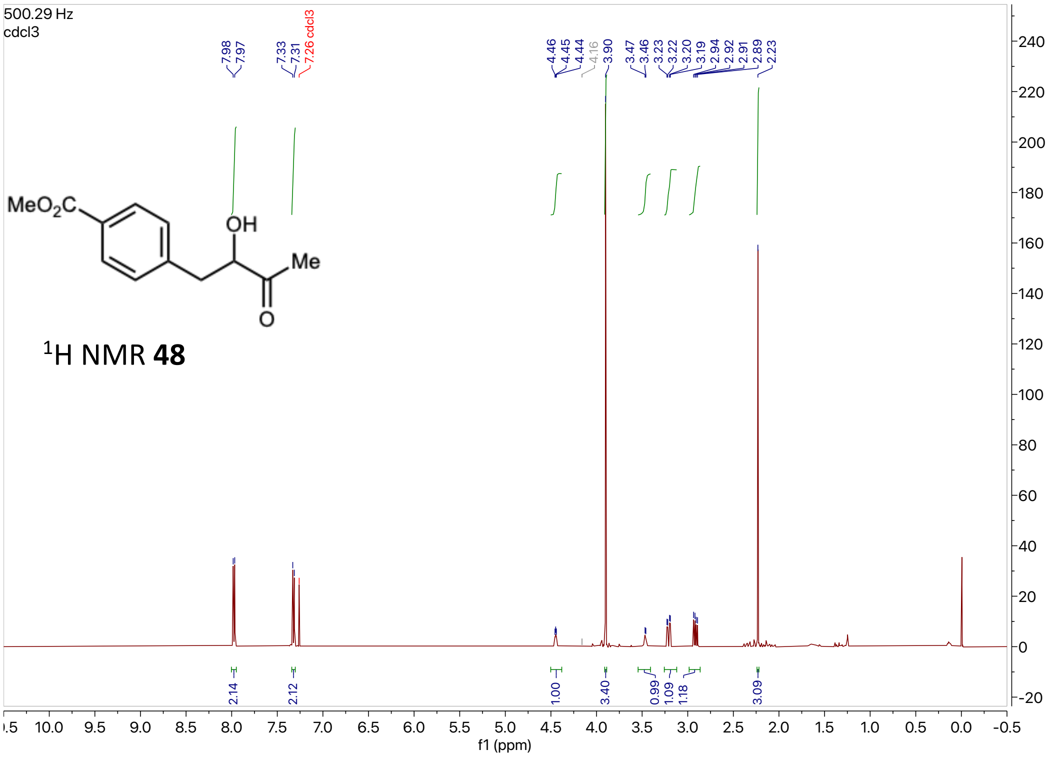

^{1}H NMR **48**

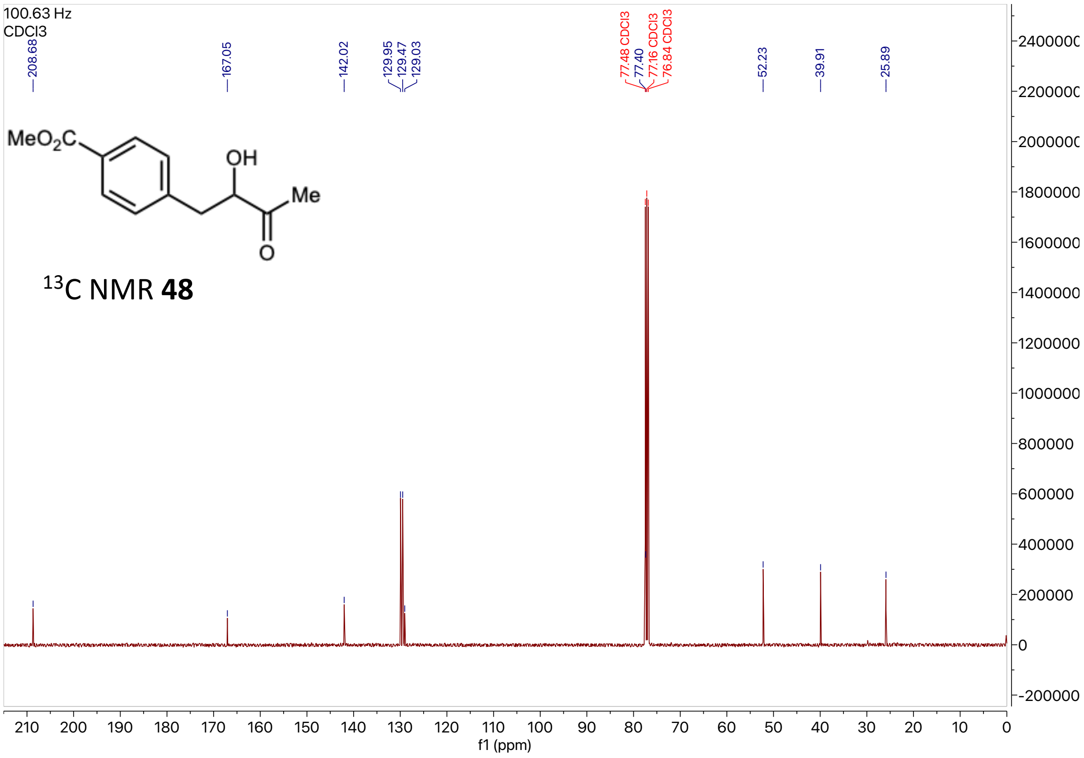

^{13}C NMR **48**

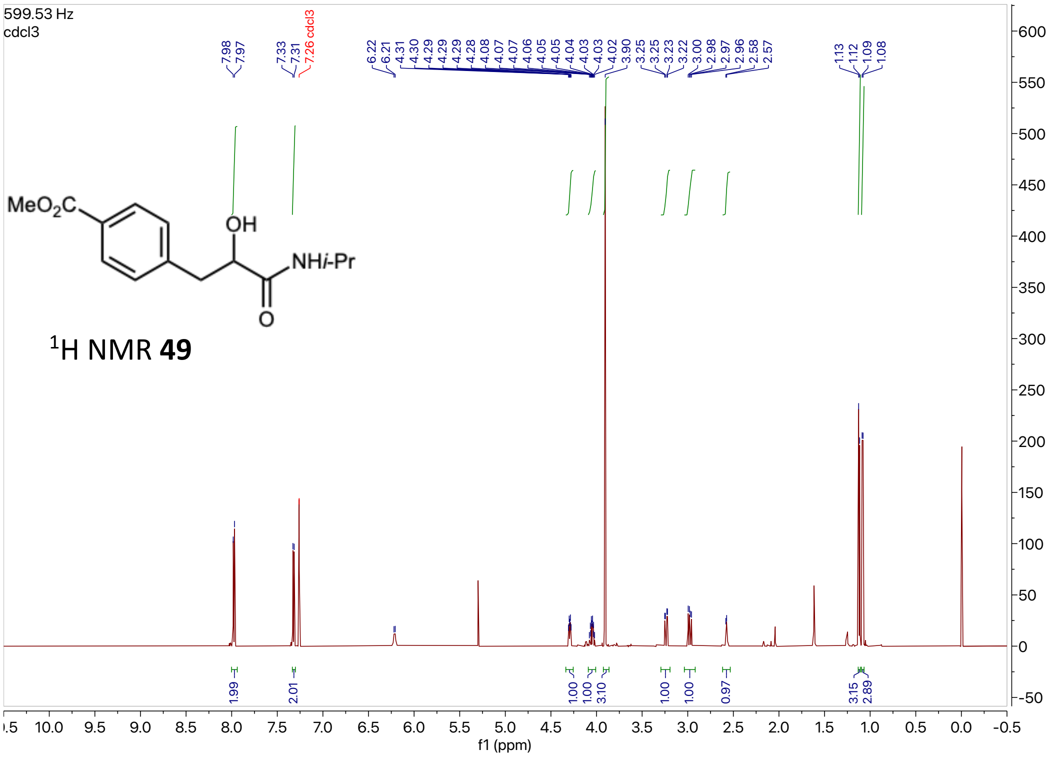

^{1}H NMR **49**

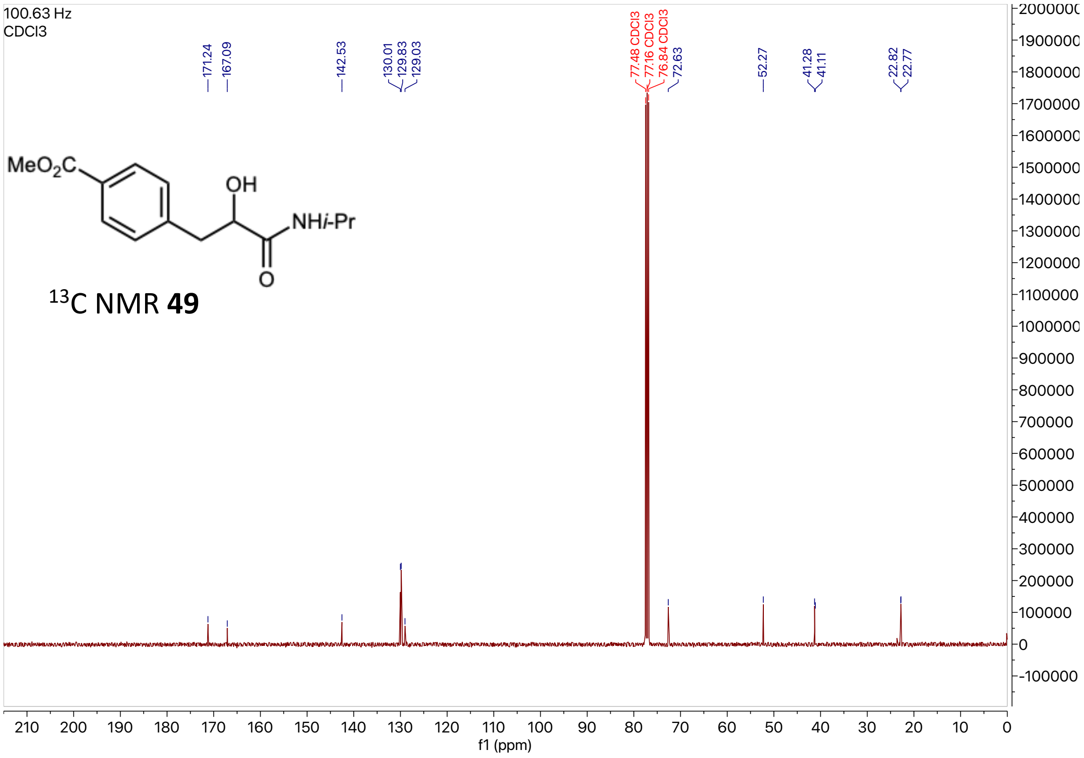

^{13}C NMR **49**

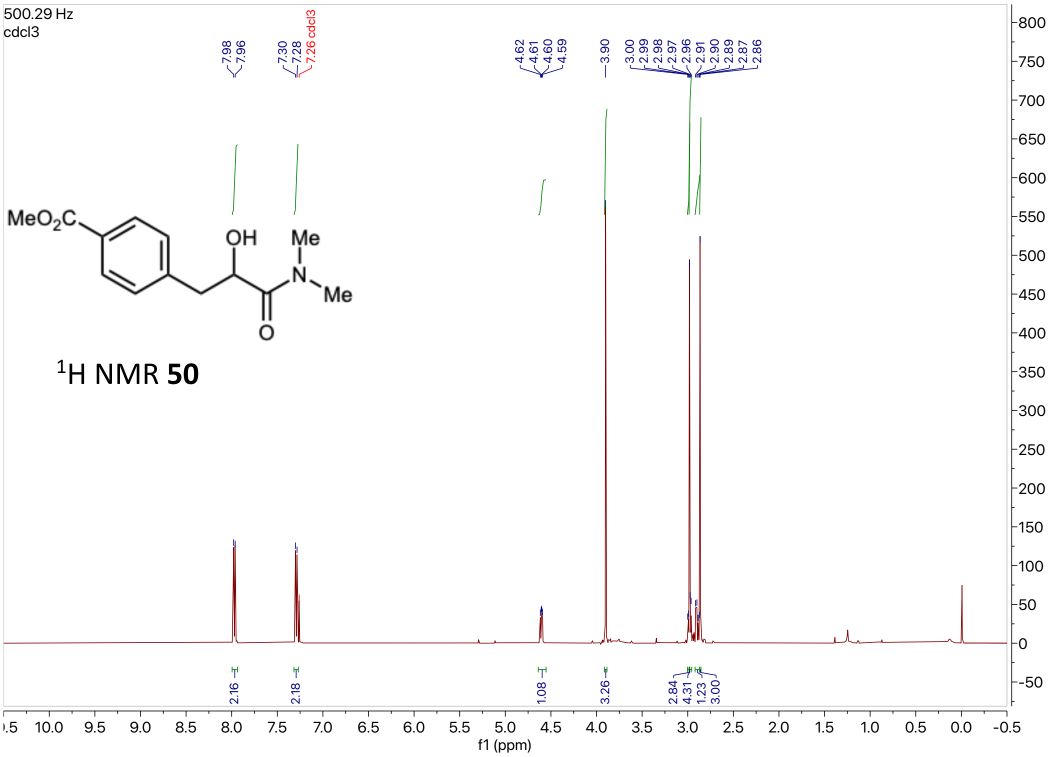
500.29 Hz
cdcl3
7.98
7.96
7.30
7.28
7.26 cdcl3
4.62
4.61
4.60
4.59
3.90
3.00
2.99
2.98
2.97
2.96
2.91
2.90
2.89
2.87
2.86
MeO2C
OH
Me
N
Me
O
1H NMR 50
2.16
2.18
1.08
3.26
2.84
4.31
1.23
3.00
f1 (ppm)

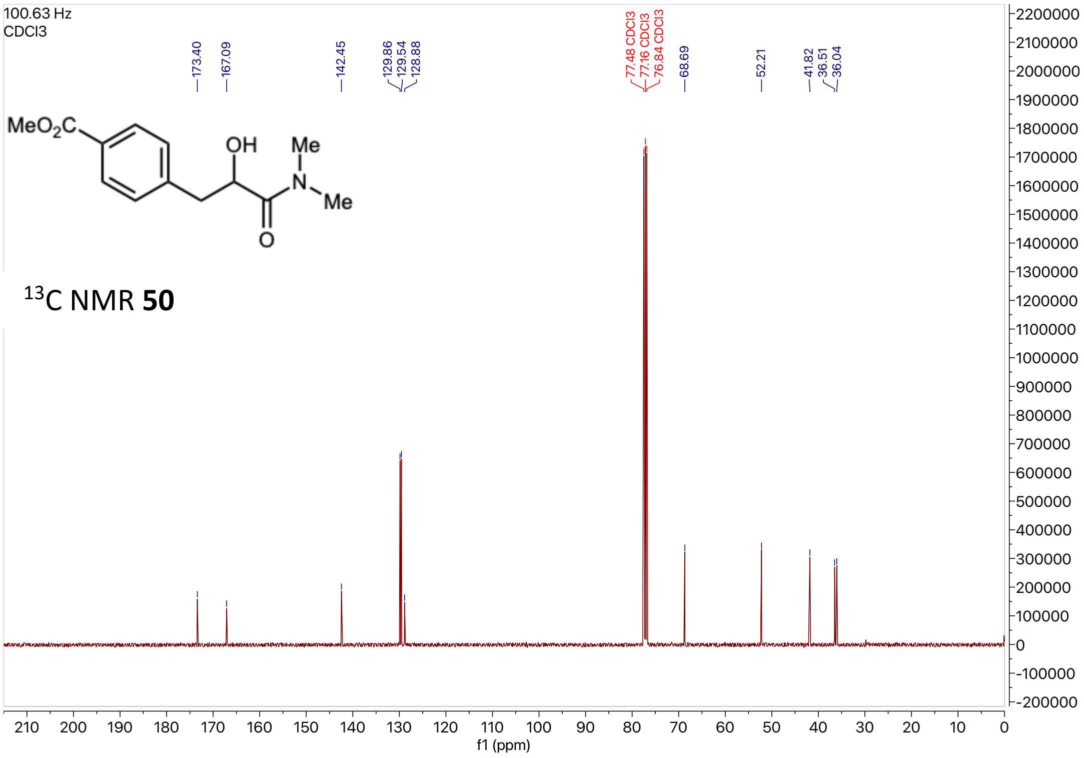
100.63 Hz
CDCl3
173.40
167.09
142.45
129.86
129.54
128.88
77.48 CDCl3
77.16 CDCl3
76.84 CDCl3
68.69
52.21
41.82
36.51
36.04
MeO2C
OH
Me
N
Me
O
13C NMR 50
f1 (ppm)

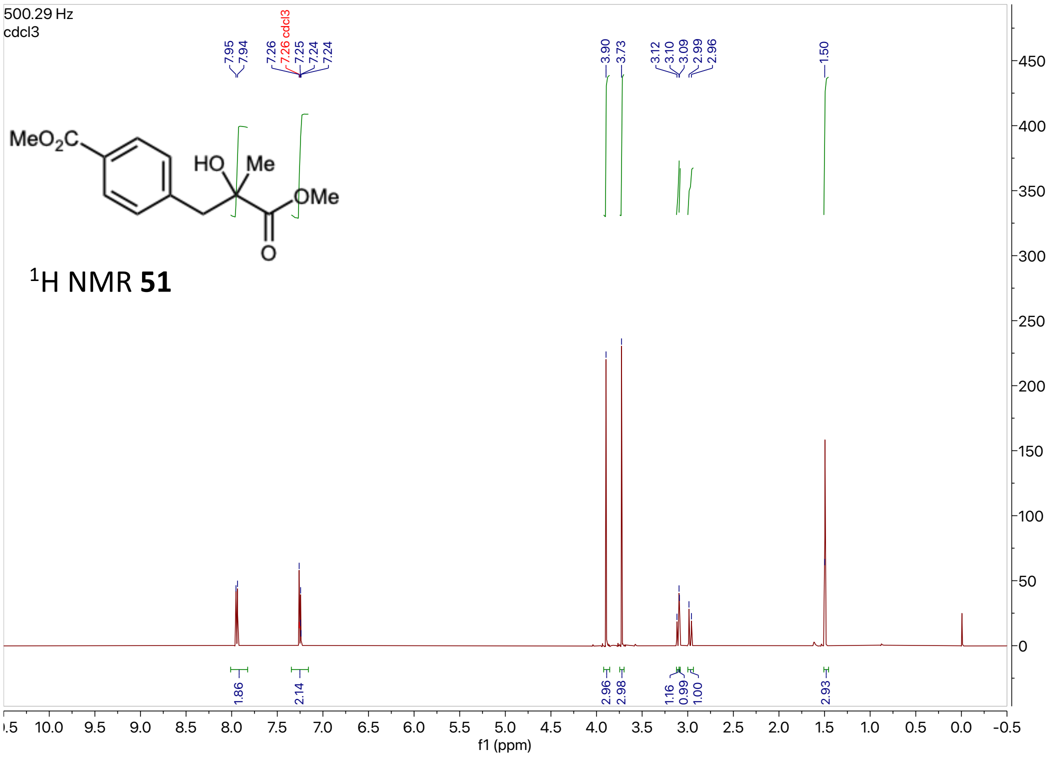
500.29 Hz
cdcl3
MeO2C
HO
Me
OMe
O
1H NMR 51
f1 (ppm)

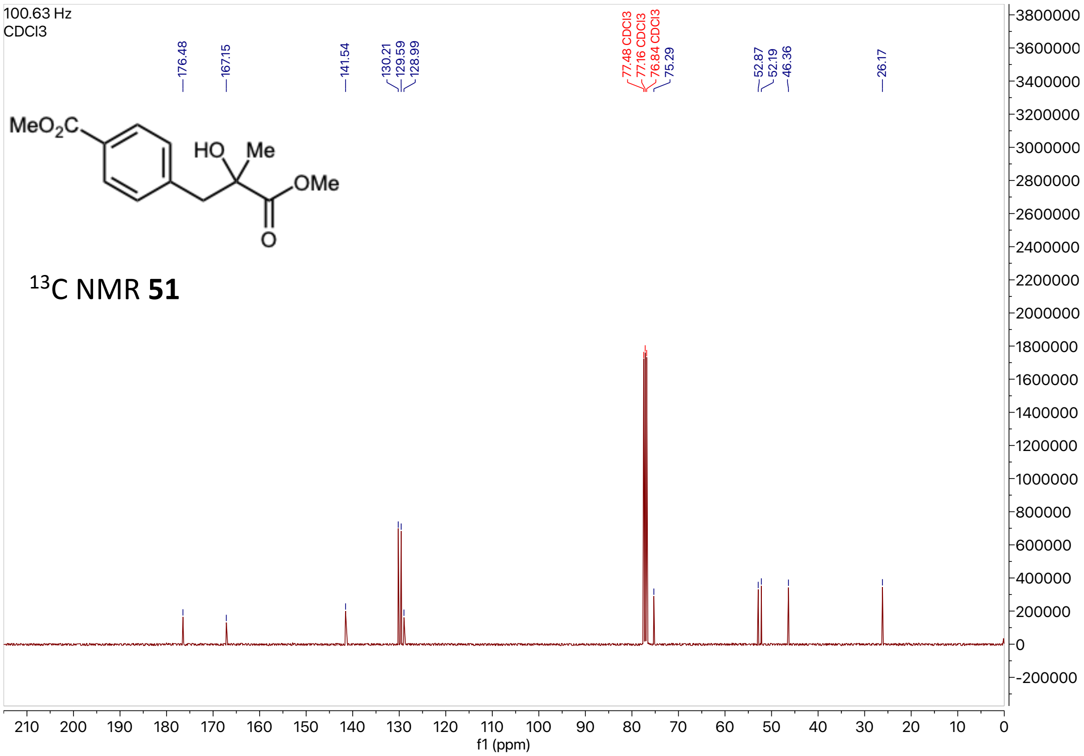
100.63 Hz
CDCl3
MeO2C
HO
Me
OMe
O
13C NMR 51
f1 (ppm)

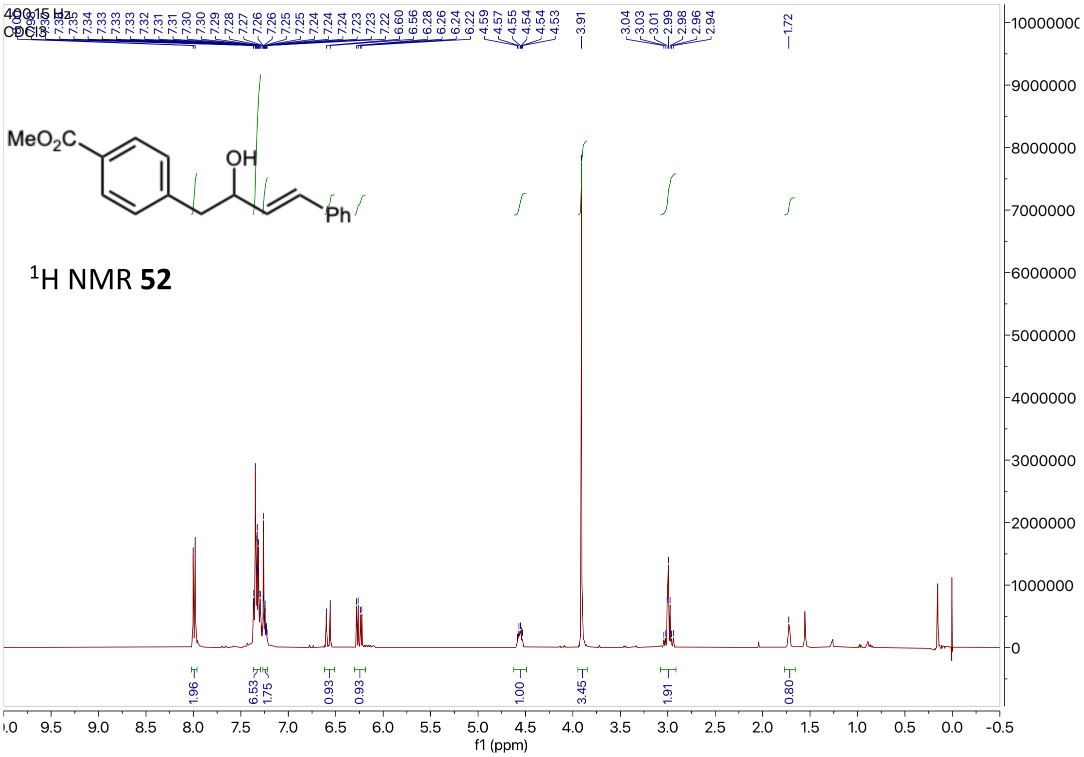
400.15 Hz
CDCl3
MeO2C
OH
Ph
1H NMR 52
f1 (ppm)

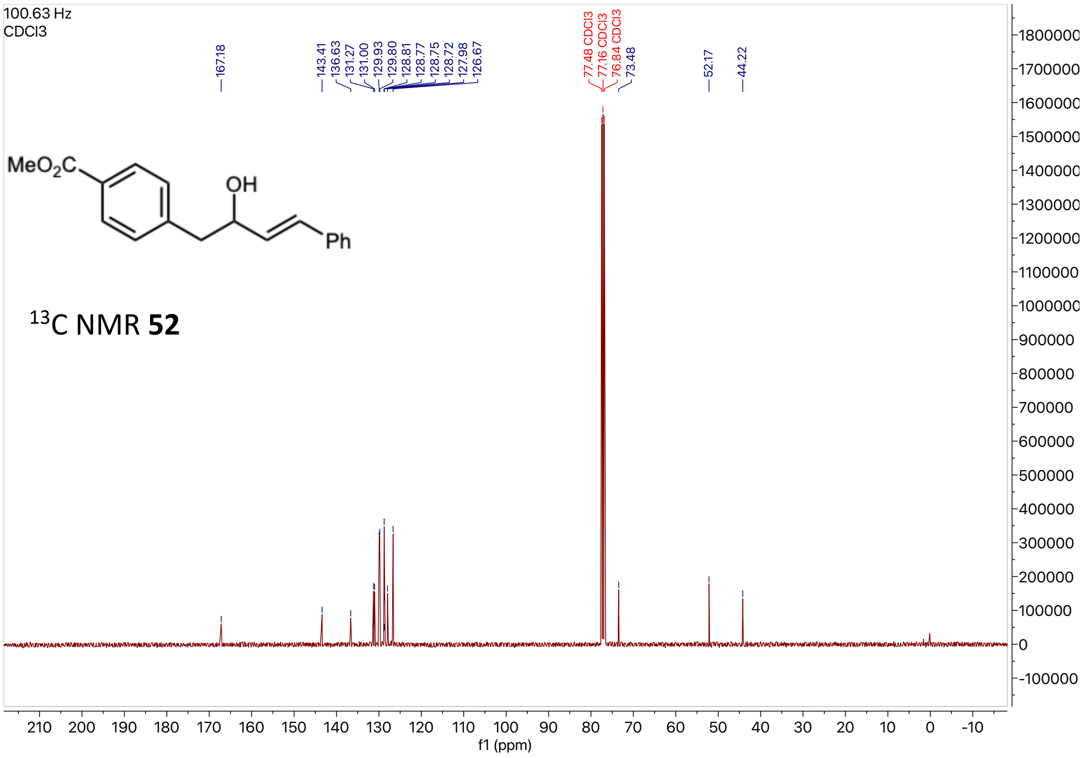
100.63 Hz
CDCl3
MeO2C
OH
Ph
13C NMR 52
f1 (ppm)

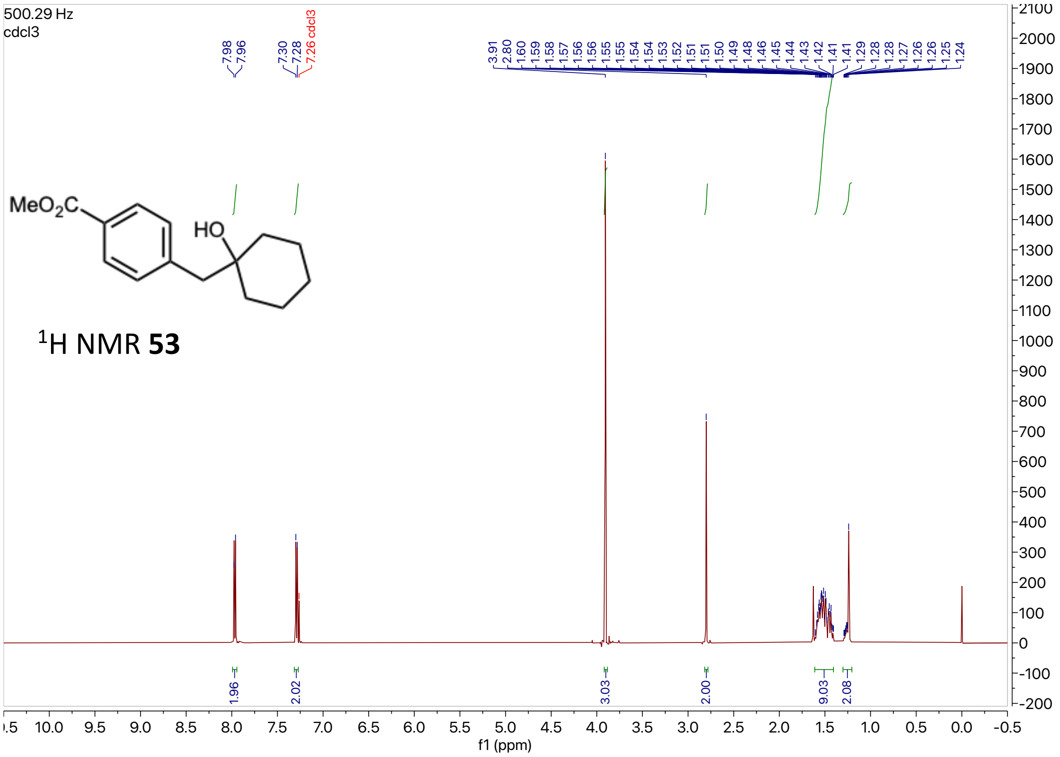

500.29 Hz
cdcl3
7.98
7.96
7.30
7.28
7.26 cdcl3
3.91
2.80
1.60
1.59
1.58
1.57
1.56
1.56
1.55
1.55
1.54
1.54
1.53
1.52
1.51
1.51
1.50
1.49
1.48
1.46
1.45
1.44
1.43
1.42
1.41
1.41
1.29
1.28
1.28
1.27
1.26
1.26
1.25
1.24
MeO2C
HO
1H NMR 53
1.96
2.02
3.03
2.00
9.03
2.08
f1 (ppm)

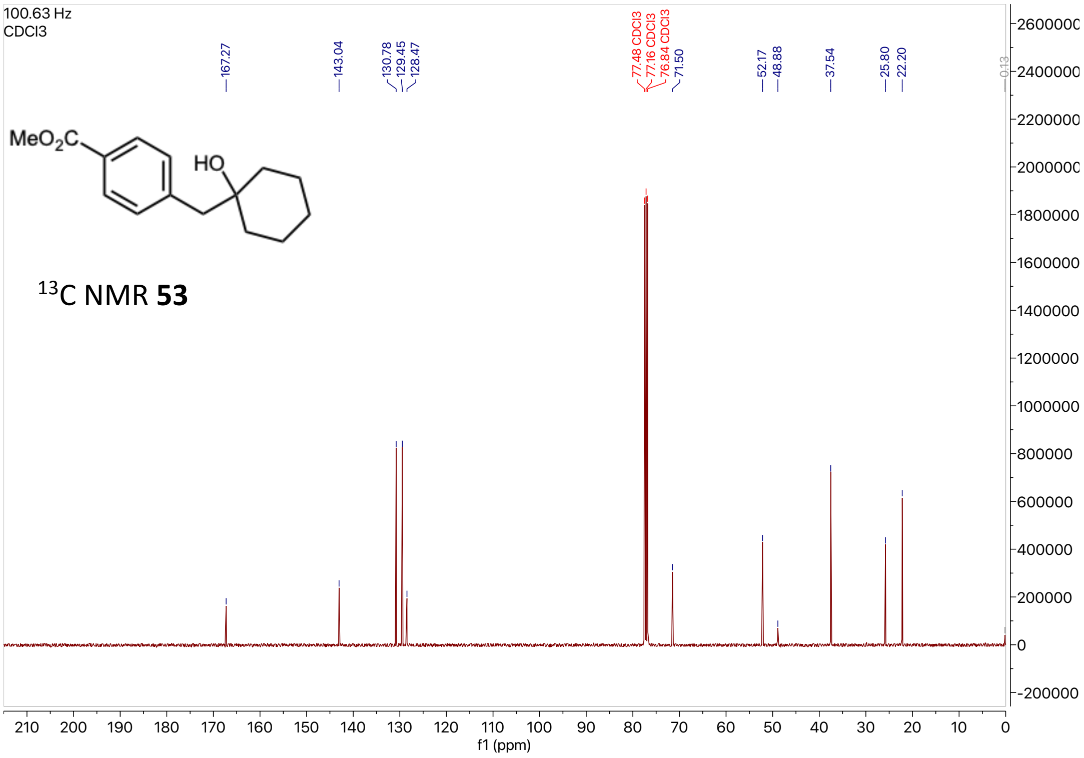

100.63 Hz
CDCl3
167.27
143.04
130.78
129.45
128.47
77.48 CDCl3
77.16 CDCl3
76.84 CDCl3
71.50
52.17
48.88
37.54
25.80
22.20
MeO2C
HO
13C NMR 53
f1 (ppm)

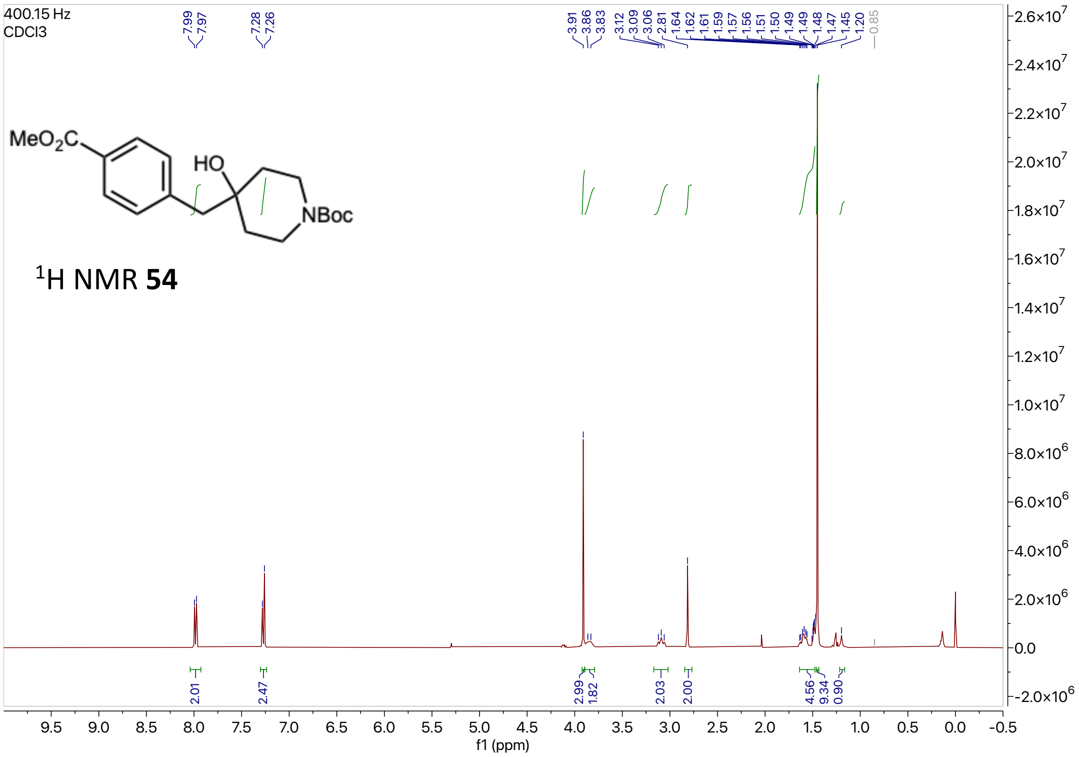

400.15 Hz
CDCl3
MeO2C
HO
NBoc
1H NMR 54
7.99
7.97
7.28
7.26
3.91
3.86
3.83
3.12
3.09
3.06
2.81
1.64
1.62
1.61
1.59
1.57
1.56
1.51
1.50
1.49
1.48
1.47
1.45
1.20
0.85
2.01
2.47
2.99
1.82
2.03
2.00
4.56
9.34
0.90
f1 (ppm)

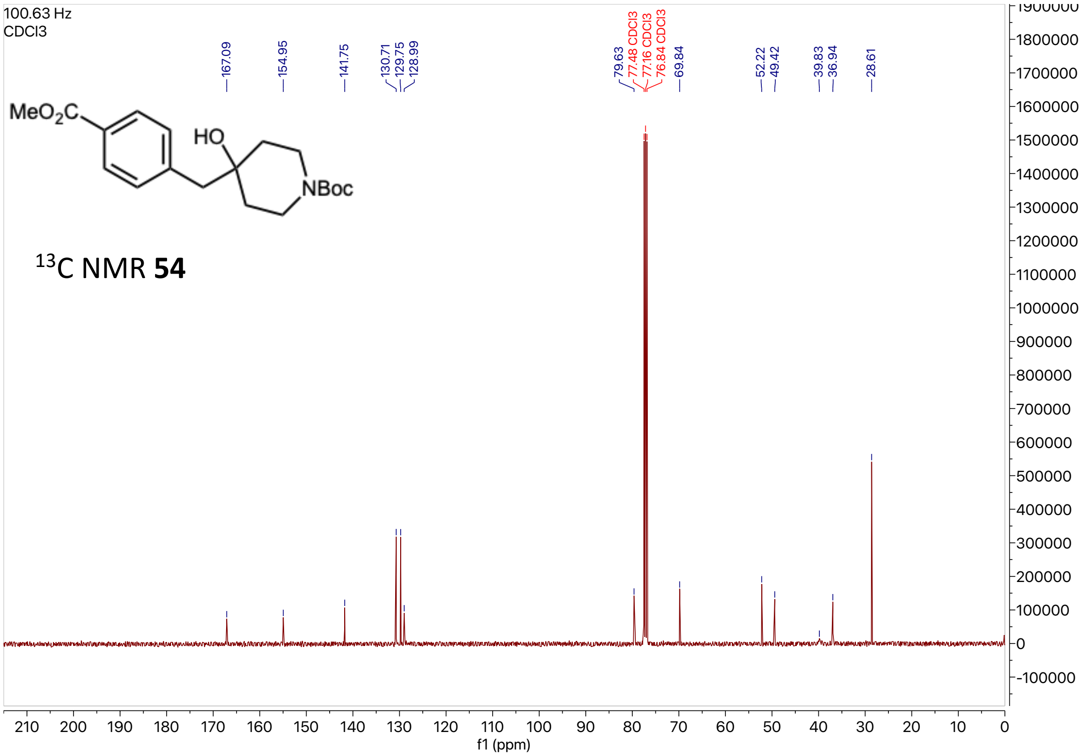

100.63 Hz
CDCl3
MeO2C
HO
NBoc
13C NMR 54
167.09
154.95
141.75
130.71
129.75
128.99
79.63
77.48 CDCl3
77.16 CDCl3
76.84 CDCl3
69.84
52.22
49.42
39.83
36.94
28.61
f1 (ppm)

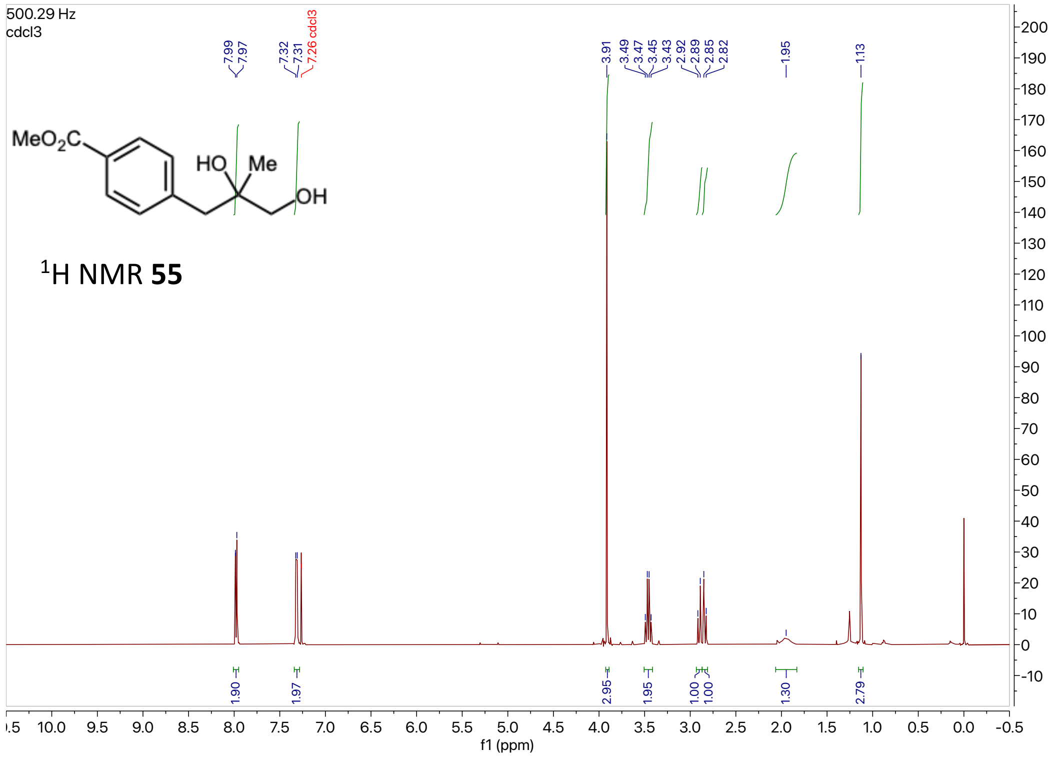
500.29 Hz
cdcl3
7.99
7.97
7.32
7.31
7.26 cdcl3
3.91
3.49
3.47
3.45
3.43
2.92
2.89
2.85
2.82
1.95
1.13
MeO2C
HO
Me
OH
1H NMR 55
1.90
1.97
2.95
1.95
1.00
1.00
1.30
2.79
f1 (ppm)

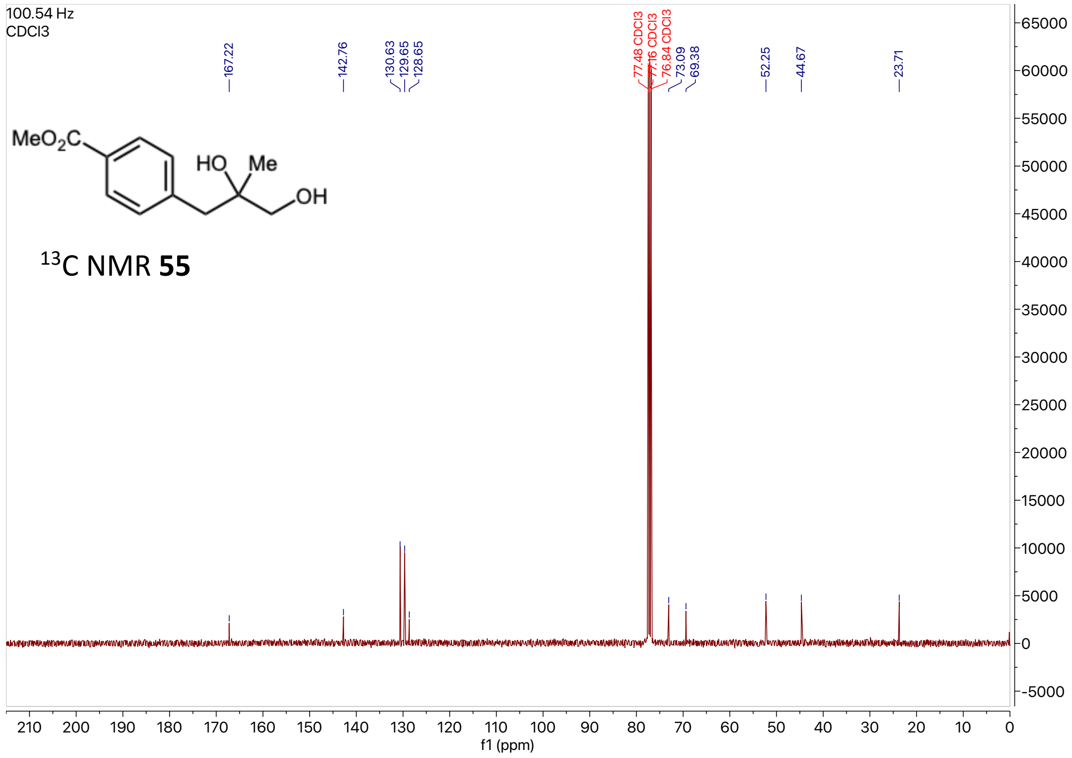
100.54 Hz
CDCl3
167.22
142.76
130.63
129.65
128.65
77.48 CDCl3
77.16 CDCl3
76.84 CDCl3
73.09
69.38
52.25
44.67
23.71
MeO2C
HO
Me
OH
13C NMR 55
f1 (ppm)

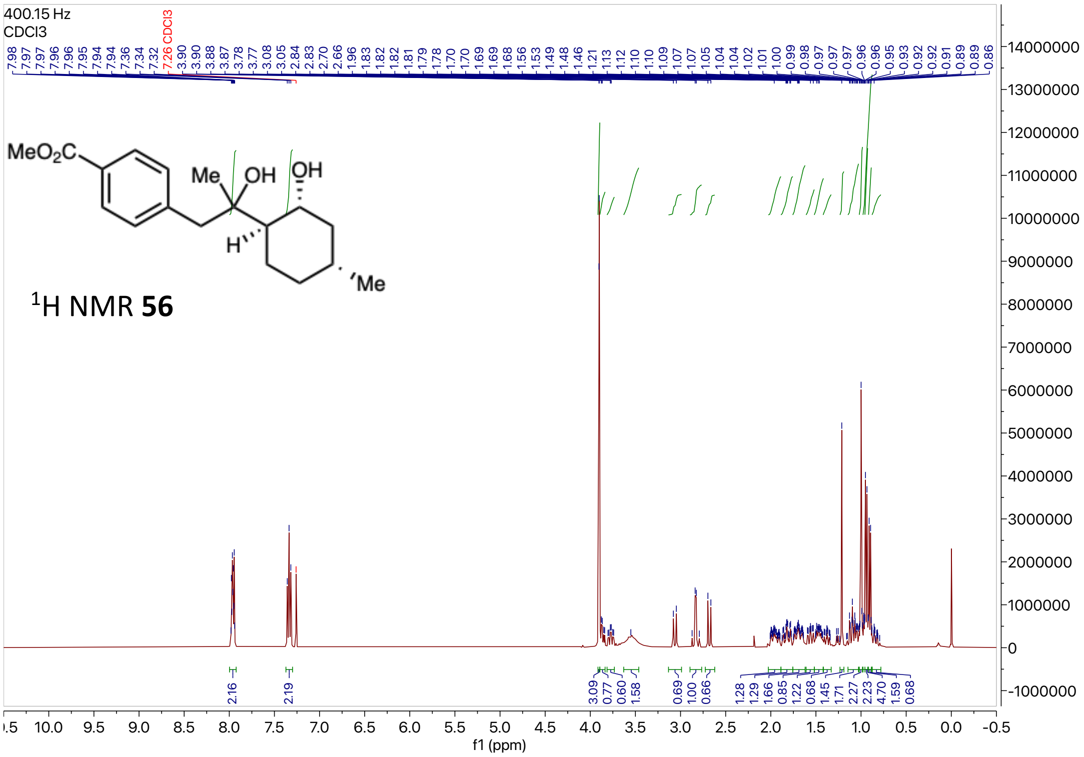
400.15 Hz
CDCl3
1H NMR 56
f1 (ppm)

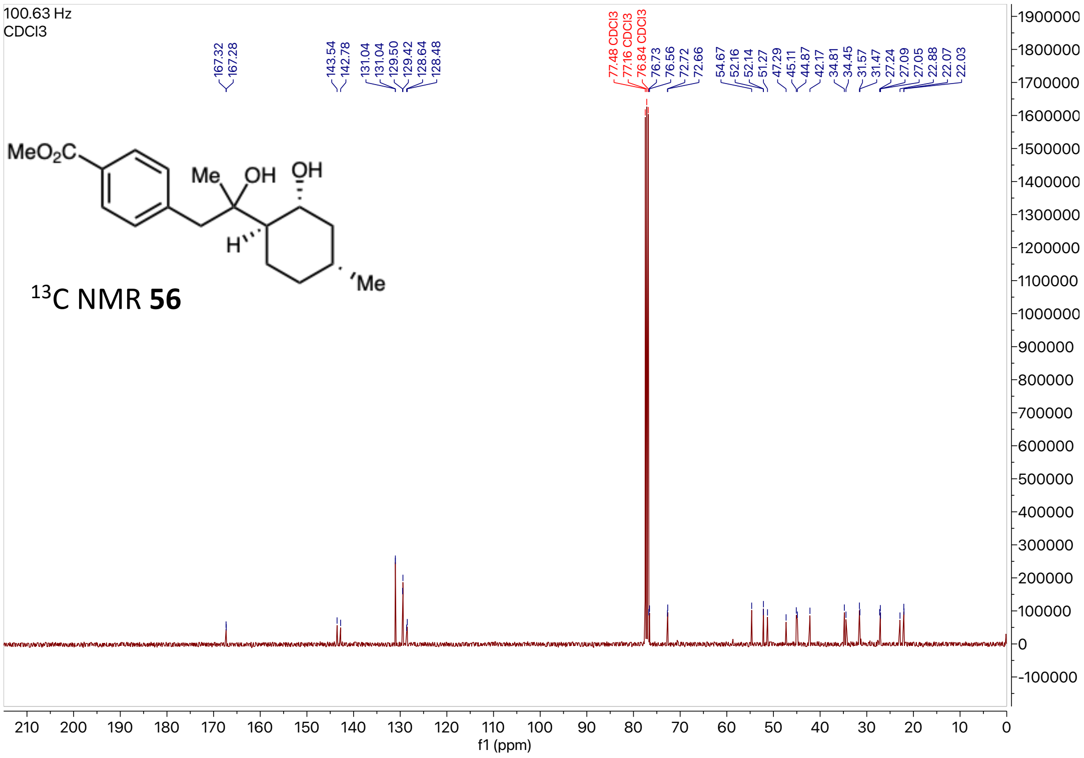
100.63 Hz
CDCl3
13C NMR 56
f1 (ppm)

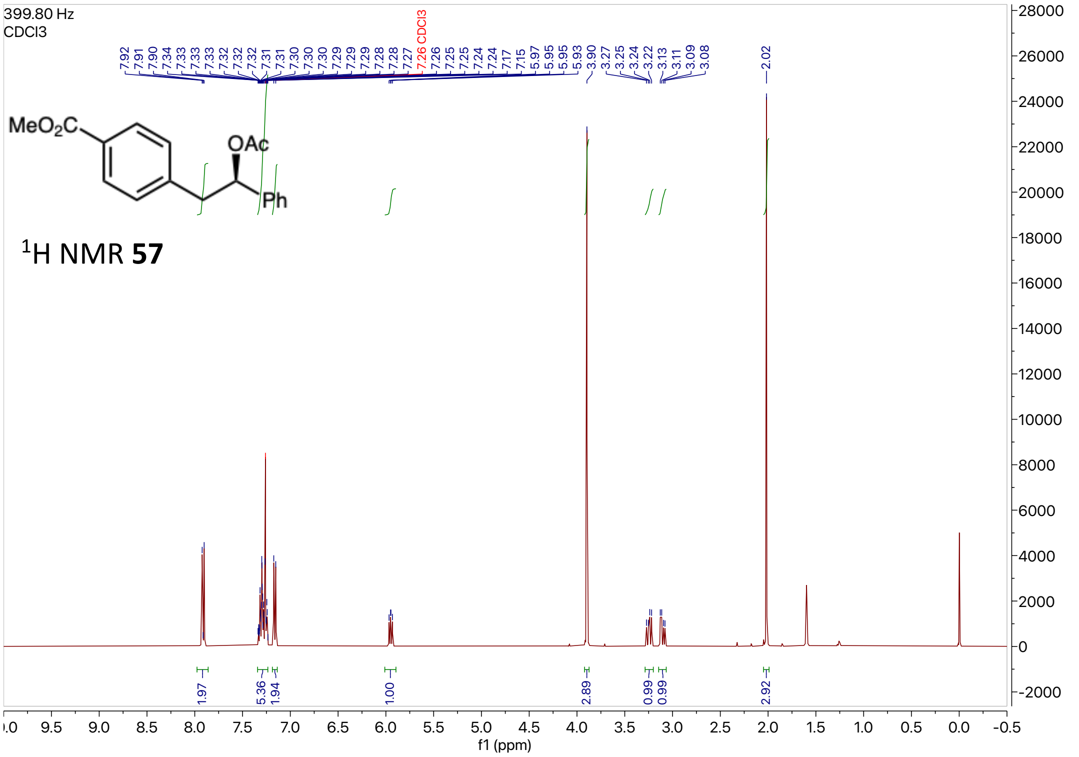
399.80 Hz
CDCl3
7.92 7.91 7.90 7.34 7.33 7.33 7.33 7.32 7.32 7.31 7.31 7.30 7.30 7.29 7.29 7.29 7.28 7.28 7.27 7.26 CDCl3 7.26 7.25 7.25 7.24 7.24 7.17 7.15 5.97 5.95 5.95 5.93 3.90 3.27 3.25 3.24 3.22 3.13 3.11 3.09 3.08 2.02
MeO2C
OAc
Ph
1H NMR 57
1.97 5.36 1.94 1.00 2.89 0.99 0.99 2.92
f1 (ppm)

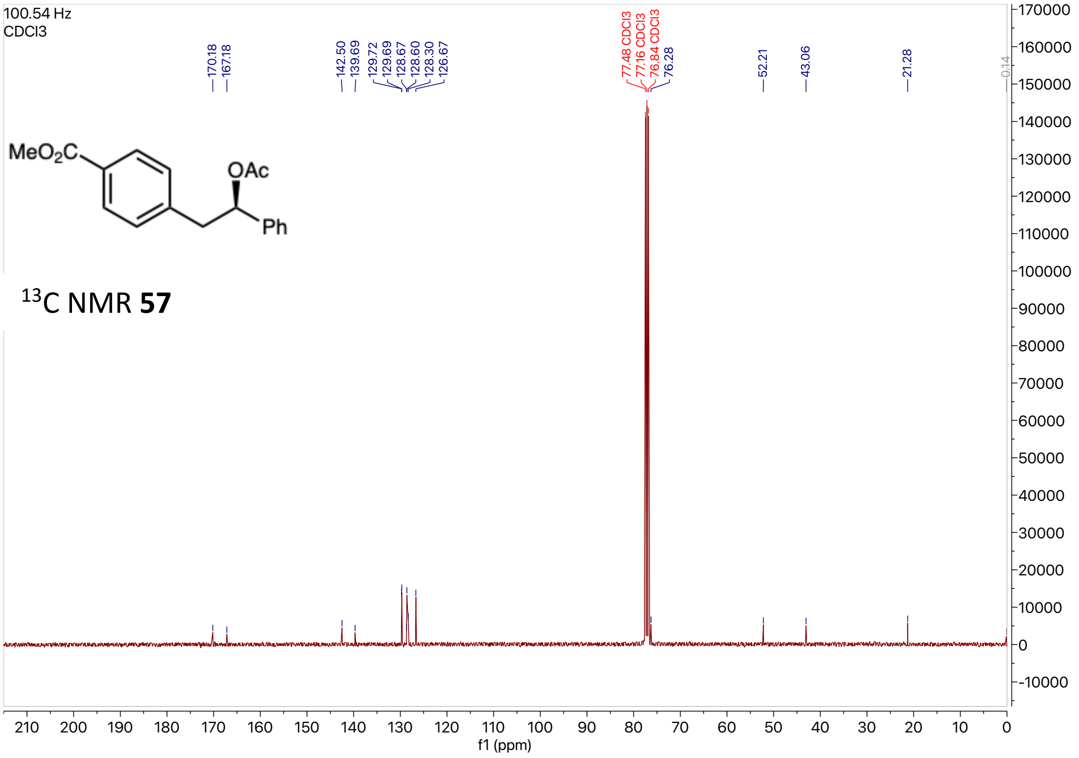
100.54 Hz
CDCl3
170.18 167.18 142.50 139.69 129.72 129.69 128.67 128.60 128.30 126.67 77.48 CDCl3 77.16 CDCl3 76.84 CDCl3 76.28 52.21 43.06 21.28
MeO2C
OAc
Ph
13C NMR 57
f1 (ppm)

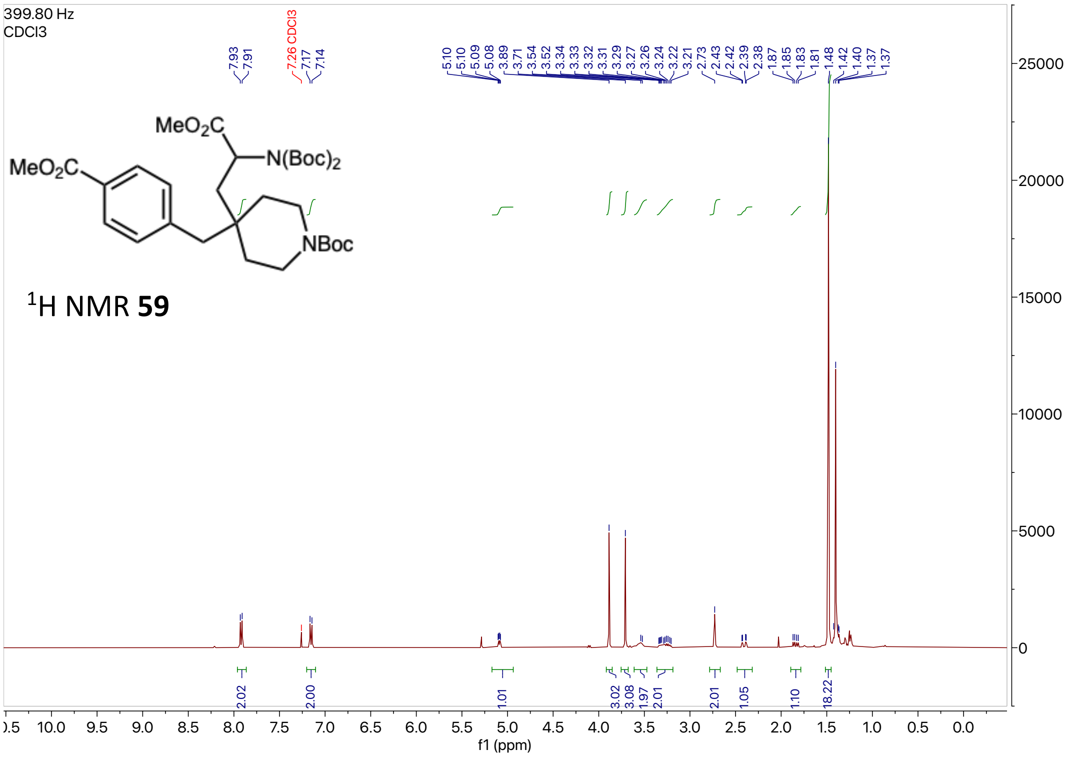

^{1}H NMR **59**

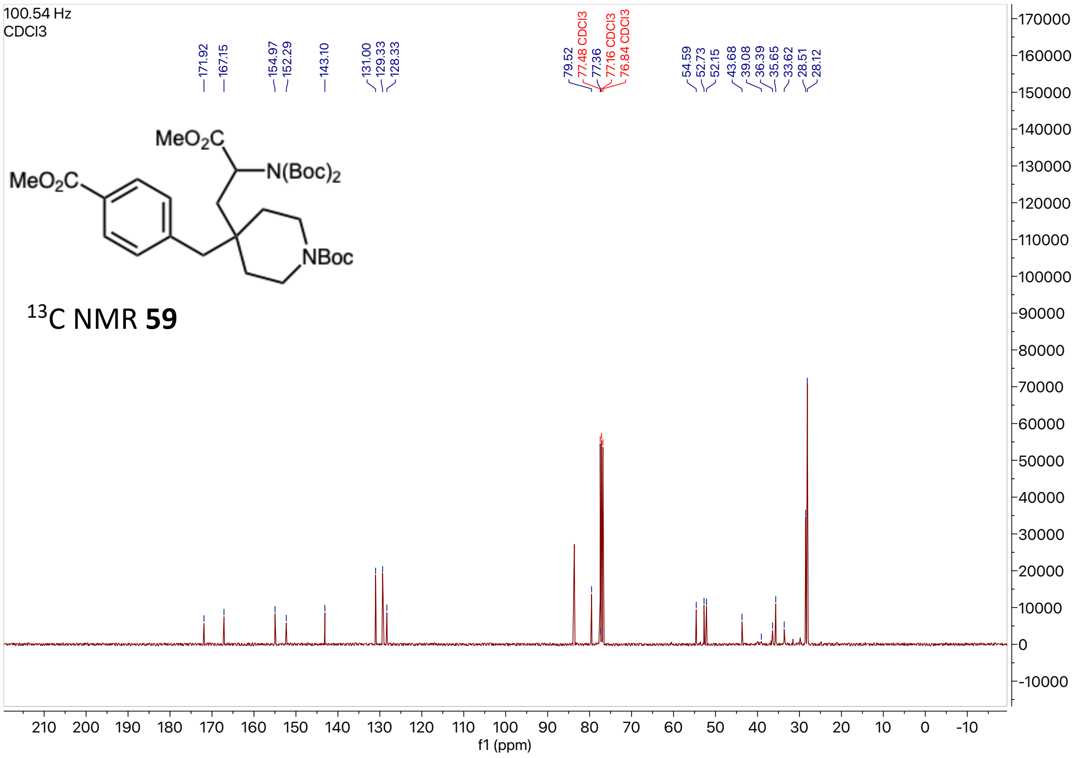

^{13}C NMR **59**

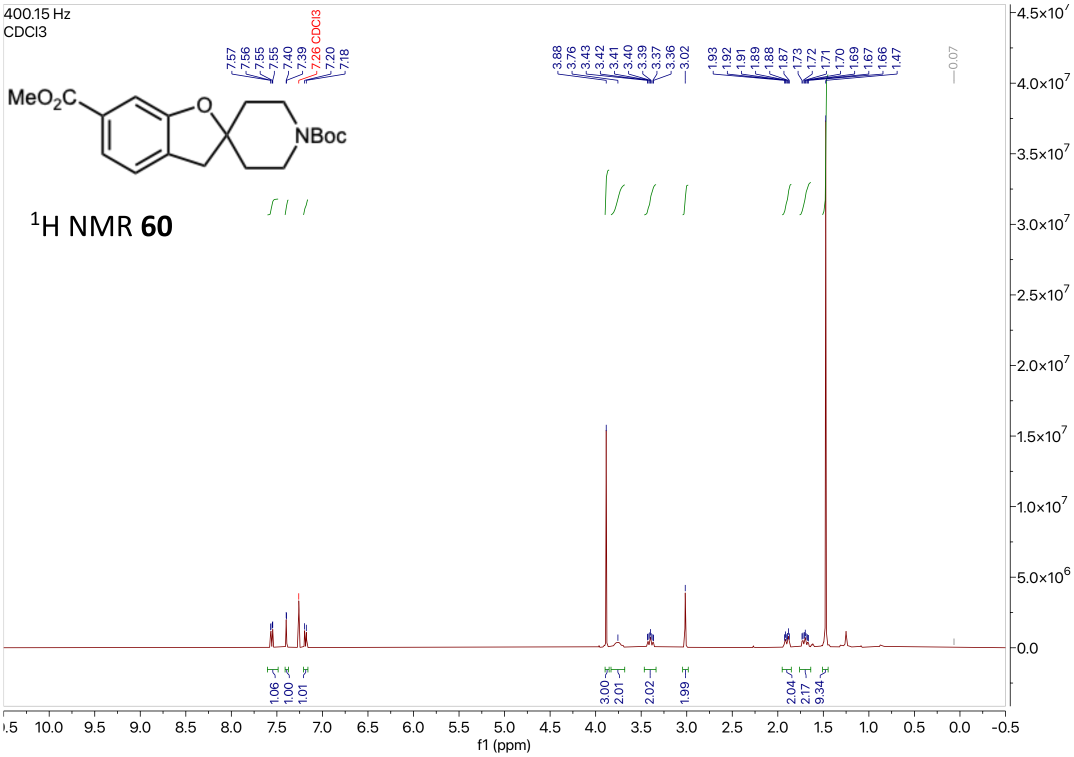
400.15 Hz
CDCl3
7.57
7.56
7.55
7.55
7.40
7.39
7.26 CDCl3
7.20
7.18
3.88
3.76
3.43
3.42
3.41
3.40
3.39
3.37
3.36
3.02
1.93
1.92
1.91
1.89
1.88
1.87
1.73
1.72
1.71
1.70
1.69
1.67
1.66
1.47
0.07
MeO2C
O
NBoc
1H NMR 60
1.06
1.00
1.01
3.00
2.01
2.02
1.99
2.04
2.17
9.34
f1 (ppm)

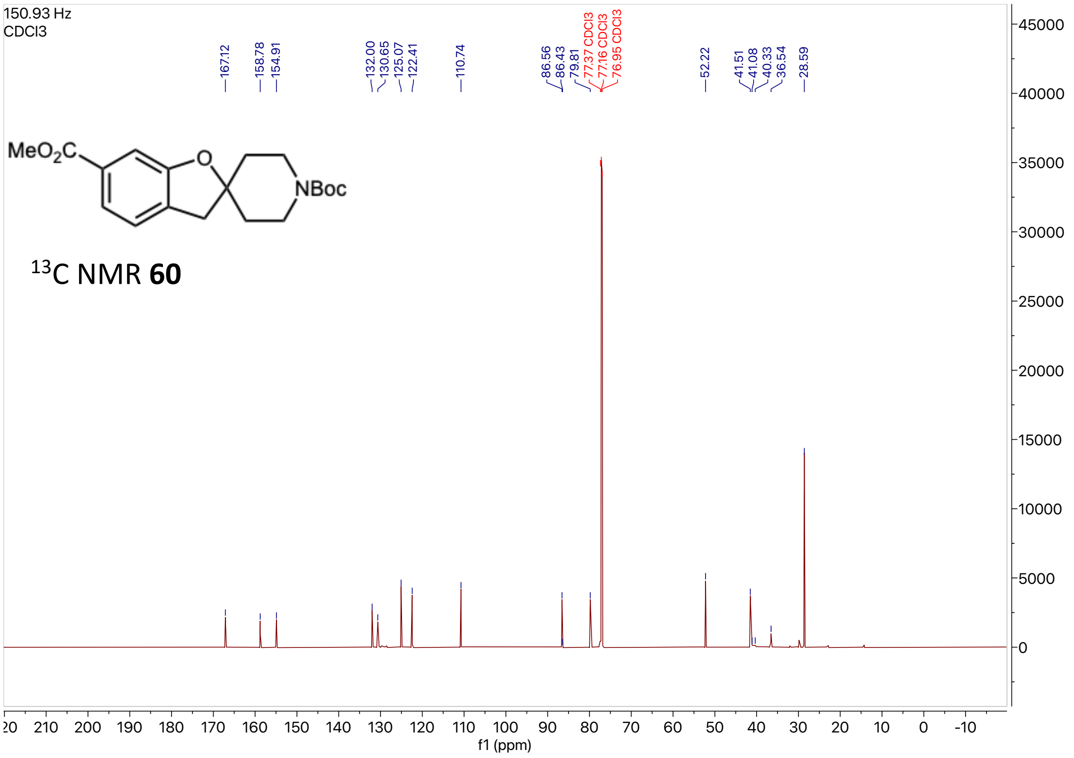
150.93 Hz
CDCl3
167.12
158.78
154.91
132.00
130.65
125.07
122.41
110.74
86.56
86.43
79.81
77.37 CDCl3
77.16 CDCl3
76.95 CDCl3
52.22
41.51
41.08
40.33
36.54
28.59
MeO2C
O
NBoc
13C NMR 60
f1 (ppm)

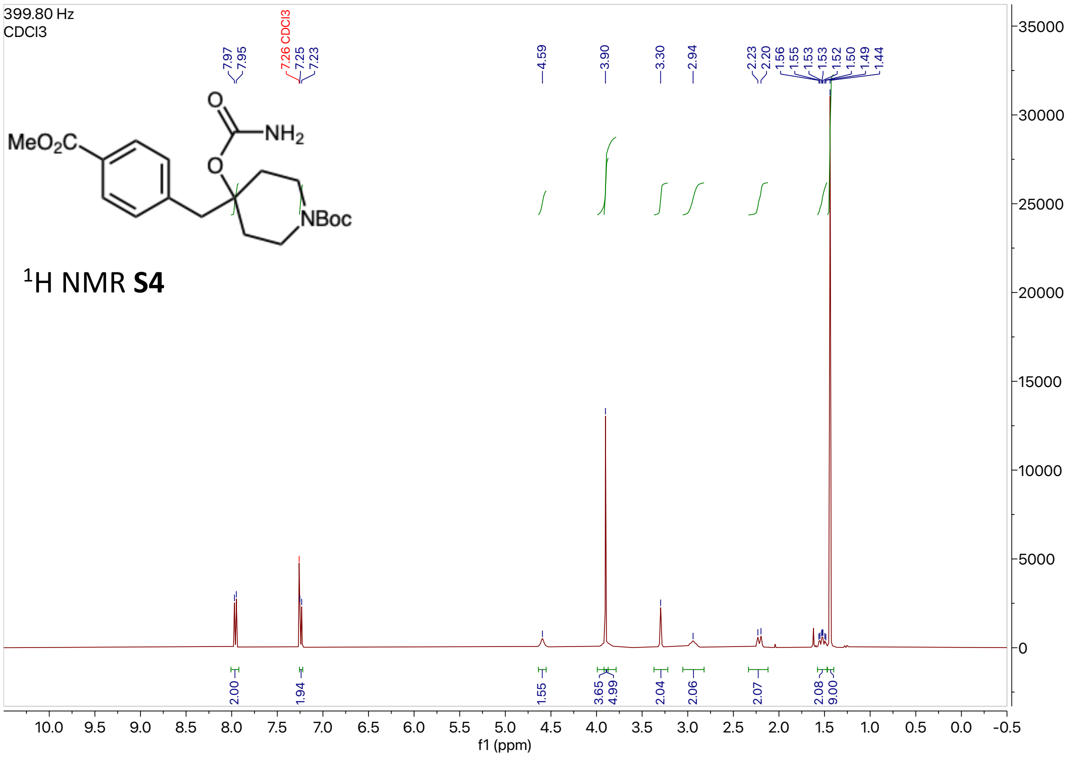

399.80 Hz
CDCl3
7.97
7.95
7.26 CDCl3
7.25
7.23
4.59
3.90
3.30
2.94
2.23
2.20
1.56
1.55
1.53
1.53
1.52
1.50
1.49
1.44
MeO2C
NH2
NBoc
1H NMR S4
2.00
1.94
1.55
3.65
4.99
2.04
2.06
2.07
2.08
9.00
f1 (ppm)

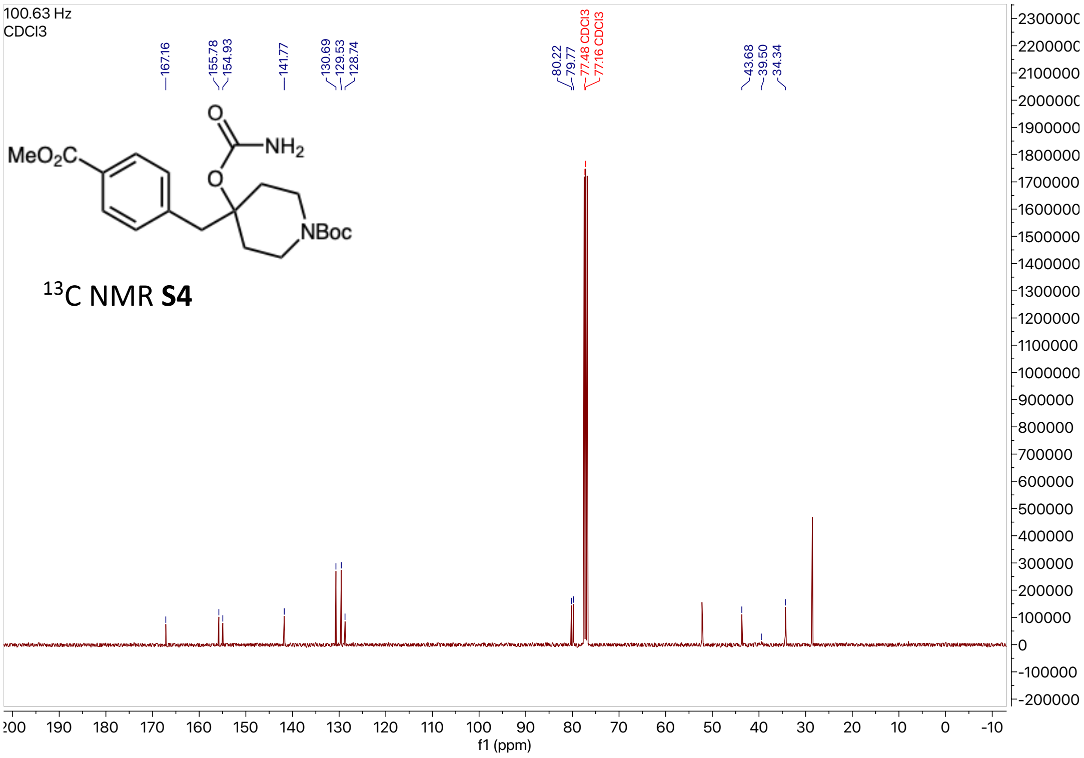

100.63 Hz
CDCl3
167.16
155.78
154.93
141.77
130.69
129.53
128.74
80.22
79.77
77.48 CDCl3
77.16 CDCl3
43.68
39.50
34.34
MeO2C
NH2
NBoc
13C NMR S4
f1 (ppm)

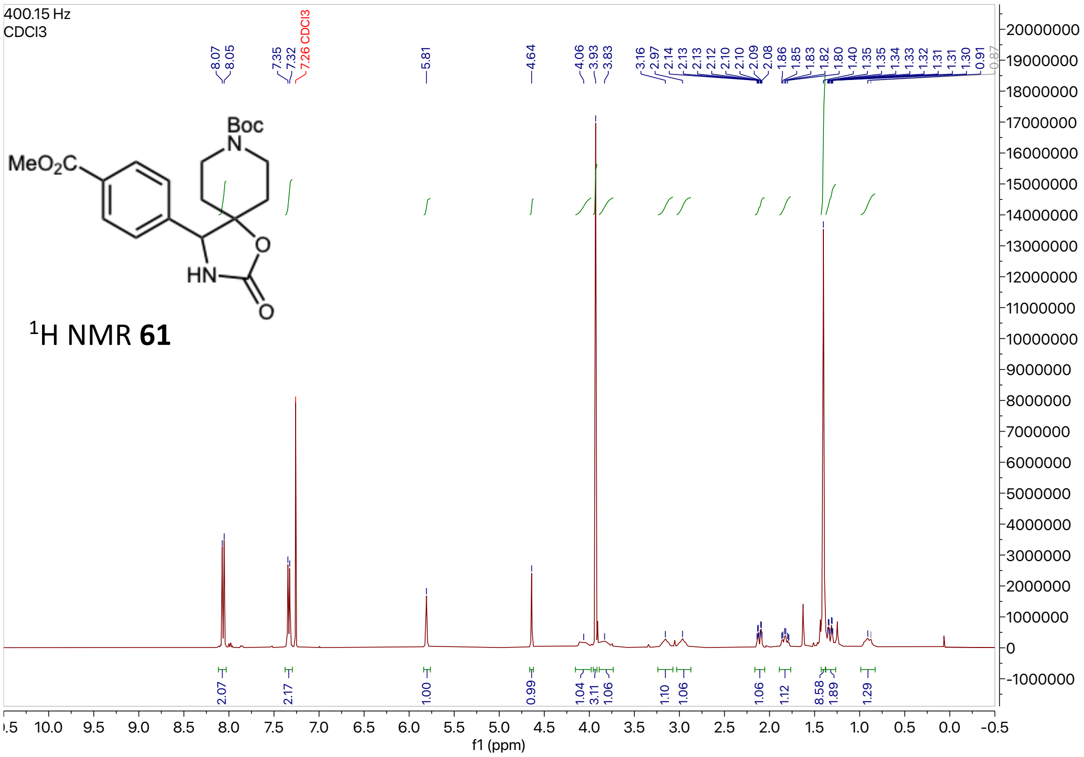

400.15 Hz
CDCl3
1H NMR 61
MeO2C
Boc
N
O
HN
O
f1 (ppm)

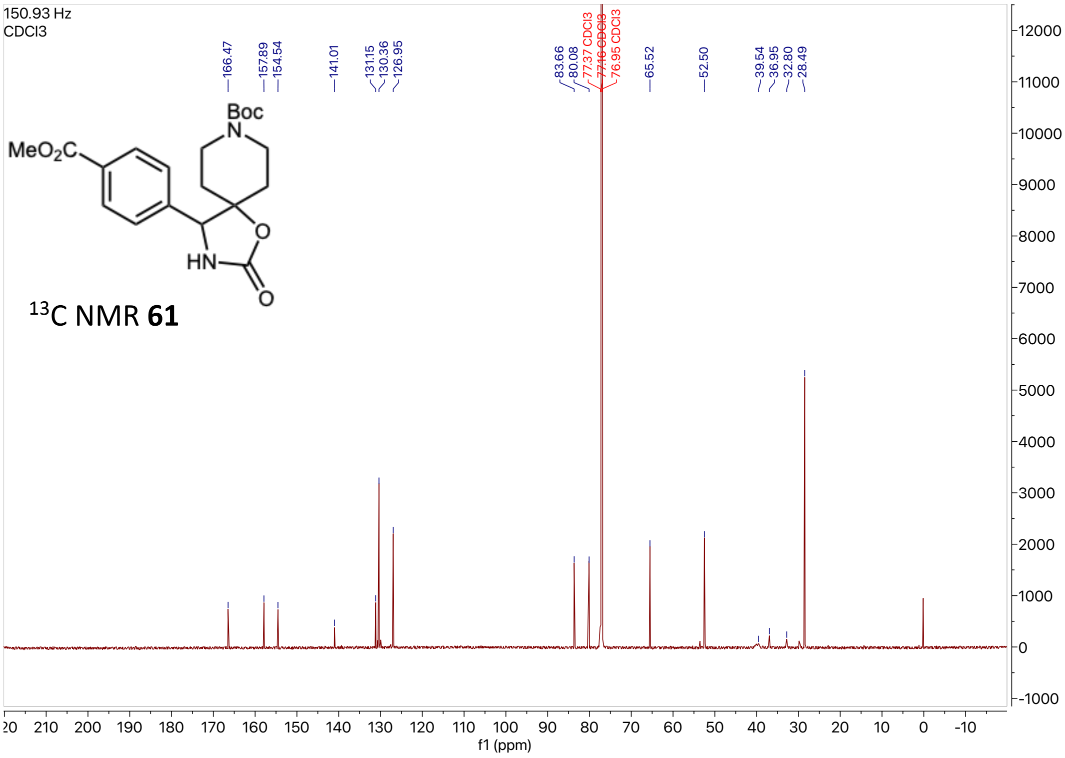

150.93 Hz
CDCl3
13C NMR 61
MeO2C
Boc
N
O
HN
O
f1 (ppm)
